# Integrated transcriptomic and proteomic analysis of the immune response in *Hyalomma anatolicum* to bacterial invasion

**DOI:** 10.3389/fimmu.2025.1576721

**Published:** 2025-09-04

**Authors:** Jianlong Li, Tingxiang Luo, Zhen Ma, Depeng Yang, Sen Yang, Yongchang Li, Bayinchahan Gailike, Qingyong Guo, Zhengxiang Hu, Ercha Hu

**Affiliations:** ^1^ College of Veterinary Medicine, Xinjiang Agricultural University, Urumqi, China; ^2^ Xingjiang Key Laboratory of New Drug Research and Development for Herbivorous, Urumqi, China; ^3^ College of Grassland Science, Xinjiang Agricultural University, Urumqi, China; ^4^ Veterinary Medicine Postdoctoral Research Station of Xinjiang Agricultural University, Urumqi, China

**Keywords:** *Hyalomma anatolicum*, immune response, TMT-labeled quantitative proteomics, RNA-seq, immune defense mechanism

## Abstract

**Background:**

*Hyalomma anatolicum* is a multi-host ectoparasite that carries and transmits a variety of zoonotic pathogens. Understanding the immune response of ticks to bacterial infections is of research significance for deciphering their immune defense mechanisms and harnessing tick - derived molecules.

**Methods:**

In the current study, transcriptomic and proteomic analyses on *H. anatolicum* injected with *Staphylococcus aureus* (SA group), *Proteus mirabilis* (PM group) or phosphate buffered saline (PBS group) were performed.

**Results:**

In pairwise comparisons among the experimental groups, we identified 9,776 (SA/PBS), 10,230 (PM/PBS), and 1,309 (SA/PM) differentially expressed genes (DEGs), as well as 175 (SA/PBS), 277 (PM/PBS), and 223 (SA/PM) differentially expressed proteins (DEPs), respectively. Subsequent KEGG pathway analysis revealed that these DEGs and DEPs were significantly enriched in a range of pertinent pathways, including the immune system and apoptosis, Toll and IMD signaling pathways, MAPK signaling pathway, and NF - κB signaling pathway. The RT - qPCR detection data exhibited a concordant trend with the RNA - seq data, indicating a substantial alignment in the observed results. Notably, the defensin and lectin gene families emerged as potentially pivotal components within the innate immune defense system of ticks.

**Conclusion:**

Overall, in this study, genes, proteins, and signaling pathways integral to the immune defense of *H. anatolicum* were identified, offering substantial potential for future research focused on harnessing its intricate immune defense mechanisms for antimicrobial applications.

## 1 Introduction

Ticks are commonly found across various regions, and their role in transmitting numerous pathogens has great interest among researchers in the fields of public health and veterinary medicine (1), and this has resulted in detailed investigations and a surge of research activities being conducted (2, 3). These parasites are commonly found in various ecosystems, including grasslands and forest areas, and they are competent in transmitting a range of pathogens to humans and livestock, such as bacteria, viruses, and protozoa (4). *Hyalomma anatolicum* is a hard tick of considerable medical significance, with its exclusive dependency on the blood of host animals as its only source of nutrition (5). This tick species is primarily distributed in Central Asia, Southern Asia, and several countries in Eastern Europe (6). In China, *H. anatolicum* primarily presents in the northwest region, particularly with notable populations observed in Xinjiang Uygur Autonomous Region (7). *H. anatolicum* is a competent vector for various pathogenic microorganisms, often causing significant economic losses in the local animal husbandry (8).

Ticks possess unique abilities to survive and reproduce in various environments, and their adaptive physiology and innate immune system serve as crucial defenses against pathogen invasion (9, 10). Conducting in-depth research into the complex innate immune system of ticks can help us uncover their survival strategies and simultaneously provide valuable insights into potential defense mechanisms against pathogenic organisms. When compared to vertebrates, the immune systems of ticks and other invertebrates are more rudimentary, primarily relying on innate immune mechanisms to combat challenges from external pathogens (11, 12). The innate immunity of ticks, similar to other invertebrates, relies on the coordinated action of both humoral and cellular immune responses, where cellular defenses encompass phagocytosis, encapsulation, and nodulation of foreign elements, while humoral defenses hinge on a variety of pattern recognition proteins and effector molecules (13). Ticks possess various pattern recognition receptors (PRRs), including lectins, Toll receptors, IMD (immune deficiency) receptors, and scavenger receptors (14, 15). When microorganisms invade, the host’s PRRs recognize pathogen-associated molecular patterns (PAMPs) on the surface of these pathogens, triggering specific immune responses (11). In response to the invasion of pathogens, tick immune cells activate and produce a spectrum of antimicrobial peptides (AMPs), such as defensins, microplusin, and tick venom proteins, which demonstrate antibacterial activity against a diverse range of bacteria, fungi, and certain parasites, as part of their immune system’s defense mechanisms (16–18).

Transcriptomic and proteomic sequencing allow for the rapid acquisition of global gene and protein expression data from specific time points and tissues of a biological organism. These techniques enable the study of gene and protein function and structure at a holistic level, revealing specific biological processes (19, 20). The aim of this study is to enrich the genetic and proteomic sequence information of *H. anatolicum* and to analyze the changes in mRNA and proteins related to immune defense in response to bacterial challenges. To achieve this, we performed a comparative analysis of transcriptomics and proteomics on *H. anatolicum* samples treated with *Proteus mirabilis* (PM group), *Staphylococcus aureus* (SA group), and phosphate-buffered saline (PBS group), using TMT - based quantitative proteomics and RNA - seq methods.

## 2 Methods

### 2.1 Experimental samples and ethical statements

*H. anatolicum* used in the experiment were artificially reared ticks preserved in the College of Veterinary Medicine, Xinjiang Agricultural University. The experimental conditions for the rearing and storage in the laboratory were maintained as follows: an ambient air temperature of 25 ± 2 °C, a relative humidity of 85% ± 5%, and a photoperiod consisting of 15 hours of illumination and 9 hours of darkness. Gram-positive *S. aureus* and Gram-negative *P. mirabilis* strains were isolated from the purulent exudates collected from the sites on the ears of experimental rabbits where *H. anatolicum* had attached and engaged in blood - feeding. All animal procedures were carried out in accordance with the guidelines in the Document 2023025 approved by the Animal Ethics Committee of Xinjiang Agricultural University.

### 2.2 Experimental design

To investigate the gene expression profiles of *H. anatolicum* in response to bacterial challenges, transcriptome and proteome sequencing were conducted in this study. Specifically, female *H. anatolicum* ticks, collected from the same breeding experiment two weeks post - molting, were first immersed in sterile phosphate - buffered saline (PBS) solution and gently agitated for 15 s to clean their surfaces for three times. After thorough drying on sterile medical gauze, the ticks were positioned under a dissecting microscope with their ventral side exposed upwards. Each tick was then individually injected with either *S. aureus* suspended in phosphate-buffered saline (500 nL containing 10^7^ CFU) or *P. mirabilis* suspended in PBS (500 nL containing 10^7^ CFU), and a small amount of sterile petrolatum was picked up with a sterile needle and quickly applied to seal the wound upon needle withdrawal. This was accomplished through a puncture into their fourth coxal segment using a precision microsyringe (Hamilton Bonaduz, LTD, Switzerland). The control group received an equivalent volume of PBS (500 nL, 10 mM) to allow for comparative analysis of responses during bacterial invasion (21).

### 2.3 Sample preparation for sequencing

After injection, each tick was individually positioned in a 5 ml Eppendorf tube for observation. Following a 24 - hour period, those displaying robust vitality and normal mobility were chosen (survival rate > 95% among punctured ticks), and placed into cryovials. For subsequent sequencing sample preparation, each sample comprised a group of ten ticks pooled together, with three biological replicates for each experimental group. The samples were swiftly submerged in liquid nitrogen for freezing and subsequently stored at -80 °C for future use.

### 2.4 *De novo* transcriptome sequencing and data analysis

#### 2.4.1 Total RNA extraction and cDNA library construction

The extraction of total RNA was conducted using Trizol reagent (Invitrogen, California, USA) according to the manufacturer’s instructions. The concentration and purity of the total RNA were assessed using a NanoDrop (Thermo Scientific NanoDrop 2000, USA), while the integrity was evaluated using a 2100 Bioanalyzer (Agilent, California, USA) with the RNA 6000 Nano kit. Poly(A) RNA was purified using Poly - T magnetic beads from 5 μg of total RNA, with one round of extraction repeated.

The purified mRNA was randomly fragmented using divalent cations through an ionic disruption process. Fragmented mRNA served as the template, with random oligonucleotides acting as primers for cDNA synthesis. The purified double - stranded cDNA underwent end - repair, A - tailing, and ligation of sequencing adapters. cDNA fragments of 400–500 bp were selected using AMPure XP beads for PCR amplification. The PCR products were then purified using AMPure XP beads, resulting in the final library.

The quality of the library was assessed using an Agilent 2100 Bioanalyzer with the Agilent High Sensitivity DNA Kit. The total concentration of the library was determined using Pico Green, while the effective library concentration was quantified using qPCR. After normalization, the multiple DNA libraries were mixed in equal volumes. The mixed library was then diluted and quantified before being sequenced on an Illumina sequencing platform in PE150 mode.

#### 2.4.2 Data quality control

Following sequencing, image files were obtained and converted into raw data in FASTQ format using software provided by the sequencing platform. These raw data, also known as off - machine data, contained a small number of reads with sequencing adapters or low quality. To ensure the quality of subsequent information analysis, raw reads were filtered to obtain clean reads. The data processing steps included using fastp v.0.22.0 to remove sequences with adapters at the 3’ end and to discard low - quality reads, which were defined as reads where more than 50% of the bases had a quality score of Qphred ≤ 20. All subsequent analyses were based on high - quality clean data.

#### 2.4.3 Transcript assembly and gene functional annotation

Using Trinity v.2.15.1, the clean reads were assembled into unigenes for subsequent analysis (22). These unigenes were then subjected to gene functional annotation. The databases utilized for gene functional annotation included NR (NCBI non-redundant protein sequences), GO (Gene Ontology), KEGG (Kyoto Encyclopedia of Genes and Genomes), EggNOG (evolutionary genealogy of genes: Non-supervised Orthologous Groups), Swiss - Prot and Pfam (Protein Family Database). Additionally, Animal TFDB (Animal Transcription Factor DataBase) was used to predict transcription factors (TFs) among the unigenes obtained from sequencing.

#### 2.4.4 Identification of differentially expressed genes and functional enrichment analysis

Using the transcriptome expression quantification software RSEM v.2.15, the assembled unigene sequences were employed as the reference. The clean reads from each sample were aligned to the reference sequences. Subsequently, the number of reads mapped to each gene in each sample was counted, and the fragments per kilobase of transcript per million mapped (FPKM) values for each gene were calculated (23). Differential expression analysis between the two comparison groups was conducted using DESeq v1.38.3. DESeq was utilized for differential gene expression analysis, with genes considered differentially expressed if they met the criteria of |log2 FoldChange| > 1 and a significance of *P* < 0.05.

Using topGO v.2.50.0 for GO enrichment analysis, we calculate *P* - values through the hypergeometric distribution method, with a significance threshold set at *P* < 0.05. This process identifies significantly enriched GO terms among differentially expressed genes, thereby delineating the primary biological functions these genes perform. Additionally, we employ clusterProfiler v.4.6.0 for KEGG pathway enrichment analysis, with a particular emphasis on significantly enriched pathways at *P* < 0.05.

#### 2.4.5 Analysis of protein-protein interaction networks for differential genes

To gain deeper insights into the defense mechanisms of *H. anatolicum* following bacterial treatment, a protein-protein interaction (PPI) network was constructed based on the identified differentially expressed genes. PPI pairs were then screened from the STRING database (https://string-db.org/), where both end nodes corresponded to differential genes with a score exceeding 0.95, for conducting PPI analysis to unravel the interactions among the genes of interest.

#### 2.4.6 Validation by real-time quantitative PCR

The expression levels of differentially expressed genes identified from transcriptome sequencing data were validated using RT - qPCR. The cDNA samples from ticks used in the RT - qPCR experiments were obtained from female adult ticks of *H. anatolicum*, punctured and processed from a single batch, utilizing the tick sample preparation as described before.

Reverse transcription was then performed using the Fastking RT cDNA Synthesis Kit (TianGen Biotech, LTD, China). The RT - qPCR experiments were conducted using the cDNA, gene-specific primers, and QuantiNova SYBR^®^Green PCR Kit (Qiagen N.V, LTD, Germany). The reaction program included an initial denaturation at 95 °C for 2 min, followed by 40 cycles of denaturation at 95 °C for 15 s and annealing/extension at 60 °C for 35 s. Elongation factor EF-1 alpha (ELF1α) was selected as the reference gene (24). The fold change of the identified genes was calculated using the relative quantification method 2^^-△△^CT (25). The primer sequences and amplification system are presented in **Supplementary Table S1**.

### 2.5 TMT-labeled quantitative proteomics sequencing and data analysis

#### 2.5.1 Protein preparation and TMT-labeled quantitative analysis

The SDT buffer (comprising 4% SDS, 100 mM Tris-HCl, 1 mM DTT, adjusted to pH 7.6) was utilized for sample lysis and protein extraction. Protein quantification was subsequently performed using the BCA Protein Assay Kit sourced from Bio-Rad, USA. Trypsin digestion adhered to the filter-aided sample preparation (FASP) protocol outlined by Matthias Mann. The digested peptides from each sample underwent desalting through C18 columns (Empore™ SPE Cartridges C18, standard density, I.D. 7 mm, 3 ml volume, Sigma), followed by concentration via vacuum centrifugation and resuspension in 40 µl of 0.1% (v/v) formic acid. Twenty micrograms of protein from each sample were mixed with 5 X loading buffer and boiled for 5 min. Proteins were then separated on a 12.5% SDS - PAGE gel at a constant current of 14 mA for 90 min. Protein bands were visualized through Coomassie Blue R - 250 staining. One hundred micrograms of peptide mixtures from each sample were labeled with TMT reagents (Thermo Fisher Scientific Inc, USA) according to the manufacturer’s instructions provided by Thermo Scientific.

#### 2.5.2 High-pH reverse-phase separation and LC - MS/MS data acquisition

The labeled peptides were separated using a high - pH reversed-phase peptide separation kit (Thermo Fisher Scientific Inc, USA). The dried peptide mixture was resuspended and acidified with a 0.1% TFA solution, then loaded onto a balanced high - pH reversed - phase spin column. The peptides bound to the hydrophobic resin under aqueous conditions and were desalted by washing the column with low - speed centrifugation. A stepped gradient of volatile high - pH elution solution with increasing acetonitrile concentrations was then applied to the column, eluting the bound peptides into 10 distinct fractions collected by centrifugation. The collected fractions were desalted using C18 cartridges (Empore™ SPE Cartridges C18, standard density, I.D. 7 mm, 3 ml volume, Sigma) and concentrated by vacuum centrifugation.

LC - MS/MS analysis was performed on a Q Exactive mass spectrometer (Thermo Fisher Scientific Inc,USA), coupled with an Easy nLC system, over a duration of 60/90 minutes. Peptides were loaded onto a reversed - phase trap column (Thermo Scientific Acclaim PepMap100, 100 μm × 2 cm, nanoViper C18), which was connected to a C18 reversed - phase analytical column (Thermo Scientific Easy Column, 10 cm length, 75 μm inner diameter, 3 μm resin) in buffer A (0.1% formic acid). Separation was achieved using a linear gradient of buffer B (84% acetonitrile and 0.1% formic acid) at a flow rate of 300 nl/min, controlled by IntelliFlow technology. The mass spectrometer operated in positive ion mode. MS data was acquired using a data - dependent top 10 method, dynamically selecting the most abundant precursor ions from a survey scan for HCD fragmentation. The automatic gain control target was set to 3e6, with a maximum injection time of 10 ms. Dynamic exclusion was applied for a duration of 40.0 s. Survey scans were acquired at a resolution of 70,000 at m/z 200, while the resolution for HCD spectra was set to 17,500 at m/z 200, with an isolation width of 2 m/z. The normalized collision energy was 30 eV, and the underfill ratio was defined as 0.1%. Peptide recognition mode was enabled during instrument operation.

#### 2.5.3 Protein identification and statistical analysis of differential results

The MS raw data for each sample was processed for identification and quantitative analysis using the MASCOT engine v.2.2 (Matrix Science, London, UK), embedded within Proteome Discoverer v.1.4. For the screening of significantly differential proteins, proteins with a Fold Change (FC) > 1.2 and *P* < 0.05 were defined as differentially expressed proteins (DEPs).

#### 2.5.4 Conduct clustering analysis and domain annotation

Hierarchical clustering analysis was performed utilizing Cluster 3.0 (http://bonsai.hgc.jp/~mdehoon/software/cluster/software.htm) in tandem with Java Treeview (http://jtreeview.sourceforge.net). During the hierarchical clustering process, the Euclidean distance algorithm was selected as the similarity metric, along with the average linkage clustering method. Domains were annotated by leveraging InterProScan to search protein sequences, thereby identifying protein domain entries from the Pfam database within the InterPro member databases.

#### 2.5.5 Functional annotation of differentially expressed proteins

To explore the potential roles of DEPs across different treatment groups, we conducted GO and KEGG pathway analyses on these DEPs. We utilized NCBI BLAST+ client software and InterProScan for local searches to identify homologous sequences, followed by functional annotation using Blast2 GO. For KEGG functional annotation, we mapped DEPs to the KEGG gene database using the KEGG Automatic Annotation Server v.2.0 (http://www.genome.jp/kaas-bin/kaas_main), subsequently categorizing the KEGG - mapped proteins and annotating the target protein set with KEGG pathways. After applying the Fisher’s exact test, we compared the distribution of GO categories and KEGG pathways between the target protein set and the overall protein set. Multiple test corrections were then performed using the Benjamini - Hochberg method to adjust the resulting *P* - values. Functional categories and pathways with *P* < 0.05 were considered significantly enriched. PPI analysis was based on interaction data from the STRING (http://string-db.org/) or IntAct (http://www.ebi.ac.uk/intact/main.xhtml) databases. Using Cytoscape, we constructed and analyzed PPI networks for the DEPs from different treatment groups.

### 2.6 Integrated analysis of transcriptomics and proteomics

The names of various proteins were converted into gene identifiers using NCBI (https://www.ncbi.nlm.nih.gov/). Subsequently, we integrated DEPs and DEGs for Pearson correlation analysis, utilizing ggplot2 in R v.4.3.3 to graphically display the correlation between transcript abundance and protein levels. The correlation coefficient was calculated using the Pearson method, and regression lines were established for correlation analysis using the linear model function in R. Statistical data were summarized using R. We then screened for corresponding DEPs and their matched transcripts for functional analysis. The transcriptome data, Trinity assembly sequences, and annotation table have been uploaded to the NCBI Sequence Read Archive (SRA) database with the accession number PRJNA1189655. The mass spectrometry proteomics data have been deposited in the ProteomeXchange Consortium via the PRIDE partner repository (26), with the dataset identifier PXD058399.

## 3 Results

### 3.1 Eukaryotic *de novo* transcriptome analysis results

#### 3.1.1 Transcriptome sequencing yield and assembly quality analysis

**Figure 1:**
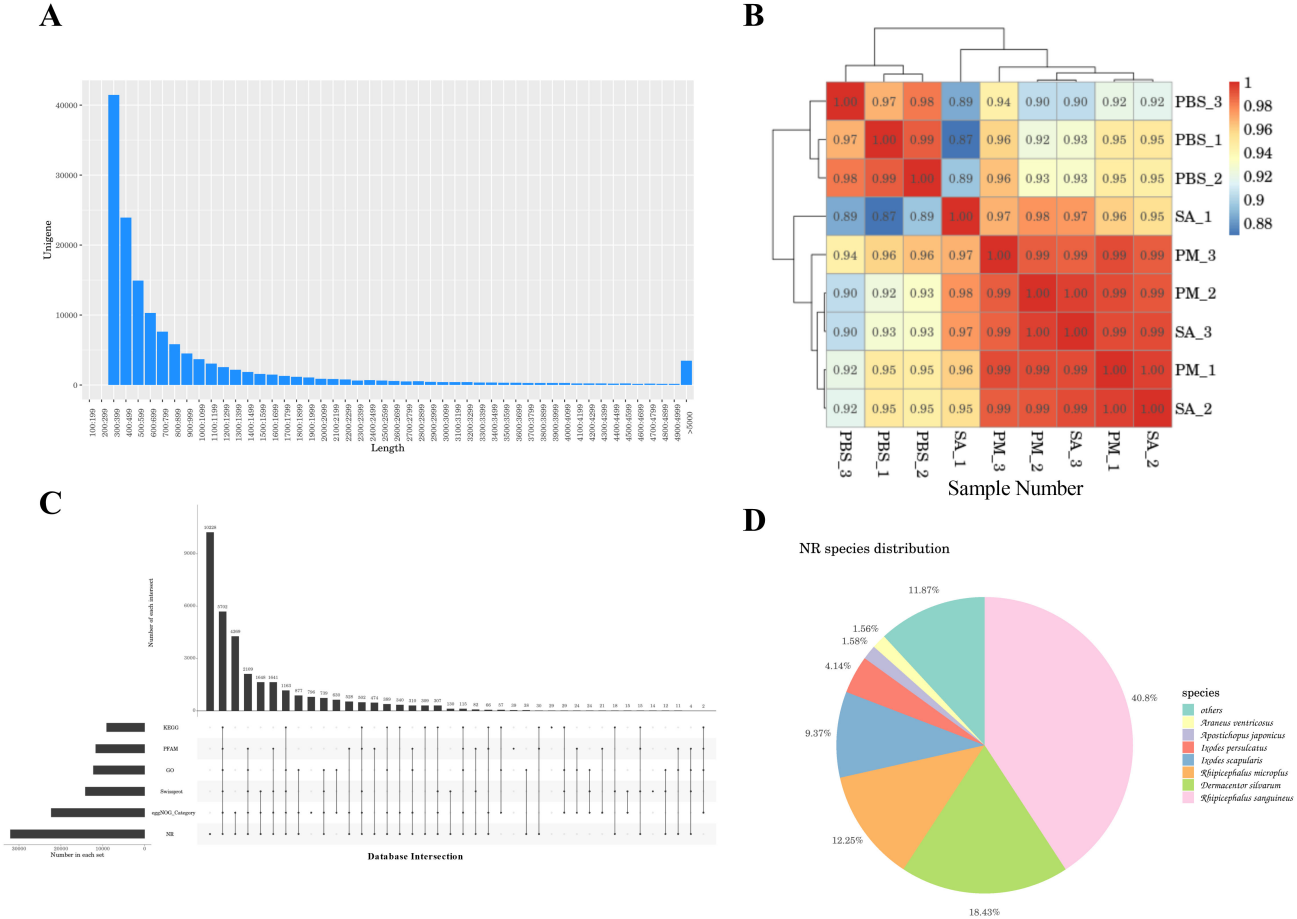
A comprehensive analysis of the transcriptome data. **(A)** Length distribution chart of unigenes. X-axis: Sequence length bins (bp); Y-axis: Frequency of unigenes. The majority of unigenes fall within 300-1,300 bp. **(B)** Sample correlation analysis. This analysis shows the correlation of gene expression profiles among different samples, with higher correlation coefficients between biological replicates. **(C)** upset plot of unigene annotation statistics. The x-axis represents the number of databases in which unigenes were annotated, and the y-axis represents the number of unigenes annotated in specific combinations of databases. This plot provides an overview of the annotation results of unigenes across different databases. **(D)** Similar species of the optimal hit in the Nr database. Pie chart showing taxonomic affiliation of best BLAST matches. Dominant species: Rhipicephalus sanguineus (40.8%), Dermacentor silvarum (18.4%), and Rhipicephalus micoplus (12.25%).

The treatment groups comprised *H. anatolicum* with *S. aureus* strains (SA-1, SA-2, SA-3), a *P. mirabilis* treatment group (PM-1, PM-2, PM-3), and a PBS control group (PBS-1, PBS-2, PBS-3), totaling nine samples. Transcriptome sequencing yielded 473,205,352 raw reads (**Table 1**). After filtering out low - quality data, 465,064,638 clean reads remained, representing 98.05% of the original reads. Using Trinity for *de novo* transcriptome assembly, we obtained 271,837 transcripts with an average length of 1,407.02 bp, an N50 length of 2,705 bp, and a GC content of 46.73%. Additionally, we identified 144,465 unigenes, averaging 1,014.15 bp in length, with an N50 length of 1,593 bp and a GC content of 45.81%. The assembly exhibited high integrity (**Table 2**). All unigene sequences exceeded 300 bp, with the longest spanning 35,521 bp. Most unigenes ranged between 300 and 1,300 bp in length (**Figure 1A**). Correlation analysis of the assembly data across samples revealed correlation coefficients exceeding 0.8, indicating a strong inter-sample correlation (**Figure 1B**).

**Table 1:** Post-sequencing data statistics for treatment samples.

Sample	Reads no.	Bases (bp)	Q30 (bp)	N (%)	Q20 (%)	Q30 (%)
SA-1	51,240,086	7,737,252,986	7,474,534,274	0.003361	98.81	96.60
SA-2	52,259,118	7,891,126,818	7,618,298,652	0.003389	98.77	96.54
SA-3	52,443,200	7,918,923,200	7,631,447,849	0.003369	98.69	96.37
PBS-1	48,937,026	7,389,490,926	7,118,159,768	0.003426	98.66	96.33
PBS-2	59,089,604	8,922,530,204	8,607,552,926	0.003353	98.71	96.47
PBS-3	56,414,350	8,518,566,850	8,237,700,397	0.003386	98.83	96.70
PM-1	56,716,430	8,564,180,930	8,249,119,720	0.003388	98.67	96.32
PM-2	47,592,354	7,186,445,454	6,932,559,356	0.003349	98.73	96.47
PM-3	48,513,184	7,325,490,784	7,068,398,575	0.003445	98.75	96.49

**Table 2:** Overall sequence statistics for transcripts and unigenes.

Description	Transcript	Unigene
Total Length (bp)	382,479,590	146,509,049
Sequence Number	271,837	144,465
Max. Length (bp)	35,521	35,521
Mean Length (bp)	1,407.02	1,014.15
N50 (bp)	2,705	1,593
N50 Sequence No.	38,463	20,973
N90 (bp)	504	403
N90 Sequence No.	172,703	102,138
GC%	46.73	45.81

#### 3.1.2 Functional annotation of unigenes

After assembling the transcripts, to achieve a comprehensive understanding of gene function, we aligned the 144,465 unigene sequences against six databases and identified the sequences with the highest alignment similarity. Among the six databases, the NR database exhibited the highest gene annotation rate, accounting for 22.19% of the total unigene sequences, whereas the KEGG database had a relatively lower annotation rate of 6.28%. A total of 3.95% of the genes were annotated in all databases (**Supplementary Table S2**). The unigene sequence annotation statistics are depicted in **Figure 1C**. Additionally, 33,743 unigene sequences (23.36%) did not match any known functional gene sequences and were classified as unknown genes, indicating that the sequencing might have captured numerous novel genes and non-coding RNA sequences (27, 28). By performing comparative annotation with the NR database, we gained insights into the similarity between the gene sequences of this species and those of closely related species, as well as the functional information of the genes. The analysis of E - values revealed that 10.5% of the annotated genes exhibited high homology, while 7.6% of the annotated genes had E - values of 0, suggesting no homology (**Supplementary Figure S1**). Furthermore, 60.44% of the annotated genes shared more than 60% similarity with known proteins (**Supplementary Figure S2**). In terms of species comparison, *Rhipicephalus sanguineus* and *Dermacentor silvarum* exhibited relatively high comparison repetition rates of 40.8% and 18.43%, respectively (**Figure 1D**).

According to the GO functional annotation presented in **Supplementary Figure S3**, most of the unigenes were involved in cellular component (cells, cell part, organelles), biological process (cellular processes), and molecular function (binding). To further explore the biological functions and potential pathways of these unigenes, a total of 144,465 unigenes were mapped into the KEGG database. The results indicated that 11,080 sequences were annotated as related to metabolic pathways. Among these, the environmental information processing branch, a key functional category in the KEGG database, covers molecular mechanisms of how an organism senses, transmits, and responds to external environmental signals. We found that the signal transduction pathway in this category had the most annotations, with 1,244 (11.26%). Second was the transport and catabolism pathway in the cellular processes branch, with 926 (8.35%). These two pathways could potentially have a close association with the tick’s adaptation to its external environment, as well as its physiological processes involving substance transport and catabolism. In addition, 613 sequences were annotated within the immune system sub - branch of the organic systems branch, potentially indicating the immune defense responses mounted by ticks following bacterial infection (**Supplementary Figure S4**). We further classified the transcription factors into 56 categories, among which 48 unigenes were annotated as the Homeobox type and 38 as the zf - C2H2 type (**Supplementary Figure S5**).

#### 3.1.3 Differentially expressed genes and functional enrichment analysis

**Figure 2:**
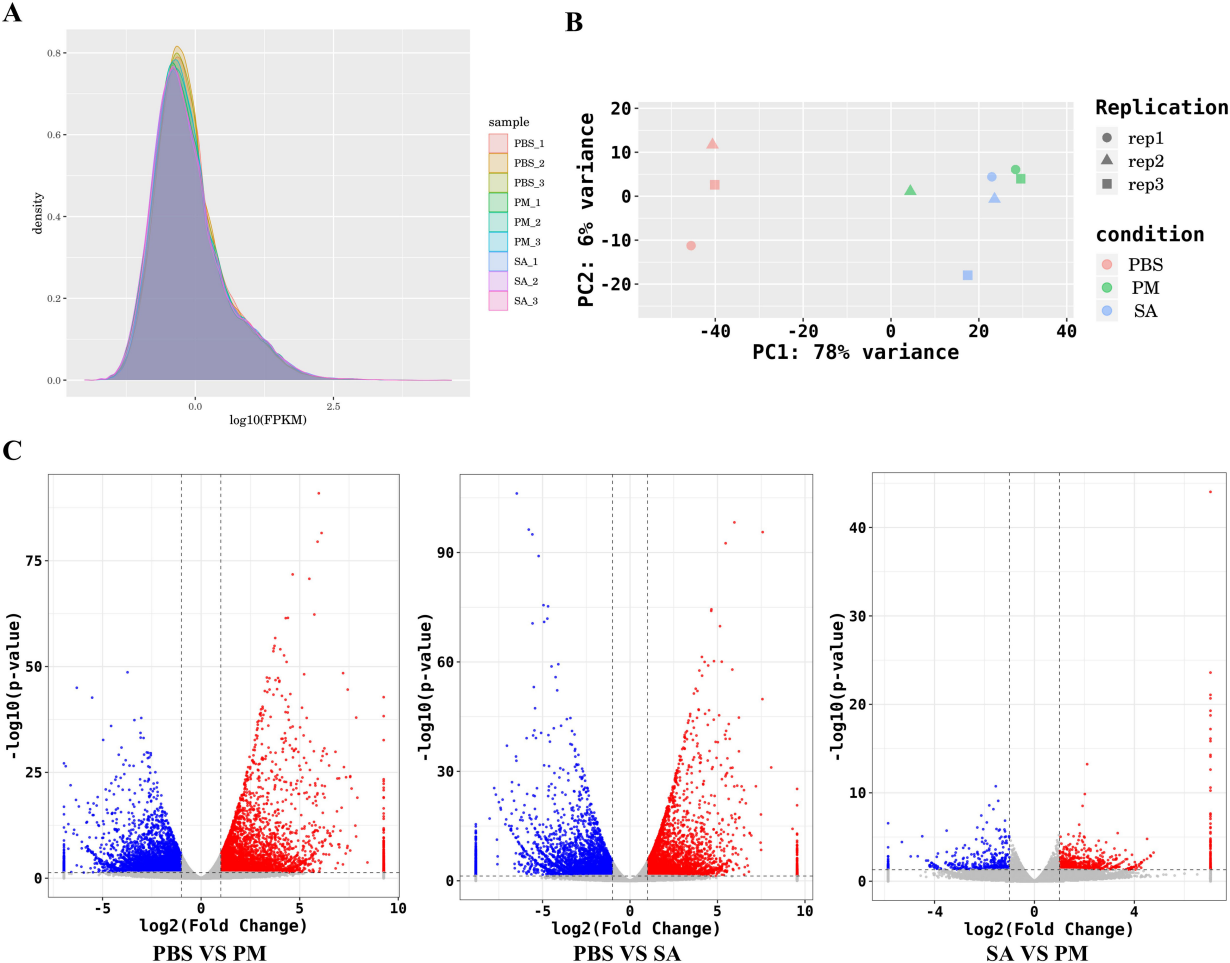
Global quantification analysis and DEGs identification. **(A)** FPKM density distribution comparing the expression levels of PBS-treated, *E. coli*-treated and *S. aureus*-treated samples, illustrating the distribution of gene expression values. The peaks and spreads of the density curves indicate the overall expression patterns and variations in each group. **(B)** A principal component analysis (PCA) plot illustrates the differences among various cDNA libraries. Each point on the plot represents a sample, with different colors indicating different treatment groups. **(C)** A volcano plot of differentially expressed genes (DEGs) shows gene distribution based on expression fold - change and statistical significance (*p* < 0.05, |log2FC|>1.0).

The comparison of gene expression profiles was displayed by the FPKM density values (**Figure 2A**). As shown in **Figure 2B**, there were significant differences between the samples from the *S. aureus* and *P. mirabilis* treatment groups and the PBS control group, indicating pronounced inter - group variations. Through the statistical analysis of the number of differentially expressed genes in the three comparison groups of PM/PBS, SA/PBS, and SA/PM, a total of 21,315 DEGs were identified. Among them, the expression profiles revealed 5,240 genes to be up-regulated and 4,990 genes to be down - regulated when comparing PM to PBS. Similarly, between SA and PBS, there were 4,493 genes up - regulated and 5,283 genes down - regulated. In contrast, the comparison between PM and SA exhibited a relatively smaller number of DEGs, with 830 genes up - regulated and 479 genes down - regulated (**Figure 2C**).

The GO enrichment analysis of DEGs indicated that all categories were enriched. Notably, the top five most significantly enriched GO terms when comparing the PM and SA groups to the PBS group were: intrinsic component of membrane, integral component of membrane, extracellular region, monooxygenase activity, and extracellular space. Furthermore, several GO terms pertinent to metabolic processes were also prominently enriched, including lipid metabolic process, oxoacid metabolic process, and chitin metabolic process, among others. (**Supplementary Figure S6**).

KEGG enrichment analyses were conducted on all the up - regulated and down - regulated differentially expressed genes (DEGs). The results revealed that, in the comparison between the PM and PBS groups, the up - regulated genes were significantly enriched in pathways like the Toll and IMD signaling pathways, MAPK signaling pathway, and apoptosis. In contrast, the down - regulated genes were mainly enriched in the retinol metabolism and glycerophospholipid metabolism pathways. For the comparison between the SA and PBS groups, the up-regulated DEGs were prominently enriched in pathways such as ribosome biogenesis in eukaryotes, aminoacyl - tRNA biosynthesis, and apoptosis. On the other hand, the majority of the down - regulated DEGs in this comparison were enriched in lysosome, fatty acid elongation, and steroid biosynthesis (**Supplementary Figure S7**). An analysis was focused on the enrichment of KEGG pathways related to immunity, revealing that the Toll and IMD signaling pathways, MAPK signaling pathway, and NF - κB signaling pathway were significantly enriched. Within the Toll and IMD signaling pathways, 10 up - regulated genes and 4 down - regulated genes were identified as influencing the defense response and apoptosis. The MAPK signaling pathway was impacted by 33 DEGs, among which were G protein - coupled receptors (GPCRs), E3 ubiquitin protein ligase (Pdzrn3), myeloid differentiation primary response protein MyD88 - like, specificity protein phosphatase 8 - like, and FGF - 9 (fibroblast growth factor - 9; with 27 up - regulated and 6 down - regulated). Additionally, a total of 14 DEGs (8 up - regulated and 6 down - regulated) were detected in the NF - κB signaling pathway (**Supplementary Table S3**). Previous studies have demonstrated that these three pathways regulate innate immune responses in ticks. Specifically, research on *Amblyomma americanum* and *Hyalomma asiaticum* following *Escherichia coli* treatment revealed activation of multiple immune - related genes, as well as the Toll and IMD signaling pathways, MAPK signaling pathway, and NF - κB signaling pathway (21, 33). This indicates conserved functions of these pathways in defending against Gram-negative bacterial invasion and highlights their critical roles in the innate immune defense system of *Hyalomma anatolicum*.

Based on the experimental data, it was observed that bacterial invasion had a significant impact on the gene functional profile of *H. anatolicum*. A detailed analysis was conducted in this study, examining the changes in expression of factors related to the immune response of *H. anatolicum* under the stress of bacterial invasion. Special attention was given to key immune molecules, such as antimicrobial peptides (comprising defensins and Microplusin), lysozymes, and lectins. In comparison to the PBS control group, a total of 16 antimicrobial peptide family members were identified in both the PM group and the SA group, encompassing 9 defensins and 7 microplusin, with 6 of them showing differential up-regulation in expression. Furthermore, 6 lysozyme family members (2 up - regulated) and 12 lectin family members (7 up - regulated) were also detected (**Supplementary Table S4**). These findings uncovered the immunomodulatory mechanisms employed by *H. anatolicum* in response to bacterial challenges.

#### 3.1.4 PPI network analysis and RT-qPCR validation

**Figure 3:**
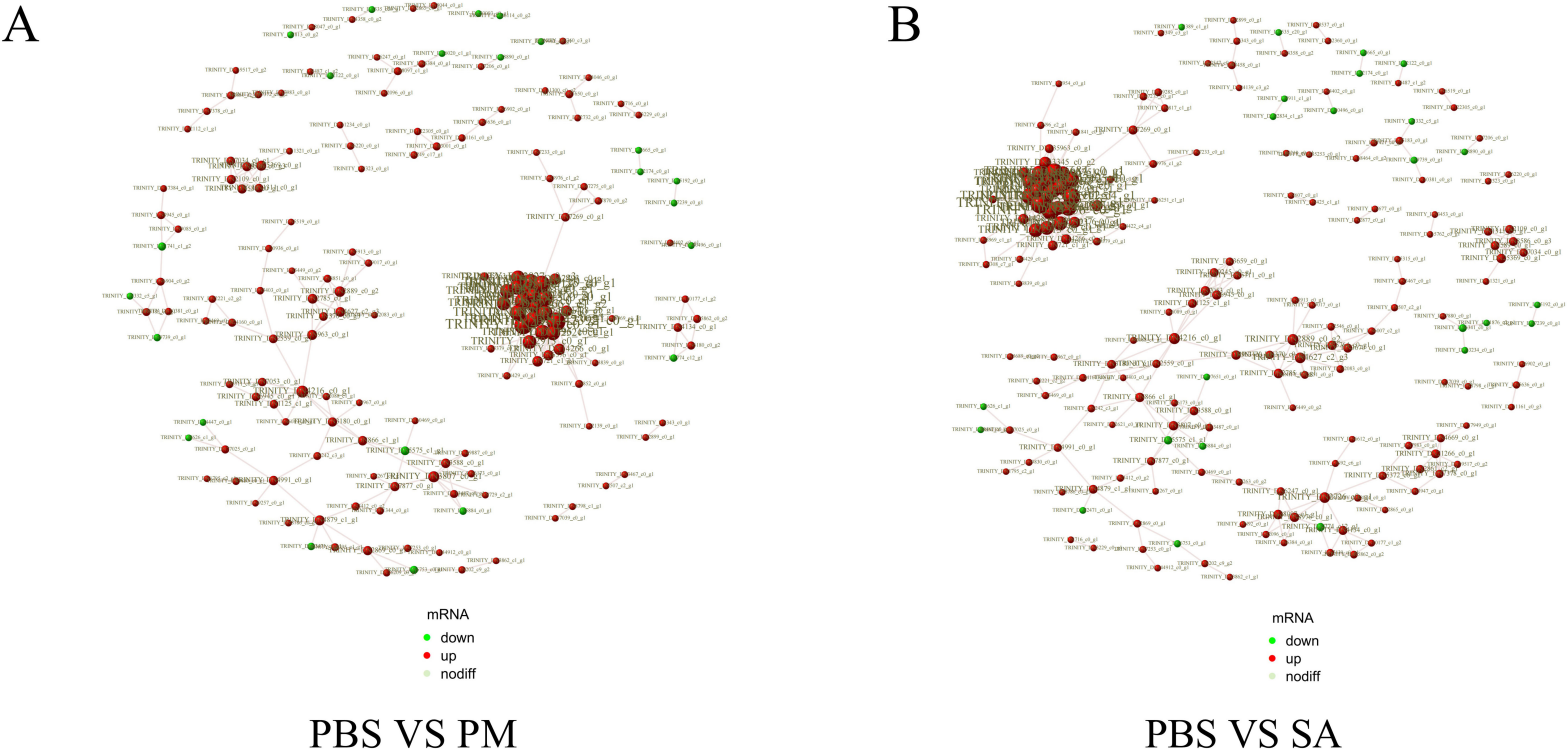
Protein-protein interaction (PPI) networks of differentially expressed genes. Edges represent interactions, and node size indicates degree. The node color (red for up-regulated, green for down-regulated genes) shows the significance of gene expression changes. **(A)** PPI network for the PBS/PM group. **(B)** PPI network for the PBS/SA group.

The PPI network for the DEGs in the PBS/SA group comprised 413 interactions among 194 nodes, whereas the PPI network for the DEGs in the PBS/PM group consisted of 329 interactions involving 168 nodes (**Figure 3**). In the study, significant interactions were identified among genes in the SA/PBS comparison group, which included the ribosome biogenesis protein WDR12, the bystin protein, and heat shock proteins (HSPs). Similarly, interactions were detected in the PM/PBS comparison group, involving the nucleolar complex protein 4, the U3 small nucleolar RNA - associated protein, and the bifunctional glutamate/proline - tRNA ligase (**Supplementary Table S5**). Further analysis through GO and KEGG enrichment revealed that these proteins were notably enriched in the IL - 17 signaling pathway, the MAPK signaling pathway, and the aminoacyl - tRNA biosynthesis pathway (**Supplementary Figure S7**). Based on these observations, it is speculated that the IL - 17 signaling pathway, the MAPK signaling pathway, and the aminoacyl - tRNA biosynthesis pathway are potentially involved in regulating the immune defense mechanisms of *H. anatolicum* at the transcriptional level.

**Figure 4:**
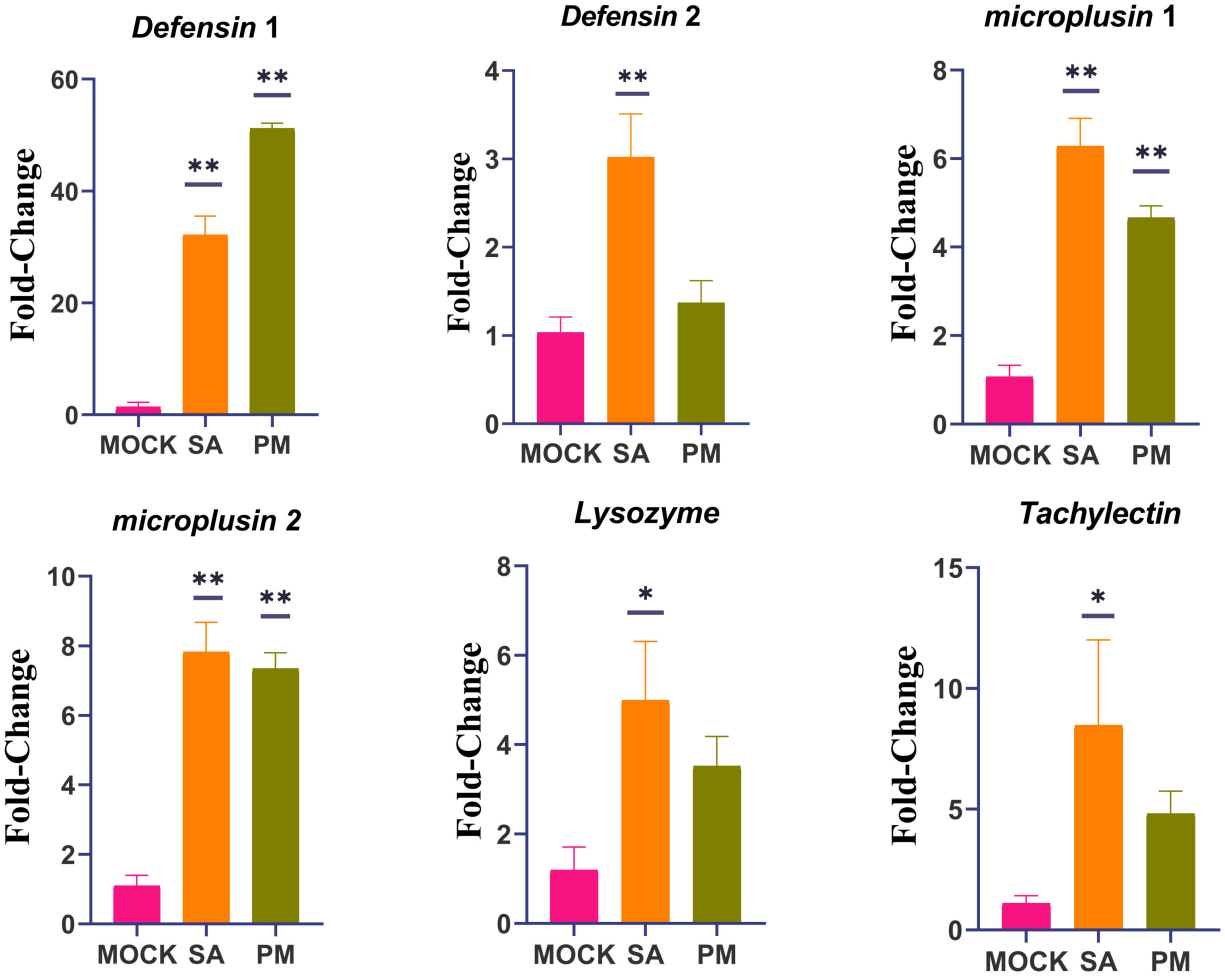
RT-qPCR validation of differentially expressed genes identified by RNA-seq. *H. anatolicum* translation elongation factor EF-1 alpha (ELF1α) was used as an internal control. Data are presented as mean ± SEM. *p < 0.05, **p < 0.01. The experiments were repeated three times. Data were normalized to the expression level of ticks from mock-infected group. Mock, PBS-injected females after 24 h; SA, *S. Aureus*-injected females after 24 h; PM, *P. mirabilis*-injected females after 24 h.

Six immune - related genes were selected for RT - qPCR relative quantitative analysis, with the aim of further confirming the protein - coding genes identified in the transcriptome. The results obtained from this analysis demonstrated the reliability of the RNA - seq data, as the expression profiles of the selected genes were found to be consistent with the fold change values calculated during the transcriptome analysis (**Figure 4**).

### 3.2 Results of TMT-labeled quantitative proteomics analysis

#### 3.2.1 Protein identification and DEPs screening

**Figure 5:**
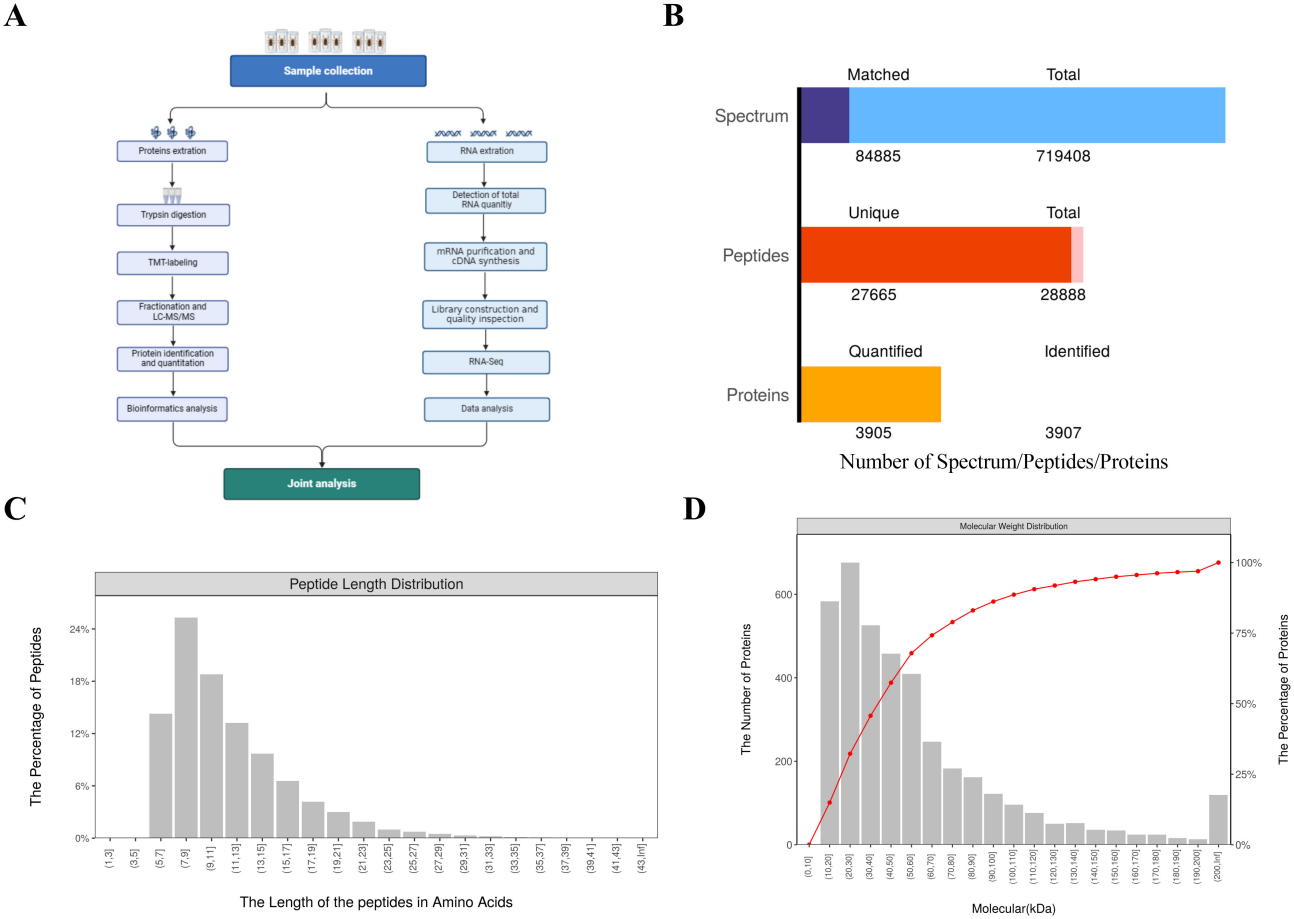
Overview of the combined transcriptome and proteome analysis data. **(A)** Flowchart of the analysis process. **(B)** bar chart summarizing identification and quantification results. **(C)** distribution chart of peptide sequence lengths. **(D)** Relative Molecular Mass Distribution of Identified Proteins.

To investigate the defense mechanisms and protein dynamics in *H. anatolicum* following bacterial challenge, we conducted TMT-based quantitative proteomic analysis across three comparative groups. Proteomic and transcriptomic profiles were established for *H. anatolicum* specimens treated with Staphylococcus aureus, Proteus mirabilis, or phosphate-buffered saline (**Figure 5A**).

A comprehensive analysis revealed the identification of 28,888 peptides, including 27,665 unique peptides. Across the three treatment groups, a total of 3,907 proteins were detected, with 3,905 of these being quantifiable (**Figure 5B**). The length distribution of the peptides indicated that the majority ranged between 7 and 15 amino acids (**Figure 5C**). Additionally, the number of identified peptides exhibited a decreasing trend as molecular weight increased from 30 to 200 kDa (**Figure 5D**).

**Figure 6:**
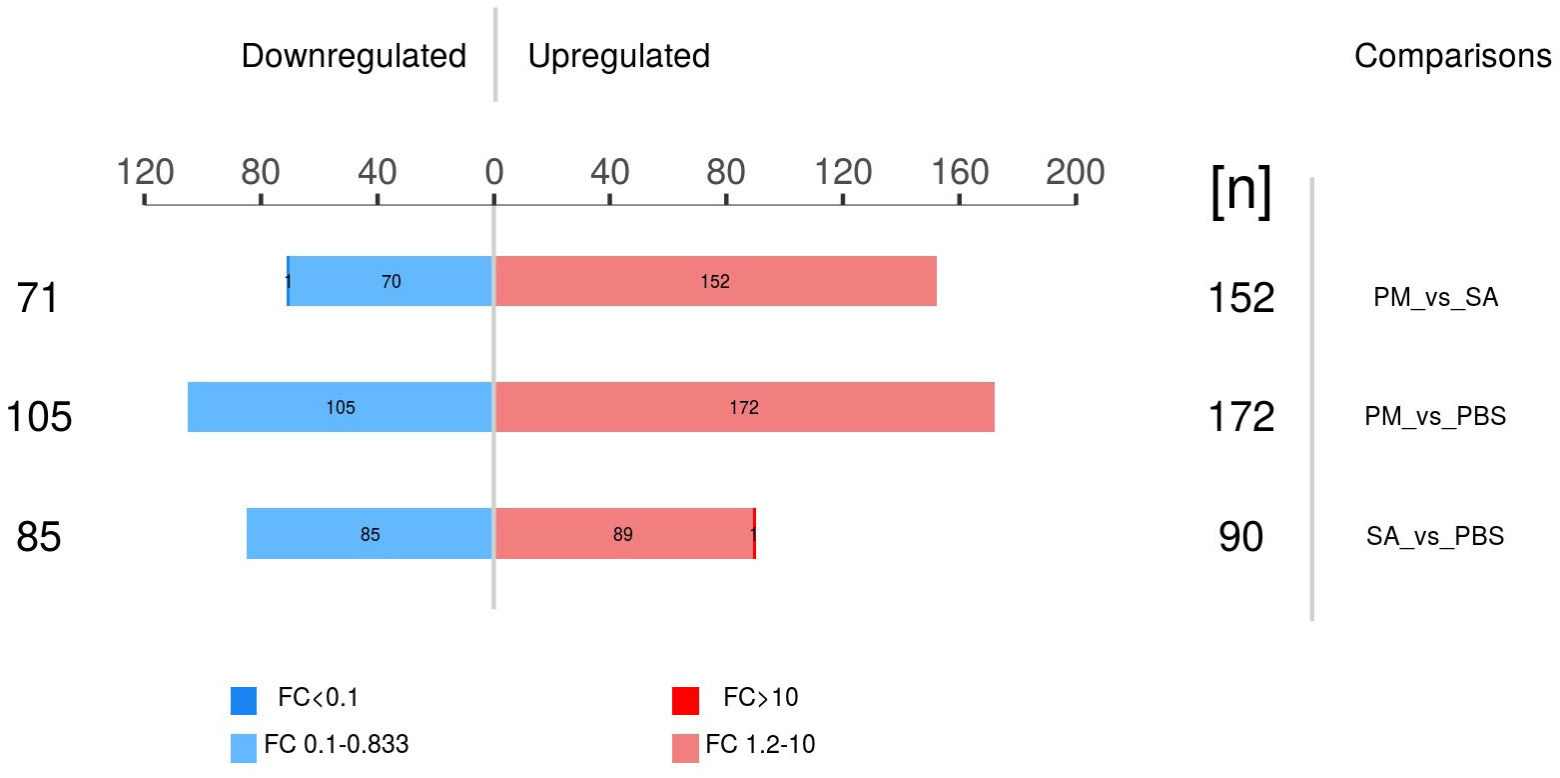
Differential expressed proteins in three comparison groups (p < 0.05, |fold change| > 1.2 or < 0.83).

A thorough differential screening of the experimental data was conducted to determine the number of DEPs among the three comparison groups. With FC greater than 1.2 times and *P* < 0.05, the numbers of up - regulated and down - regulated DEPs were obtained for each of the three comparison groups (**Figure 6**). The PM/PBS comparison group showed the highest count of differentially expressed proteins, with a total of 277, whereas the SA/PBS group had 175 DEPs. Among these, 172 proteins in the PM group exhibited significantly higher expression levels compared to those in the PBS group, and 90 proteins were found to be differentially up - regulated in the SA group when compared to the PBS group.

#### 3.2.2 Functional enrichment analysis of differentially expressed proteins

Functional enrichment analysis of differentially expressed proteins (DEPs) was conducted GO, KEGG, and protein domain databases. In the PM/PBS comparison group, the top five enriched GO terms were cell surface receptor signaling pathway, Toll signaling pathway, enzyme activator activity, mitochondrial organization, and serine tRNA aminoacylation, with the cell surface receptor signaling pathway being particularly significantly enriched. For the SA/PBS comparison group, the top 5 terms were calcium ion binding, oxidoreductase activity, cell surface receptor signaling pathway, coenzyme binding, and cofactor binding, among which calcium ion binding stood out as significantly enriched. Additionally, as depicted in **Supplementary Figure S8**, the majority of the DEPs were categorized under the molecular function terms of catalytic activity and binding. This suggests that these DEPs may play crucial roles in the immune response of ticks.

**Figure 7:**
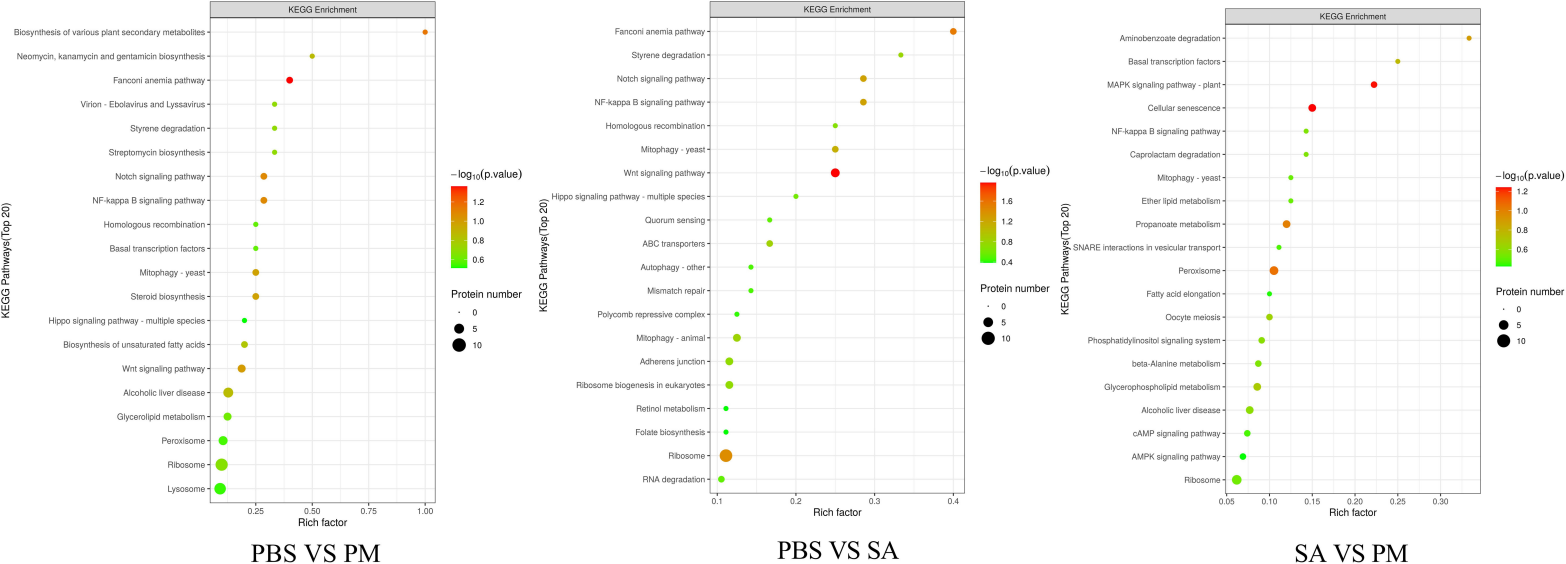
KEGG pathway enrichment bubble plot.

**Figure 8:**
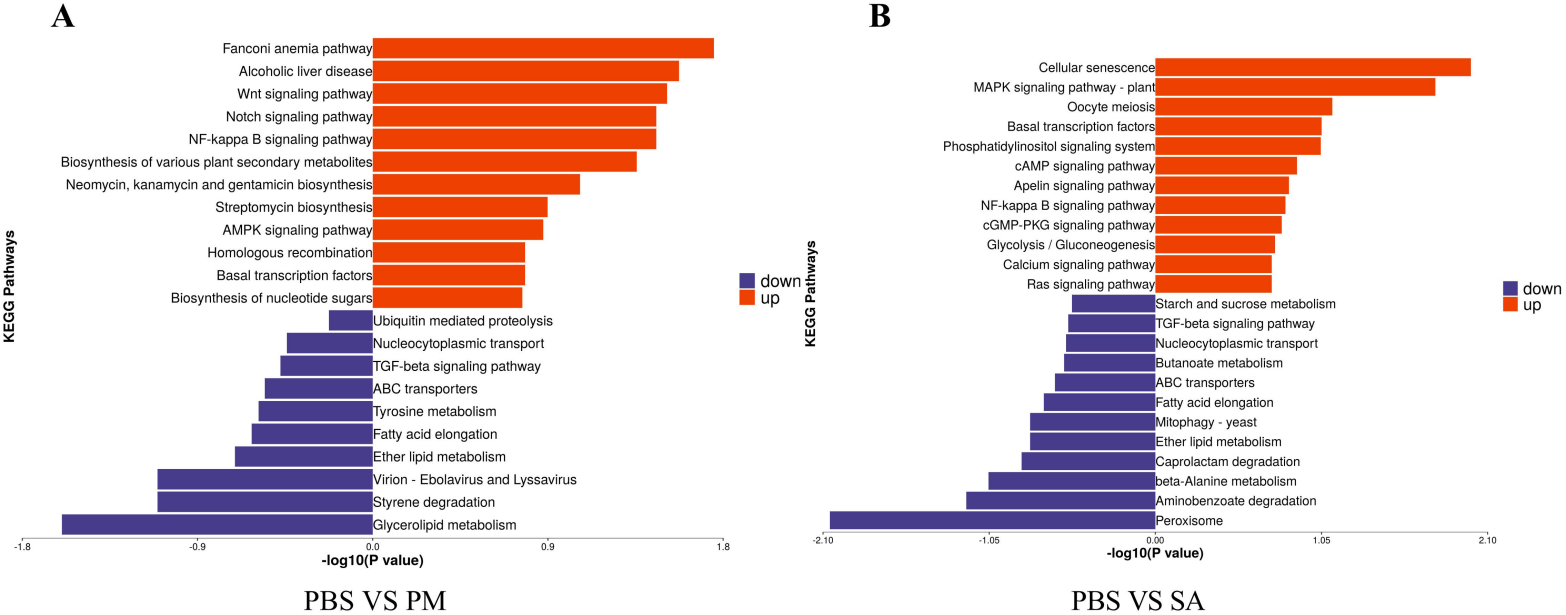
KEGG Enrichment of Differentially Expressed Proteins. **(A)** PBS VS PM treatment group. **(B)** PBS VS SA treatment group.

In the three groups, the DEPs were mainly enriched in the Fanconi anemia pathway, cellular senescence, and signal transduction pathways (**Figure 7**). For the PM/PBS group, the up - regulated DEPs were significantly enriched in the Fanconi anemia pathway, NF - kB signaling pathway, Notch signaling pathway, and Wnt signaling pathway, while the downregulated DEPs were mainly enriched in lysosomes and glycerolipid metabolism (**Figure 8A**). For the SA/PBS group, the upregulated DEPs were significantly enriched in cellular senescence and MAPK signaling pathway. Most of the down-regulated DEPs were enriched in peroxisomes and aminobenzoate degradation (**Figure 8B**). Domain enrichment analysis showed that the serine protease inhibitor and lipase domains were significantly enriched in the PM/PBS group, while the Astacin (a branch of serine peptidases) domain was significantly enriched in the SA/PBS group (**Supplementary Figure S9**). Meanwhile, it was observed that there was a notable enrichment of the C - type lectin domain in the SA/PBS group. These C - type lectin domains possess the ability to recognize and bind to specific sugar structures present on the surface of pathogens. Consequently, they play a part in the recognition and elimination of these pathogens.

Furthermore, the research focused on proteins and pathways associated with the innate immune defense mechanisms of ticks, encompassing AMPs such as defensin and microplusin, lectins, as well as signaling pathways including the NF - κB, Toll, and JAK/STAT pathways. Each gene family exhibited distinct expression trends. Intriguingly, defensin and microplusin were absent from the proteomic analysis conducted 24 hours post-puncture injection. Among the various signaling pathways like NF - κB, Toll, IMD, and JAK/STAT, only the NF - κB and JAK/STAT pathways were found to be enriched, while the Toll-like receptor signaling pathway and the IMD immune deficiency pathway were undetected. Additionally, notable differences in immune defense-related proteins were observed between the SA/PBS group and the PM/PBS group.

#### 3.2.3 Differential protein network interaction analysis

**Figure 9:**
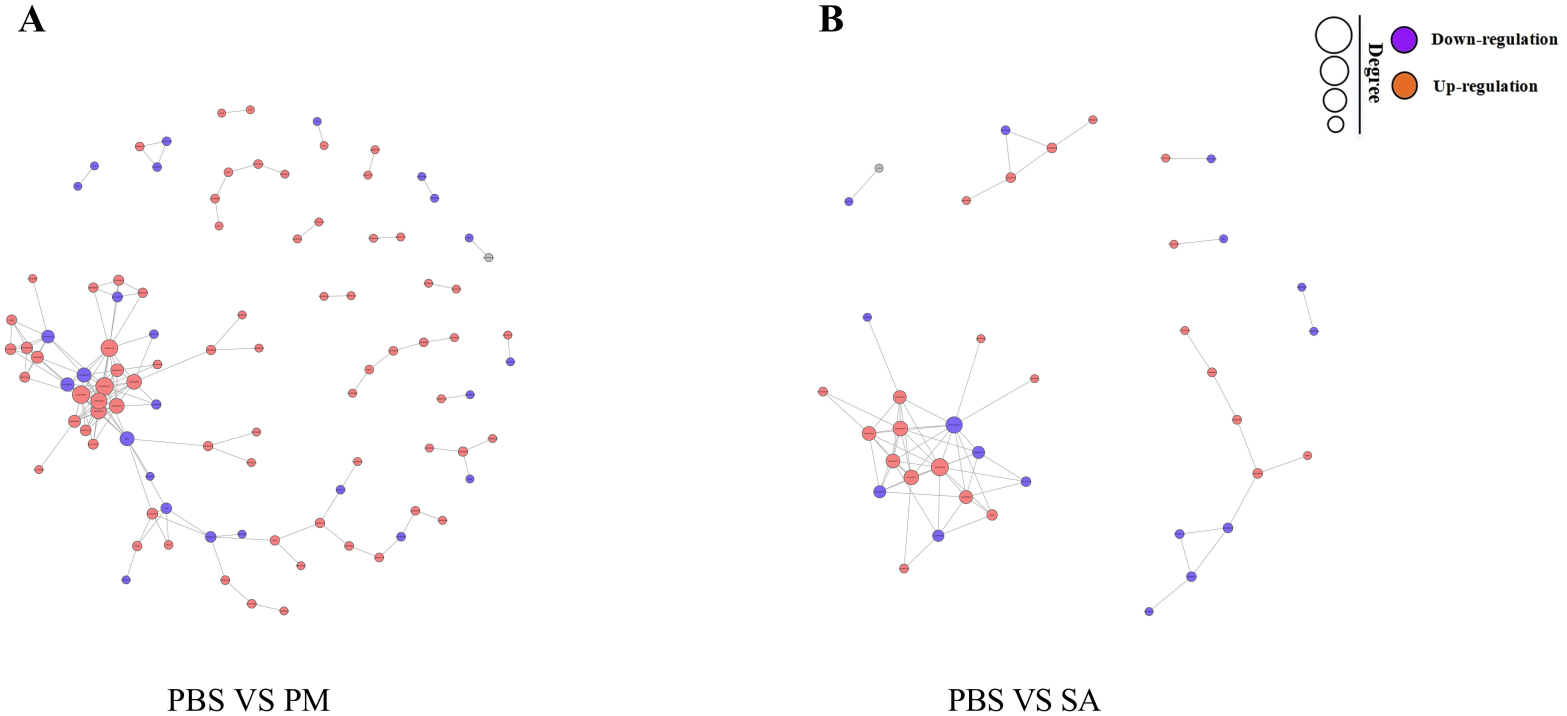
Differentially expressed protein-protein interaction (PPI) networ. Edges represent interactions, and node size indicates degree. The node color (orange for up-regulated, purple for down-regulated genes) shows the significance of gene expression changes. **(A)** PPI network for the PBS/PM group. **(B)** PPI network for the PBS/SA group.

The PPI network of DEPs in the PM/PBS group contained 155 edges and 97 nodes (**Figure 9A**). Among them, there were strong interactions among the ribosomal protein family, elongation factor 1-alpha (ELF1α), myosin (Myo9), and actin (Arp1). The PPI network in the SA/PBS group contained 40 nodes and 69 edges (**Figure 9B**). Strong interactions were found between the ribosomal protein family and the helicase family (Rm62). During the process of protein interaction analysis, we observed that a series of proteins were significantly concentrated in the NF - κB signaling pathway and the Wnt signaling pathway in the GO and KEGG pathway enrichment analyses. Moreover, the data analysis revealed that the expression levels of these proteins showed an upward trend with the invasion of bacteria. Based on these findings, we inferred that at the proteomic level, the NF - κB signaling pathway and the Wnt signaling pathway might be involved in regulating the innate immune defense response of ticks.

### 3.3 Results of integrated transcriptomics and proteomics analysis

**Figure 10:**
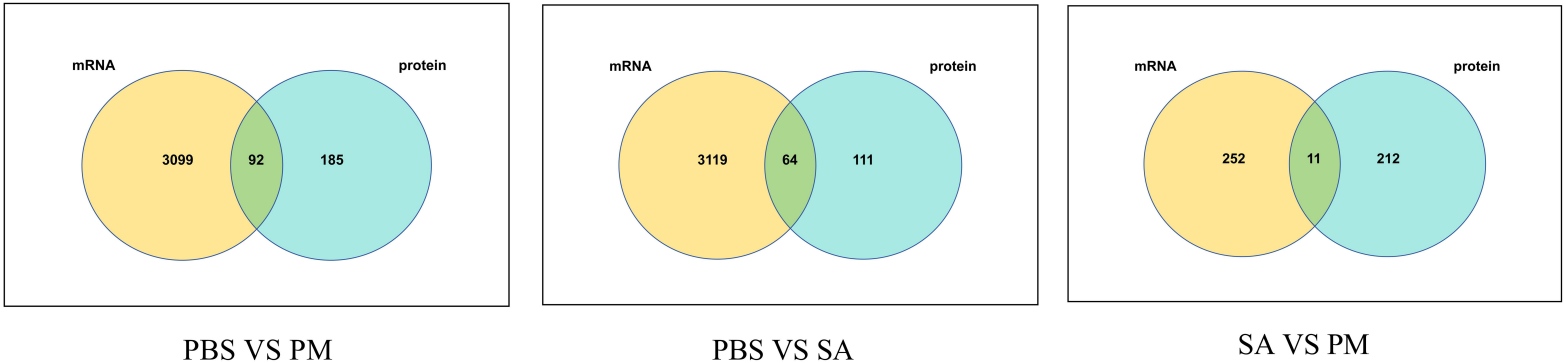
Venn diagram illustrating the corresponding relationships between differentially expressed proteins and their corresponding differentially expressed transcripts. Each circle represents a set of either DEPs or DEGs, with the overlapping regions indicating the number of genes that are differentially expressed at both the transcriptional and translational levels.

A total of 92, 64, and 11 DEPs and DEGs with corresponding relationships were identified (**Figure 10**). Furthermore, an analysis of the changing trends of these DEGs was conducted across three comparison groups at both the transcriptome and proteome levels. At the mRNA and protein expression levels, in the PM/PBS group, it was observed that 80 genes exhibited the same trend, with 42 being up - regulated and 38 being down - regulated. Additionally, four DEGs showed up - regulation at the transcriptome level but down - regulation at the proteome level, while eight DEGs demonstrated down - regulation at the transcriptome level but up - regulation at the proteome level. In the SA/PBS group, a total of 56 genes displayed the same trend at both the transcriptional and protein levels, with 28 being up-regulated and 28 being down - regulated. Among these, five genes were up - regulated transcriptionally but down-regulated at the protein level, and three genes were down - regulated transcriptionally but up - regulated at the protein level. Lastly, in the SA/PM group, 11 genes were found to have the same trend at both the transcriptional and protein levels, with 10 being up - regulated and 1 being down - regulated.

**Figure 11:**
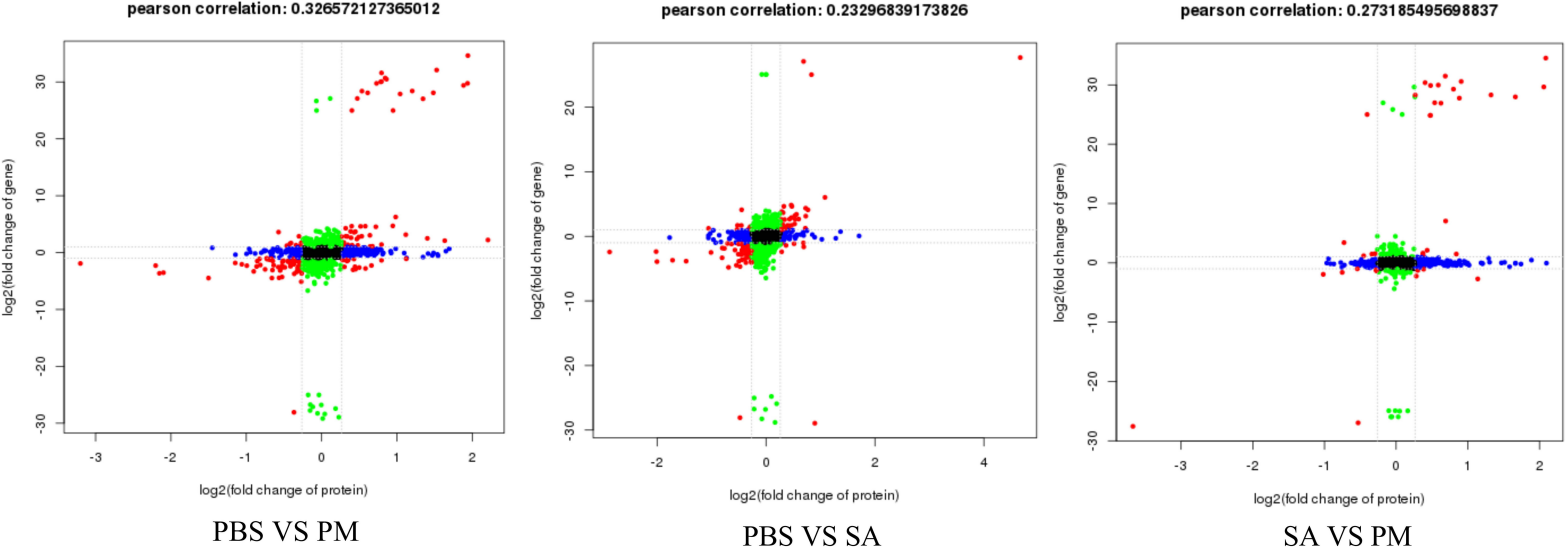
Correlation analysis of protein and gene expression levels. The x-axis and y-axis represent fold changes in protein and gene expression. Red points indicate results with significant differential expression at both transcript and protein levels; blue indicates significant protein-level differences only; green indicates significant transcript-level differences only; black represents no significant differences in either level.

Across three groups, more than approximately 26% of the genes and proteins exhibited differential expression patterns. Notably, several proteins situated in the third and seventh quadrants displayed expression patterns that were in harmony with their corresponding genes, indicating that these proteins undergo transcriptional regulation (**Figure 11**). Additionally, a comprehensive analysis revealed 53 DEGs that were up-regulated in both the transcriptome and proteome. These DEGs primarily focused on enhancing immune defense mechanisms, responding to stress, regulating metabolic processes, and maintaining cellular structure and functionality.

Among the identified DEGs, there were seven ribosome-related proteins (including four bacterial proteins), two proteins associated with glucose metabolism, one linked to lipid metabolism, two outer membrane proteins originating from bacteria, one MAPK - binding protein, one toxin protein, one nucleolar complex protein, one acetyl - CoA synthetase, and one transporter protein, among others (**Supplementary Table S6**). By comparing transcriptome and proteome data, researchers inferred that the overexpression of numerous ribosome proteins and their intense interaction reactions might contribute to protein stabilization. Furthermore, cells might bolster their stress response and immune defense by upregulating the expression of ribosome proteins (29).

GO and KEGG enrichment analyses further illuminated that these DEGs and DEPs were predominantly involved in Toll and IMD signaling pathways, the PI3K - Akt signaling pathway, and the amino sugar and nucleotide sugar metabolism signaling pathway, among others (**Supplementary Figure S7**).

Finally, the analysis revealed a total of 67 proteins related to the innate immune defense and pathways of ticks, as shown in **Supplementary Table S7**. Notably, as presented in **Supplementary Table S8**, several key components of the tick’s innate immune system, including AMPs, defensins, and lysozymes, were found to be overexpressed solely at the transcriptional level. This observation may suggest that the protein expression of these factors exhibits certain spatio - temporal specificity. However, the analysis also identified a pathogen - associated molecular pattern (PAMP) - recognizing lectin that was over - expressed at both the transcriptional and protein levels. This finding indicates that the lectin can rapidly respond to the threat posed by external pathogen invasion, further underscoring its significance in the immune defense mechanisms of this tick species.

## 4 Discussion

The innate immune responses in ticks are controlled by an intricate network of genes and signaling pathways. Previous research have illuminated significant transcriptional alterations in ticks on bacterial challenge (28, 30). The IMD, JAK/STAT, and Toll signaling pathways occupy vital roles in managing bacterial, viral, and parasitic infections within tick species. These immune signaling cascades empower ticks to mount effective defenses against a diverse array of microbial threats (11, 31, 32). Activation of the Toll pathway, which is stimulated by fungi and Gram - positive bacteria, is facilitated through an extracellular clip - domain serine protease (cSP) cascade. Conversely, the IMD pathway is engaged by Gram-negative bacteria. Such pathway activations lead to the up - regulation of antimicrobial peptide (AMP) transcription, mediated by the NF - κB transcription factor (33). The JAK/STAT signaling pathway, on the other hand, modulates the expression of AMP genes through STAT transcription factors and has been linked to immune responses directed against viral and parasitic infections (34, 35). Deciphering the intricacies of tick innate immunity presents potential benefits for the prevention of tick - borne infections and the safeguarding of public health.

The selection of these two bacterial strains for the experiment was based on their belonging to different bacterial classifications, facilitating the determination of whether *H. anatolicum* mounts distinct immune responses and produces antimicrobial substances against varying bacterial classes. Following 24 hours of treatment with *S. aureus* and *P. mirabilis*, gene expression analysis was conducted on *H. anatolicum*, revealing numerous DEGs and DEPs. By utilizing FPKM density values to study gene expression, distinct gene and protein expression profiles were identified between the SA and PM treated groups and the PBS control group, further indicating significant expression responses in *H. anatolicum* to bacterial challenge. In a study focusing on *Amblyomma americanum*, significant up - regulation of multiple immune - related genes, including those associated with the Toll, IMD, and JAK/STAT signaling pathways, was observed at 3, 6, 12, and 24 hours post - stimulation with *E. coli* (21). Similarly, in a related study where *Rhipicephalus microplus* was induced with *E. coli*, activation of the tick’s immune system was also observed (36). These findings reveal the existence of a series of conserved immune response pathways at both the cellular and whole - tick levels, playing crucial roles in the tick’s defense mechanisms against bacterial challenges.

In the current study, quantitative proteomics revealed a total of 3,907 proteins across all samples. Through differential analysis, 67 genes related to tick immune defense were detected and quantified in both transcriptome and proteome data. Among these, the differential expression changes of lectins following *S. aureus* and *P. mirabilis* treatments. This observation is in line with earlier studies that explored the role of lectins in immune defense against pathogenic and non - pathogenic bacterial infections (9, 15, 37). Given that ticks may respond to bacterial invasion by enhancing the production or release of lectins, the observed upregulation of lectin expression levels suggests a possible innate immune strategy employed by *H. anatolicum* to defend against bacterial entry.

Many transcription factors serve as key regulators of cell differentiation, environmental stress responses, and immune responses. Some transcription factors, such as zf - LITAF - like, zf - C2H2, and ZBTB, are competent in modulating the innate immune mechanisms of epithelial cells and the host upon pathogen invasion (38, 39). Among the major transcription factor subfamilies, the Homeobox, zf - C2H2, bHLH, and HMG families exhibit the highest number of differentially expressed transcription factors, indicating their significant roles in regulating the observed changes in gene expression. Various subfamilies of transcription factors have been identified as participating in the regulation of a range of biological processes and possess the ability to modulate the expression of multiple genes. These transcription factor subfamilies may play crucial roles in integrating and coordinating the observed gene expression patterns and their functional transitions (21).

By analyzing the innate immune response of *H. anatolicum* ticks to bacterial stimulation, the defensive mechanisms of ticks against bacterial pathogens have been unveiled. Specifically, genes associated with AMPs, such as defensins and microplusins, exhibited significant up - regulation in the ticks’ immune response. These small molecular AMPs, along with lysozymes and lectins, constitute the basis of the innate immune barrier in organisms and play crucial roles in the immune defense of all arthropods (40–45). These peptide molecules demonstrate remarkable abilities to target and kill bacterial and other microbial pathogens. The enhanced expression of AMP - related genes suggests that *H. anatolicum* initiates an effective antimicrobial response mechanism upon infection with *S. aureus* and *P. mirabilis*, a process that may be crucial for clearing the pathogens that cause infection. Previous studies have shown a significant increase in defensin expression levels in the transcriptome analysis of *H. anatolicum* upon bacterial challenge (33), as well as significant changes in the expression levels of immune response-related genes in *A. americanum* after *E. coli* treatment (21). In this study, 24 genes encoding AMPs, including 9 defensins, 7 microplusins, and 6 lysozymes, were identified and up - regulated upon bacterial challenge, potentially marking a defensive mechanism successful in reducing pathogen invasion. Additionally, 19 lectin genes were identified. Lectins, as a class of proteins competent in binding to polysaccharides, can bind to carbohydrates on the surface of pathogens, leading to damage to the cell wall and preventing microorganisms from attaching to host cells, thereby inhibiting pathogen growth and reproduction (37).

Through comprehensive transcriptome and proteome analyses, it was found that multiple KEGG pathways in *H. anatolicum* ticks were significantly up - regulated within 24 hours of treatment with *S. aureus* and *P. mirabilis*, including those involved in cellular senescence, the MAPK signaling pathway, and the NF - κB signaling pathway. The up - regulation of these pathways indicates the activation of defensive mechanisms against invasive pathogens. Additionally, various genes and proteins related to important signaling pathways such as NF - κB, JAK/STAT, Toll and IMD exhibited differential expression. These signaling pathways are well - known for their crucial roles in controlling immune responses and coordinating immune - related gene expression (9, 11). Interestingly, these immune - related KEGG pathways displayed different changes in the two omics analyses, with the Toll and IMD signaling pathways being significantly enriched in the transcriptome but not in the proteome, while the NF - κB signaling pathway was significantly enriched in both transcriptome and proteome analyses. This suggests a consistency of the NF - κB signaling pathway at both the transcriptional and translational levels and its potential importance in regulating immune responses. Furthermore, a large number of kinases were identified in both the transcriptome and proteome, playing pivotal roles in signal transduction pathways that regulate various cellular processes, including apoptosis, signal transduction, and inflammatory responses. The activity of kinases is tightly regulated, and changes in their activity may affect the host’s ability to respond to bacterial invasion and regulate immune signaling pathways, which are crucial for maintaining tissue homeostasis, eliminating diseased or infected cells, and controlling immune responses (46, 47).

The transcriptional and proteomic changes identified in this study align with and extend the findings from previous studies on tick immune responses. Similar to our results, earlier research has documented the activation of key immune signaling pathways, such as the Toll, IMD, and MAPK pathways, upon bacterial challenge in ticks. For instance, in a study on *Amblyomma americanum*, significant up - regulation of immune - related genes associated with these pathways was observed following *E. coli* stimulation, highlighting the conserved nature of these immune responses across tick species (21). The up - regulation of antimicrobial peptide (AMP) genes, including defensins and microplusins, in our study echoes the findings of other investigations where AMPs were found to play a central role in the tick’s defense against bacterial infections. These small molecular AMPs, along with lysozymes and lectins, form the basis of the innate immune barrier in arthropods and are crucial for their immune defense. The elevated expression of these genes in our study suggests that *H. anatolicum* mounts an effective antimicrobial response to *S. aureus* and *P. mirabilis* infections. While our results confirm the involvement of these well - characterized pathways, they also provide new insights into the complexity of the tick immune response. The integration of transcriptomic and proteomic data in our study offers a more comprehensive view of the immune mechanisms at play. For example, the NF - κB signaling pathway was found to be significantly enriched at both the transcriptional and translational levels in our analysis. This consistency underscores its potential importance in regulating immune responses in *H. anatolicum* and adds to the understanding of its role in tick immunity.

In this study, the observed changes in AMPs, immune - related signaling pathways, and the MAPK signaling pathway likely play pivotal roles in the immune defense of *H. anatolicum* ticks. Due to the complex post - transcriptional regulatory mechanisms within organisms, gene expression at the transcriptional and translational levels is not always consistent (48). Previous studies have shown moderate or low positive correlations between the transcriptome and proteome (49). Correlation analysis was conducted on overlapping DEGs and DEPs in this study. Across the three comparison groups, immune - related genes and pathways in ticks exhibited different expression trends at the transcriptome and proteome levels. There may be two reasons for this phenomenon. Firstly, during the post - transcriptional stage, mRNA undergoes processing such as splicing, capping, and tailing, which may affect mRNA stability. Thus, despite an increase in transcription levels, the actual availability of mRNA decreases, impacting protein synthesis. Secondly, the translated proteins may be regulated by post - translational modifications, which can further affect protein stability, activity, and localization. Given these complex post - transcriptional and post - translational regulatory mechanisms, it is believed that sequencing analysis at multiple time points after puncture induction may more comprehensively capture these dynamic changes, leading to better transcriptome and proteome data. Secondly, we analyzed the entire tick body. While this approach offers comprehensive gene and protein expression data, it doesn’t reveal the specific immune responses of particular tissues or cell types. Future studies could isolate specific tissues or cell types, such as hemocytes, midgut, and salivary glands, to conduct more detailed analyses.

Nonetheless, these results still demonstrate the consistency of these molecular pathways across other species and advance our understanding of the immune - mediated defense mechanisms in *H. anatolicum*. To comprehend the persistence of altered gene expression and functional pathways over extended periods, it would be beneficial to explore the long - term consequences and dynamics of these responses. Determining the functional importance of the identified genes and pathways requires additional experimental validation, despite the wealth of information provided by functional annotation and pathway enrichment analyses.

## 5 Conclusion

This study conducted a comprehensive analysis of the proteomic and transcriptomic changes in the *H. anatolicum* adult female ticks in response to stimulation by *S. aureus* or *P. mirabilis*, revealing a series of mRNAs and proteins involved in innate immune responses. Significant changes occurred in biological processes related to cellular senescence, MAPK signaling pathways, NF - κB signaling pathways, Toll and IMD signaling pathways in bacterial invasion to *H. anatolicum*. A large number of AMP genes and immune - related signaling pathways genes played crucial roles in the innate immune defense of the tick species. The proteomic and transcriptomic data resources provided by this study may offer valuable insights for a deeper understanding of the immune defense mechanisms of ticks and the acquisition of sequence information for antimicrobial proteins.

## Data Availability

The data presented in the study are deposited in the NCBI repository, accession number PRJNA1189655. Regarding the mass spectrometry proteomics data, they have been deposited in the ProteomeXchange Consortium via the PRIDE partner repository, dataset identifier PXD032781.
