# Supplementary material for: Integrated transcriptomic and proteomic analysis of the immune response in Hyalomma anatolicum to bacterial invasion
Source: Front Immunol. 2025 Sep 4;16:1576721. doi: 10.3389/fimmu.2025.1576721 (PMC12443844; doi:10.3389/fimmu.2025.1576721)
Supplement: Supplementary file 1 [file Supplementaryfile1.docx]

Supplementary Material

# 2 Supplementary Figures and Tables

## 2.1 Supplementary Figures

**
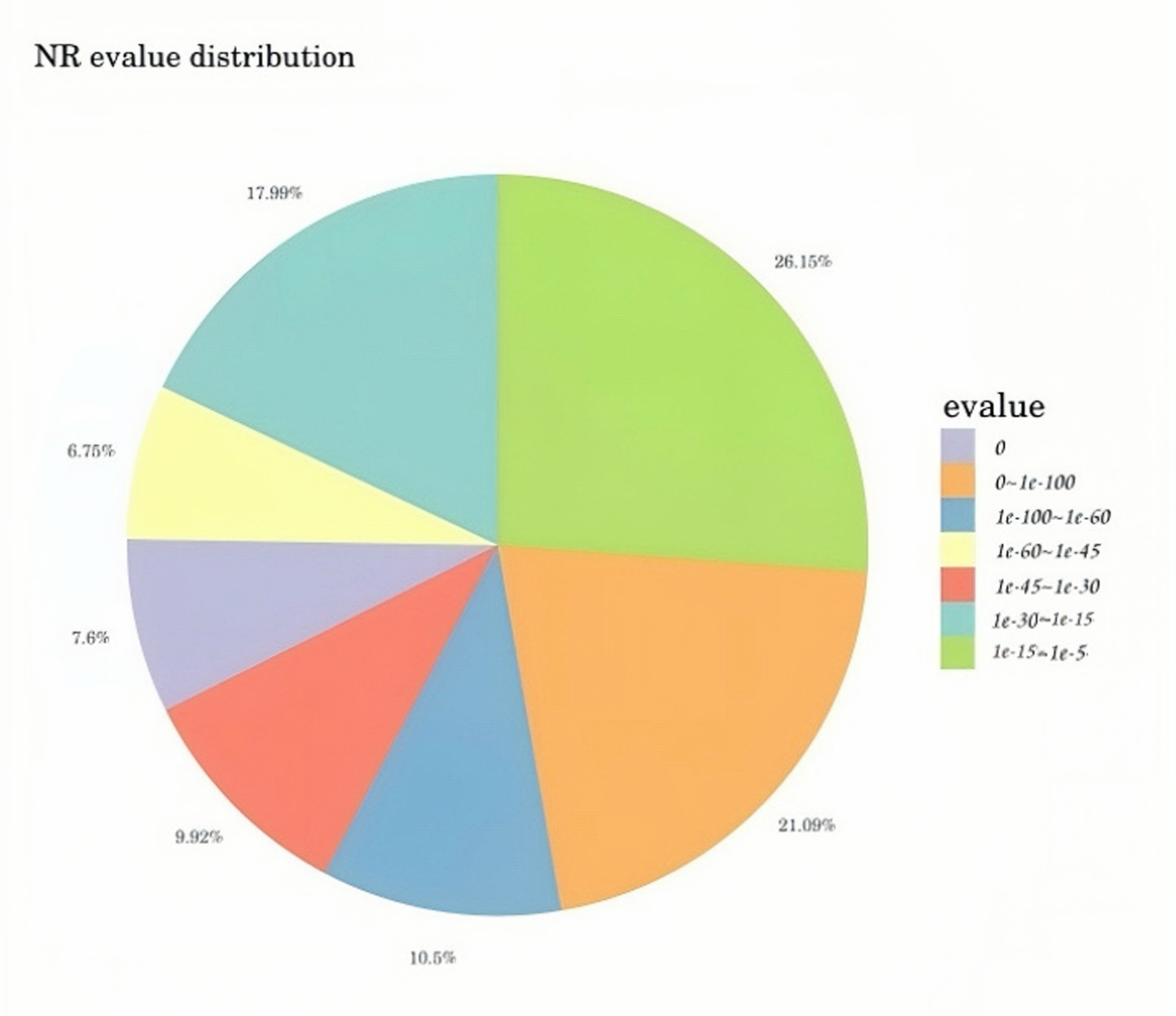
**

**Supplementary Figure 1.** Distribution of E-values from gene alignment based on the NR database.


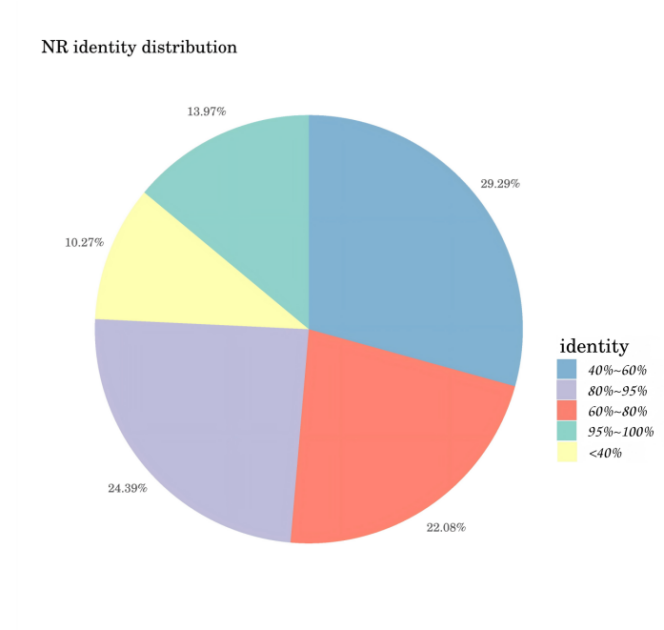


**Supplementary Figure 2.** Distribution of gene sequence identity based on the NR database.


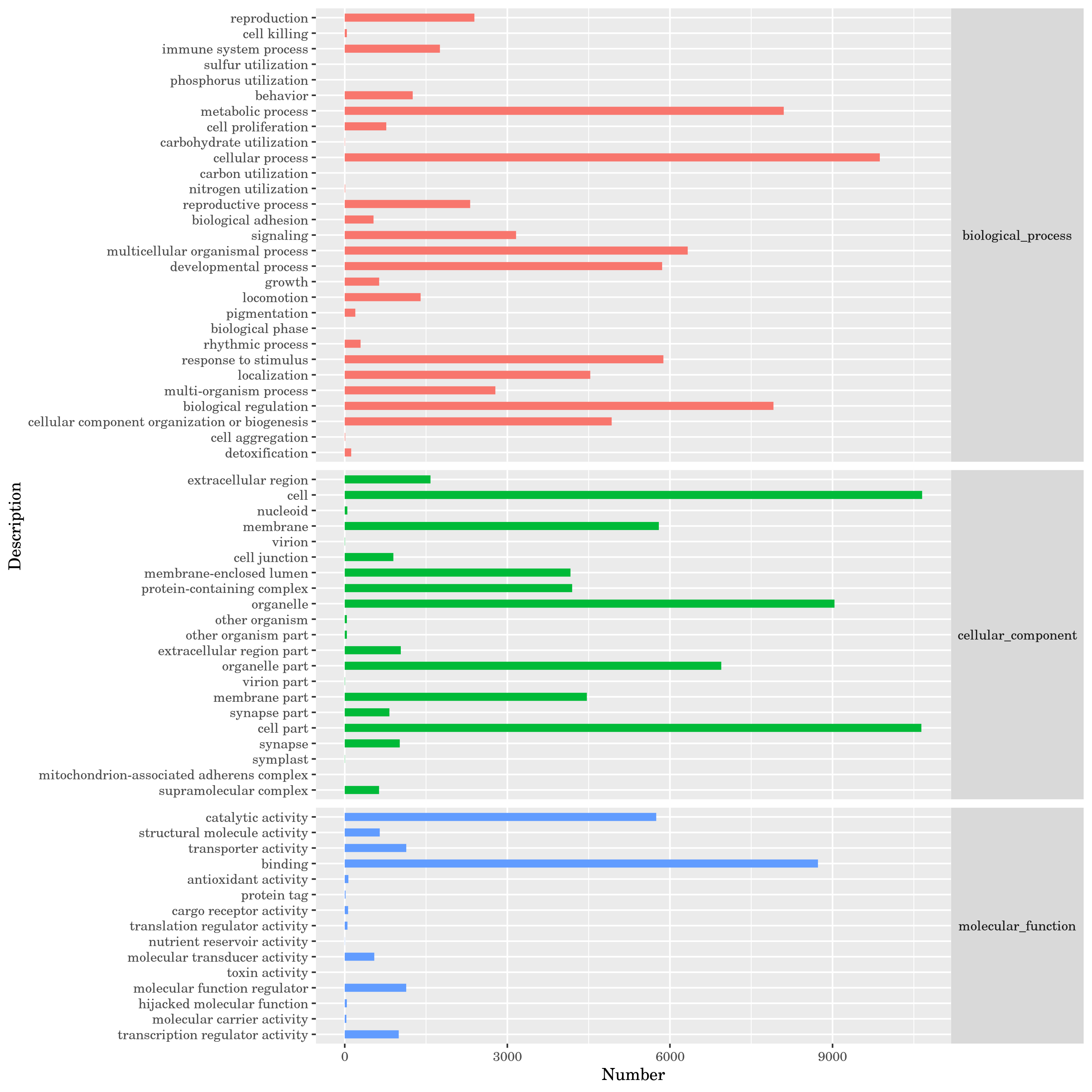


**Supplementary Figure 3.** Gene Ontology (GO) functional classification statistics.


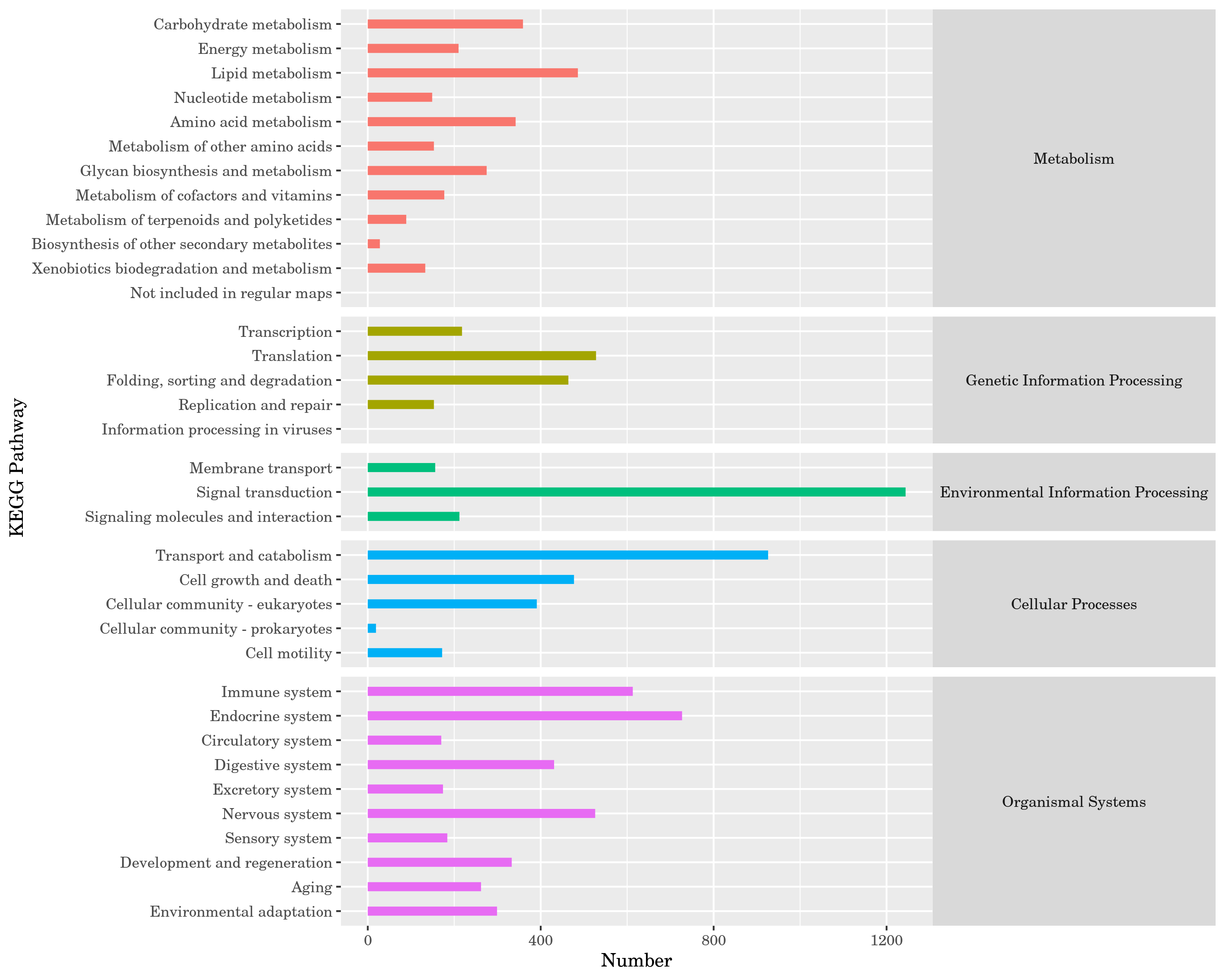


**Supplementary Figure 4.** KEGG metabolic pathway and functional category enrichment analysis.


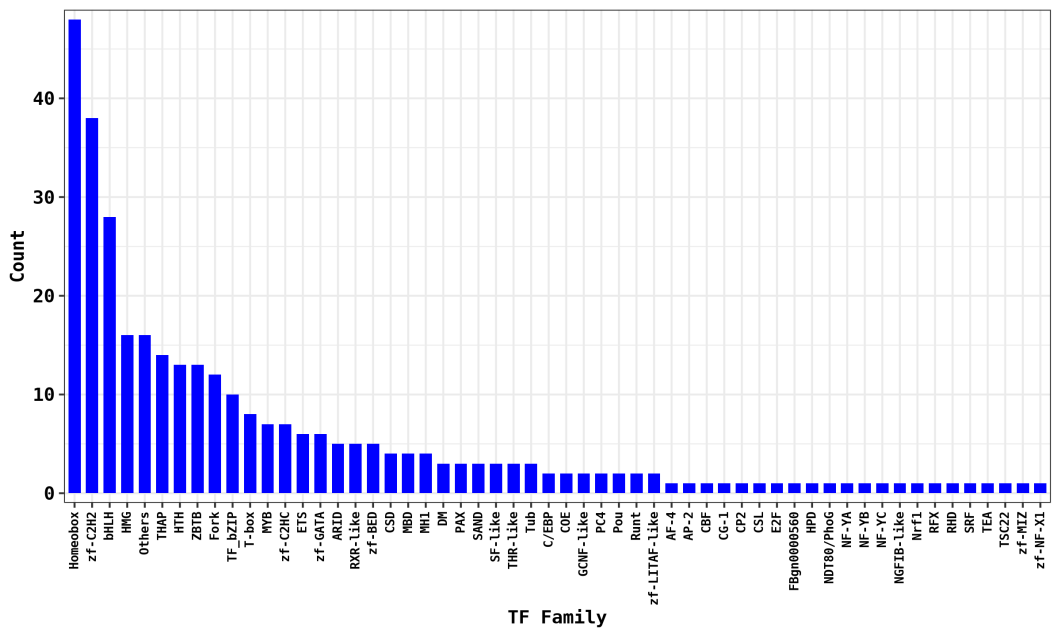


**Supplementary Figure 5.** Statistical chart of transcription factor families.


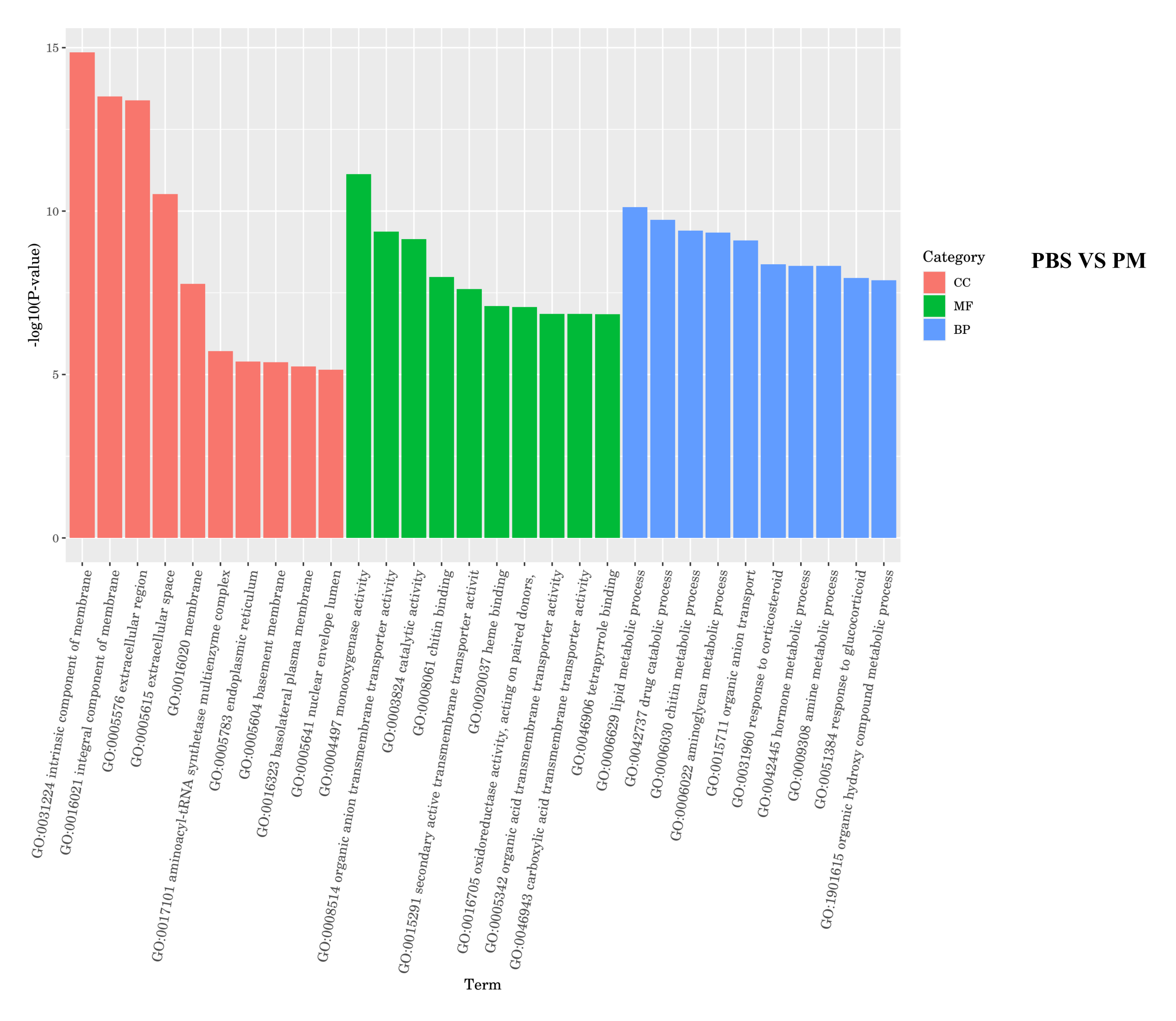

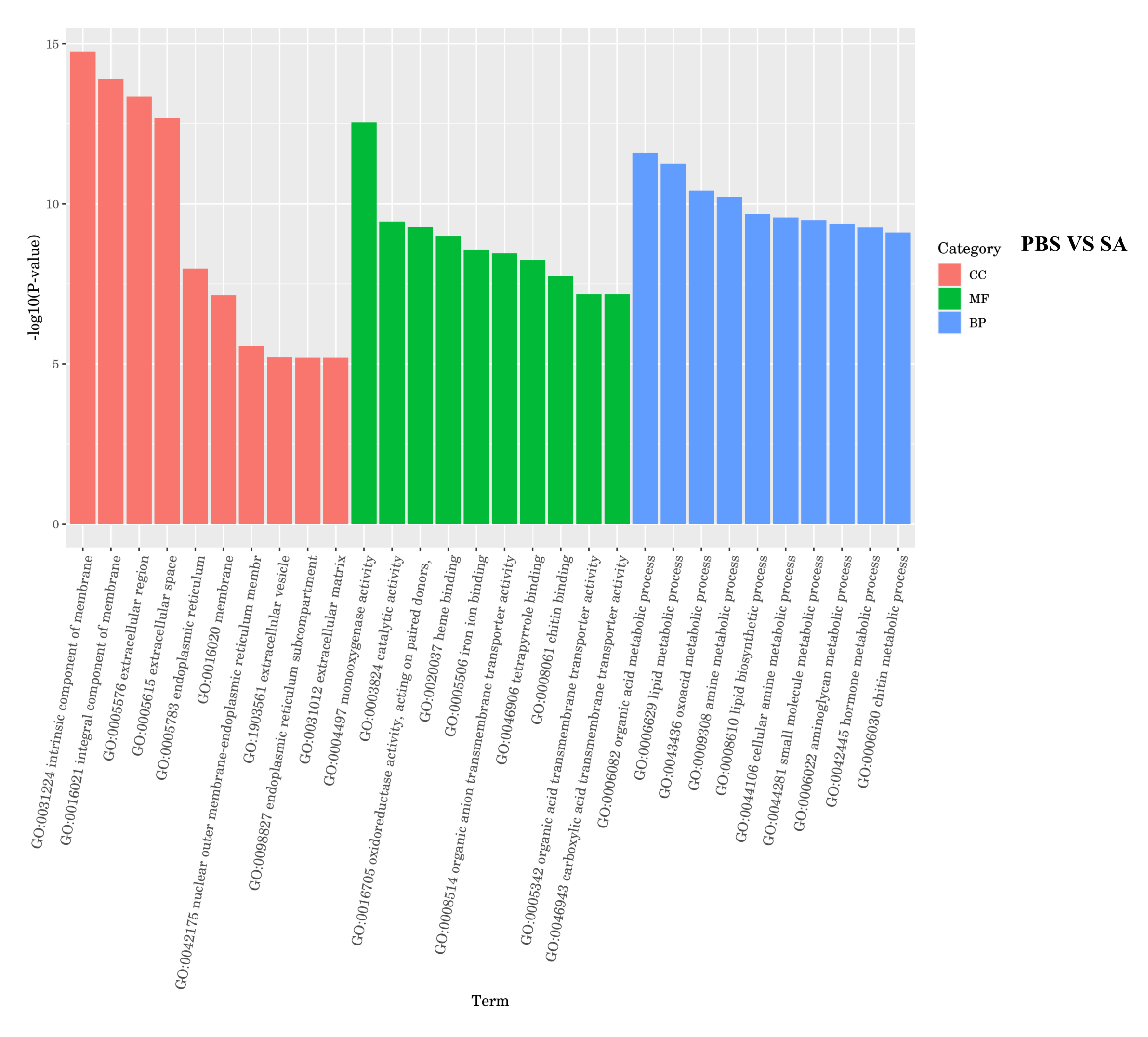

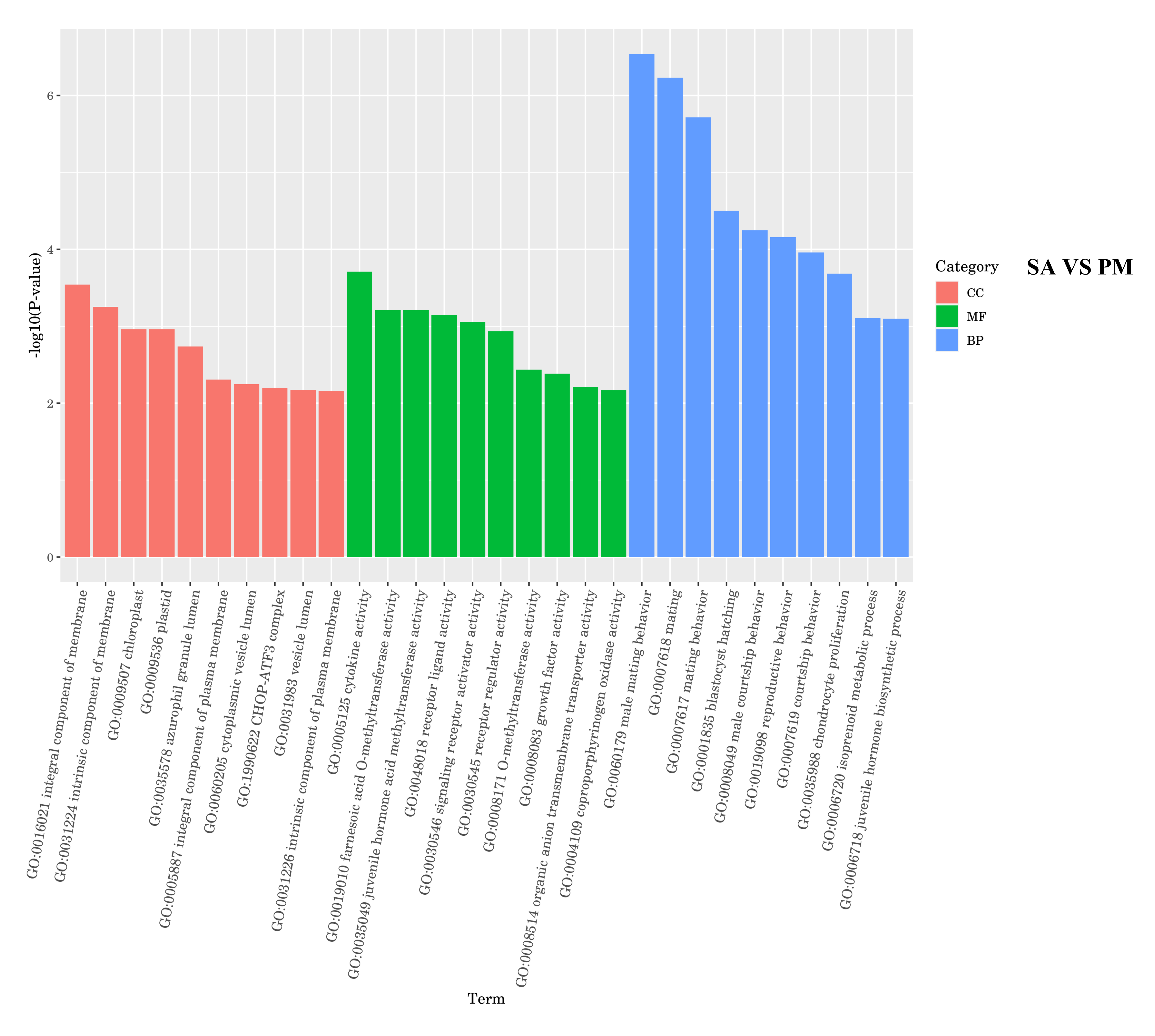


**Supplementary Figure 6.** Bar plot of GO enrichment analysis.


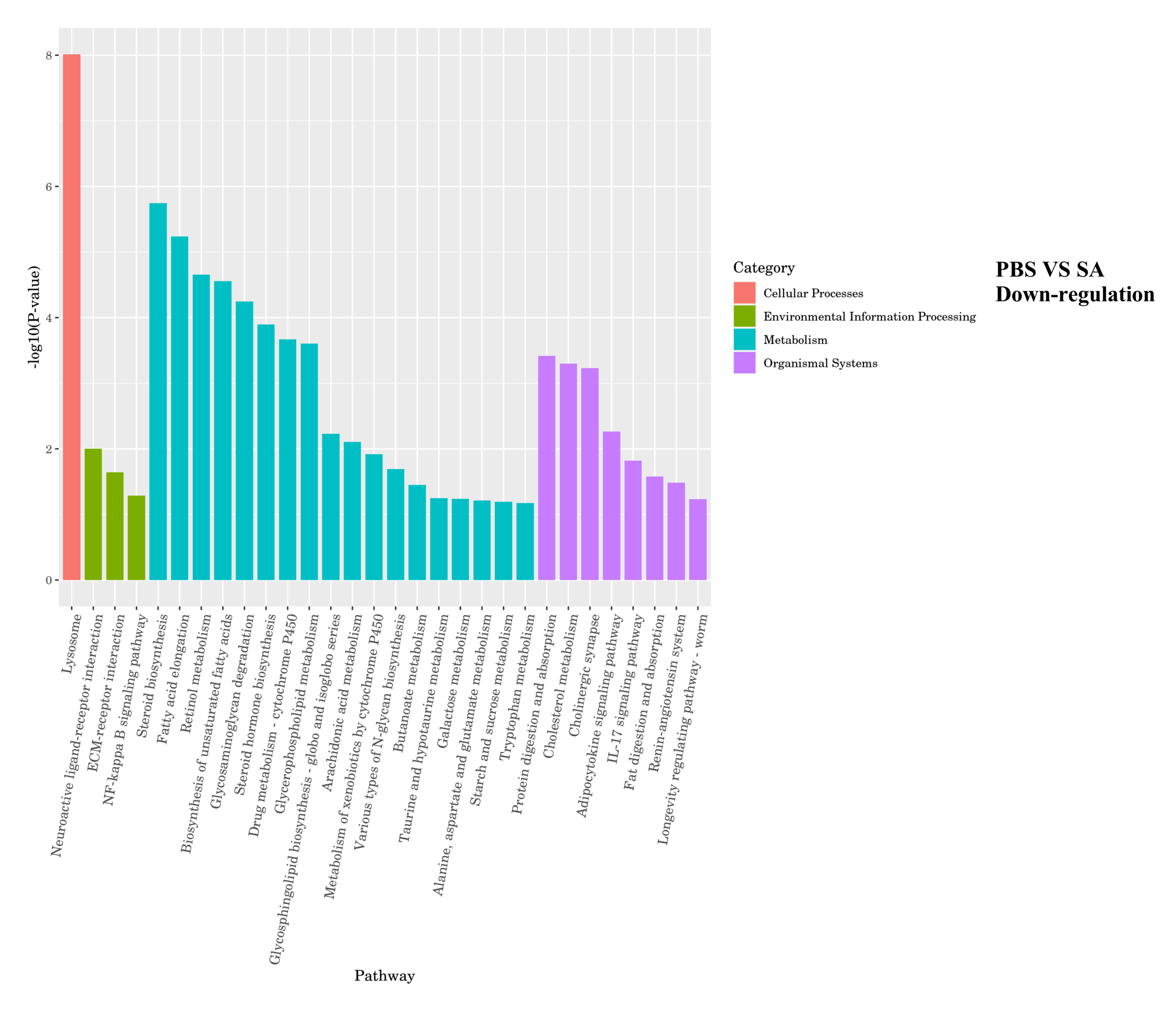

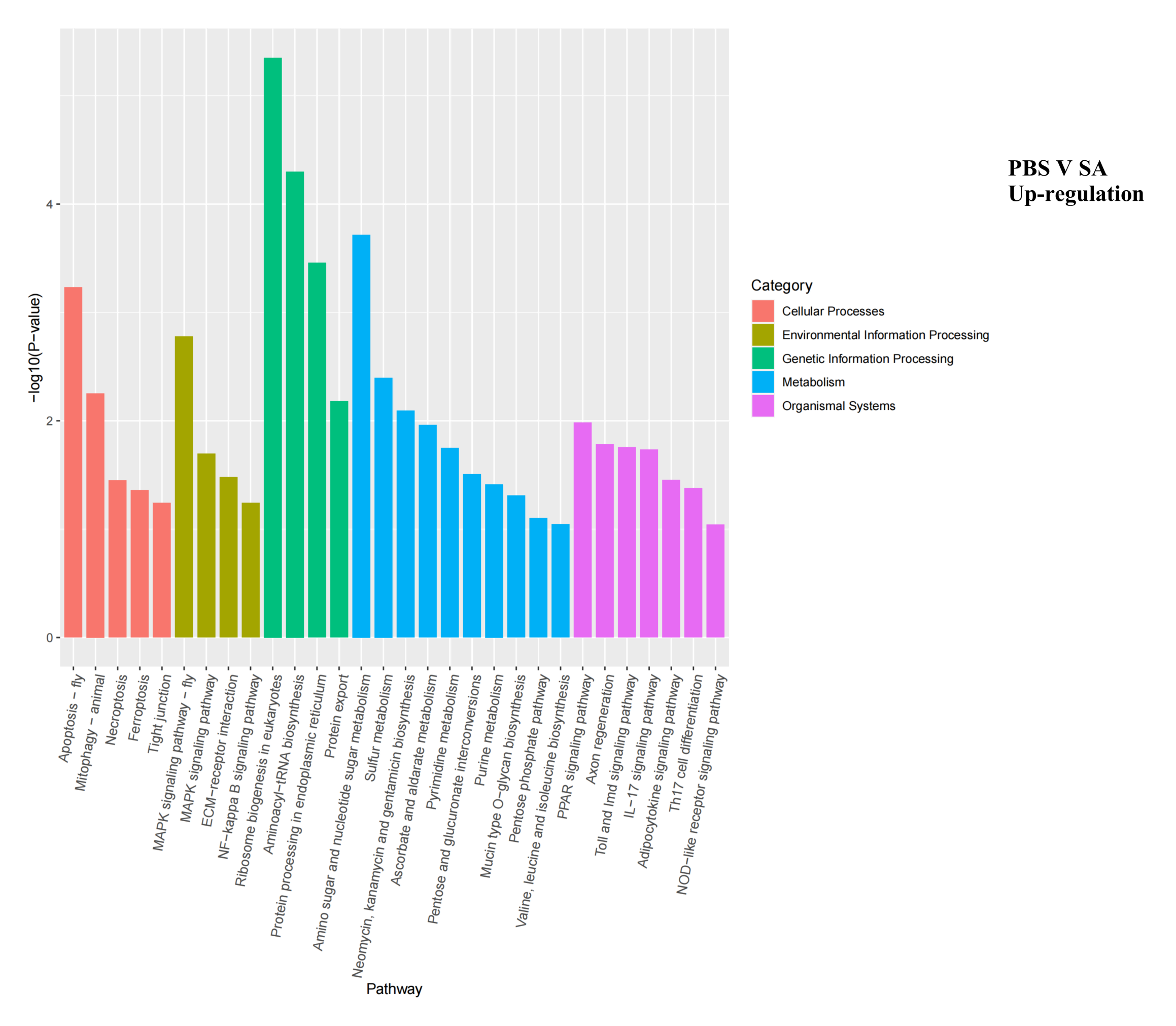

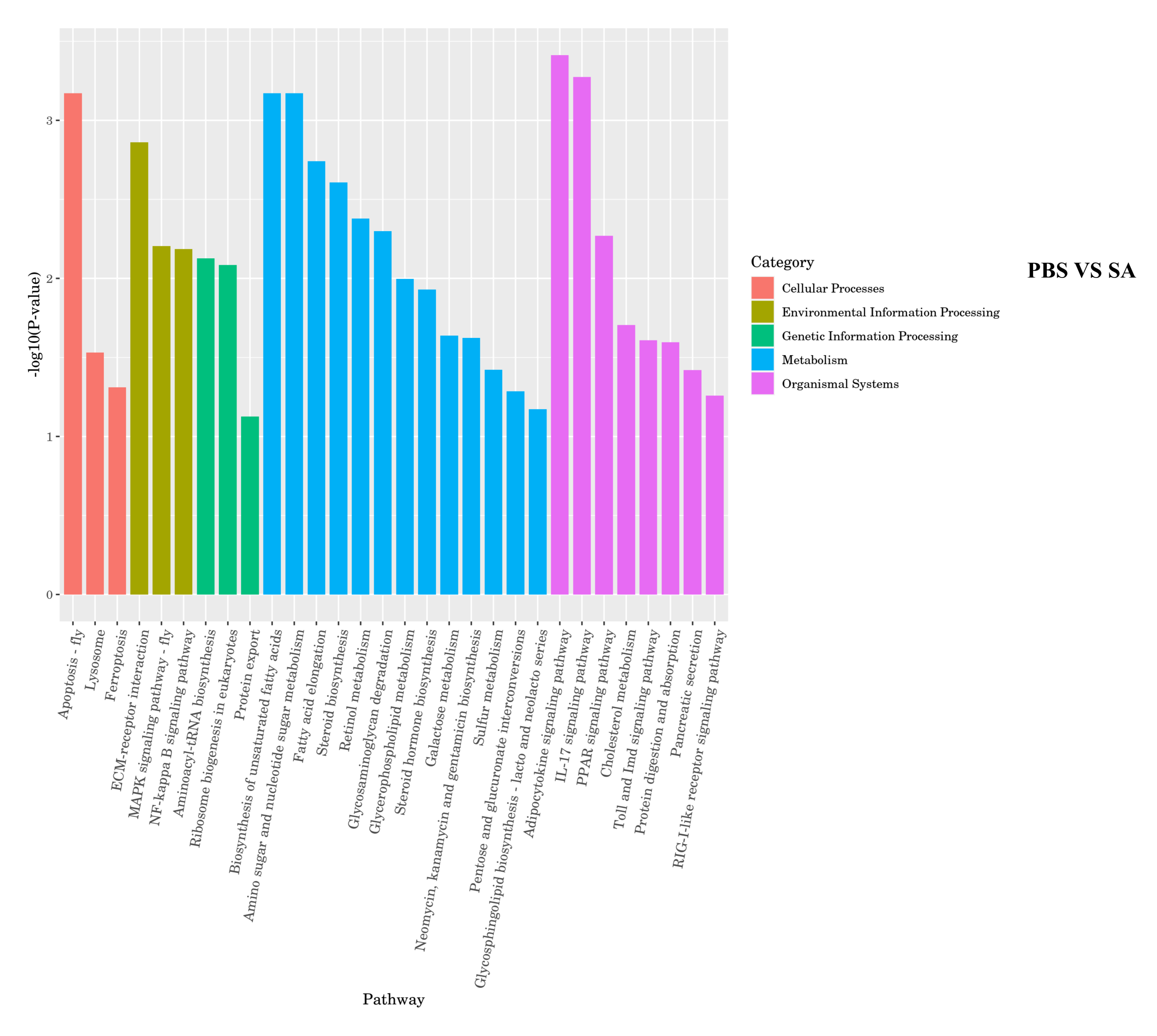

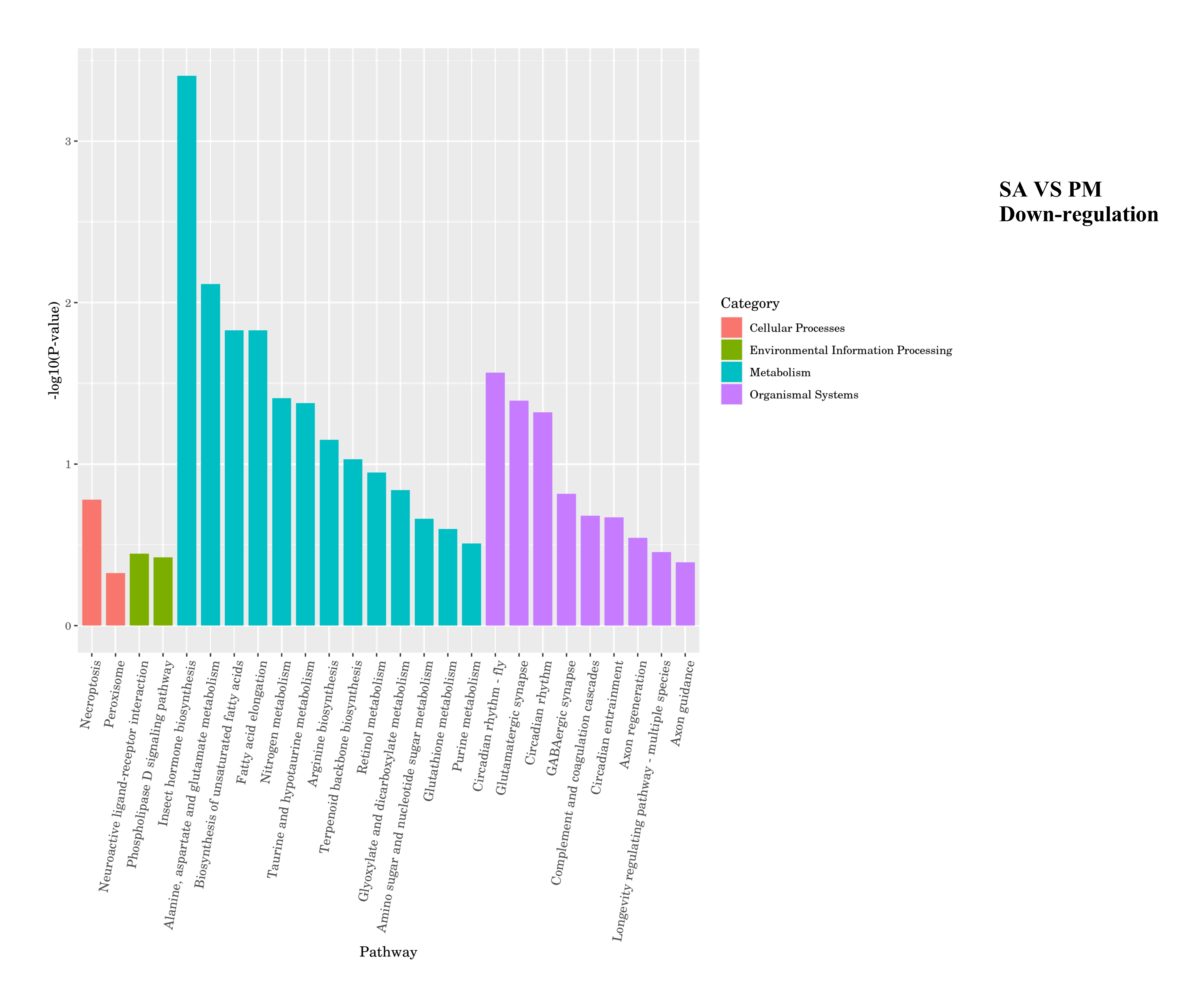

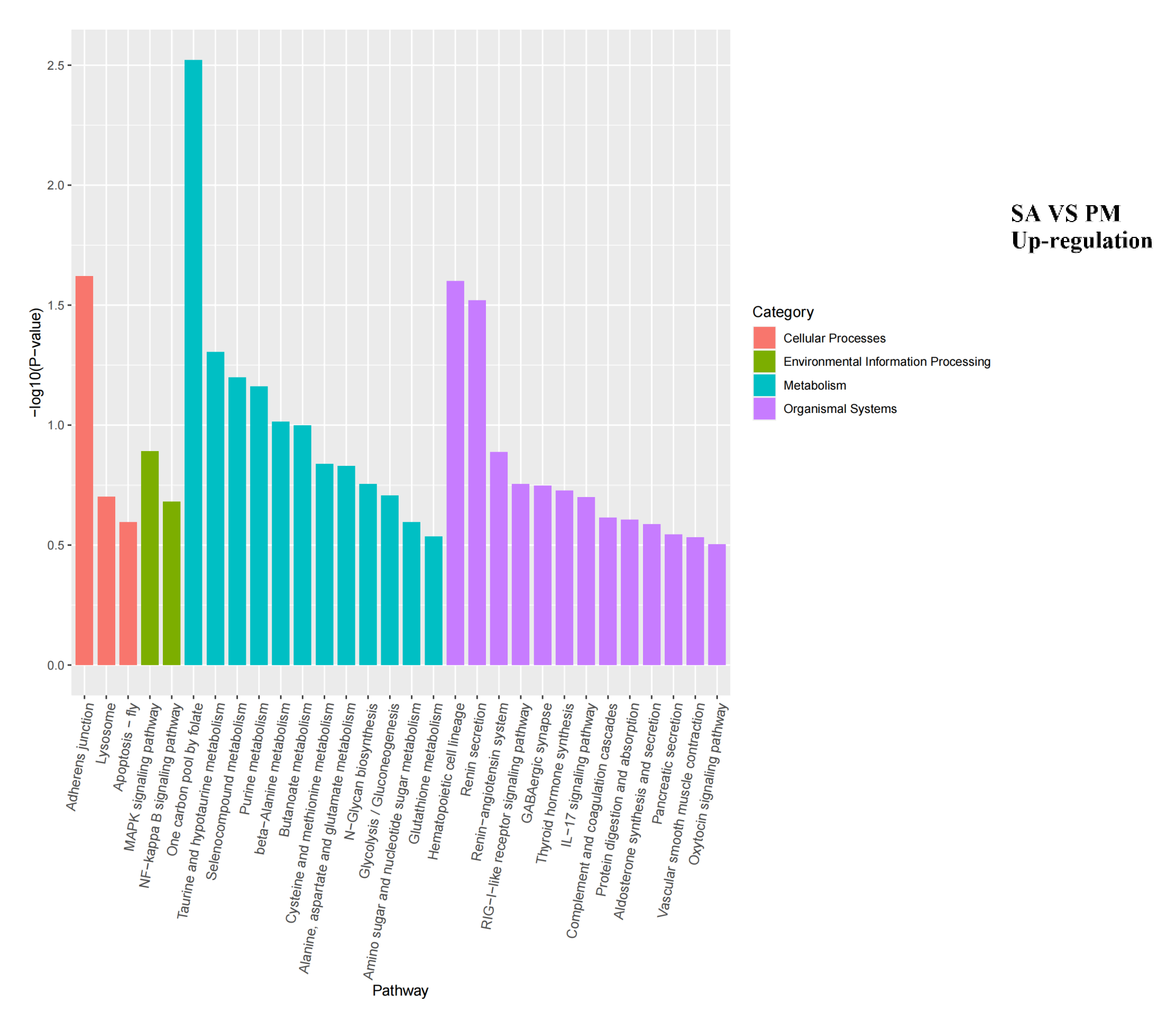

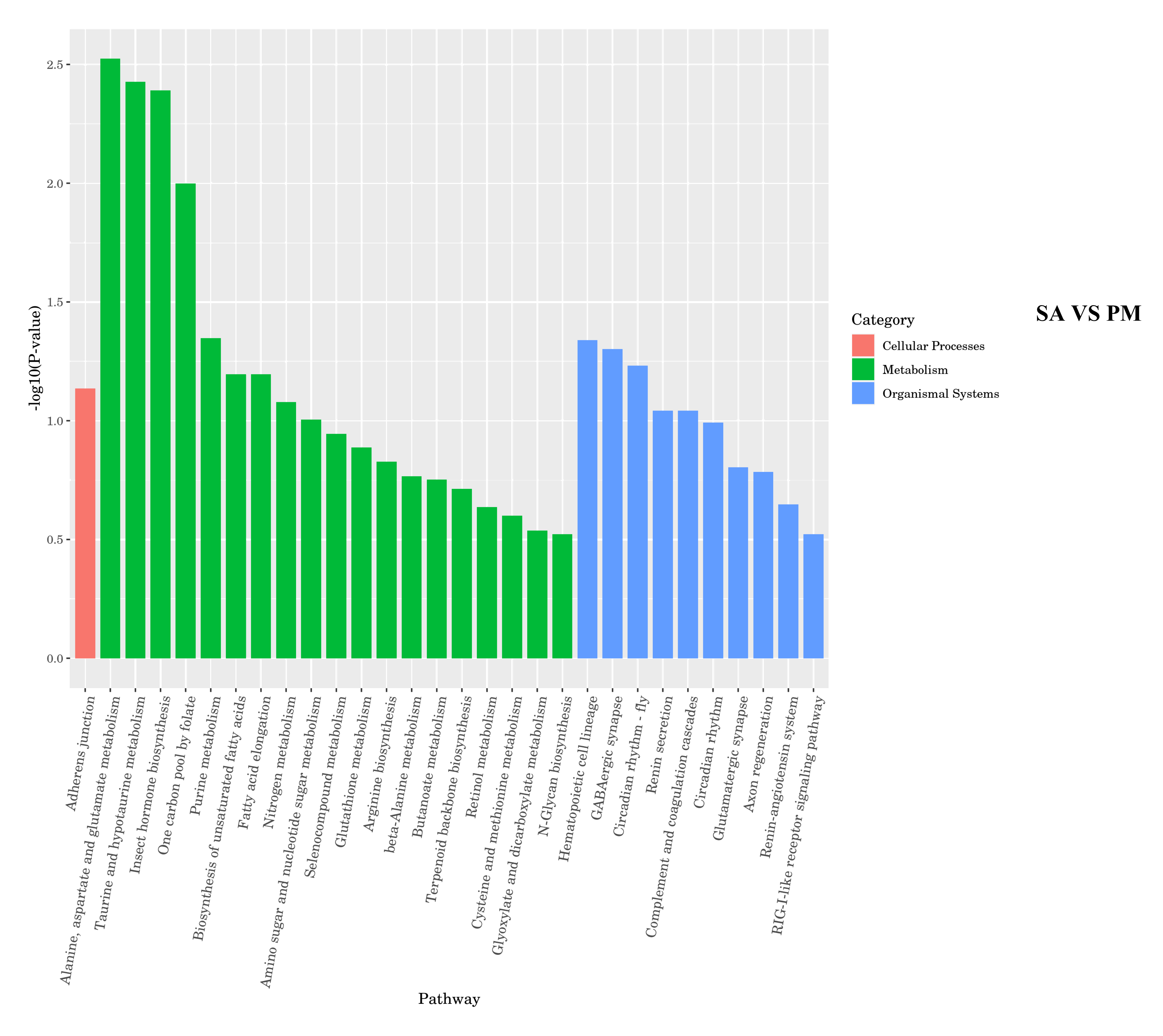

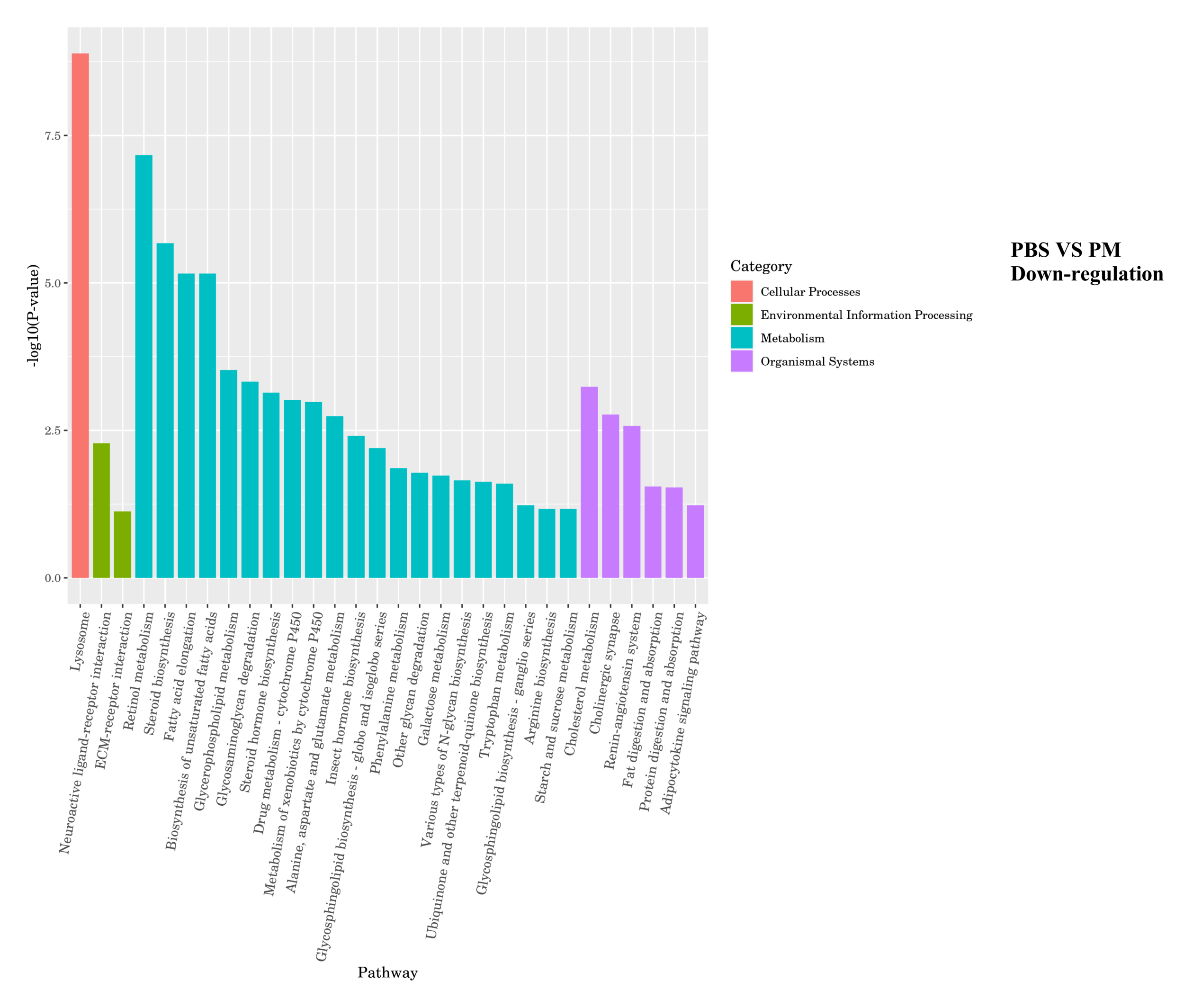

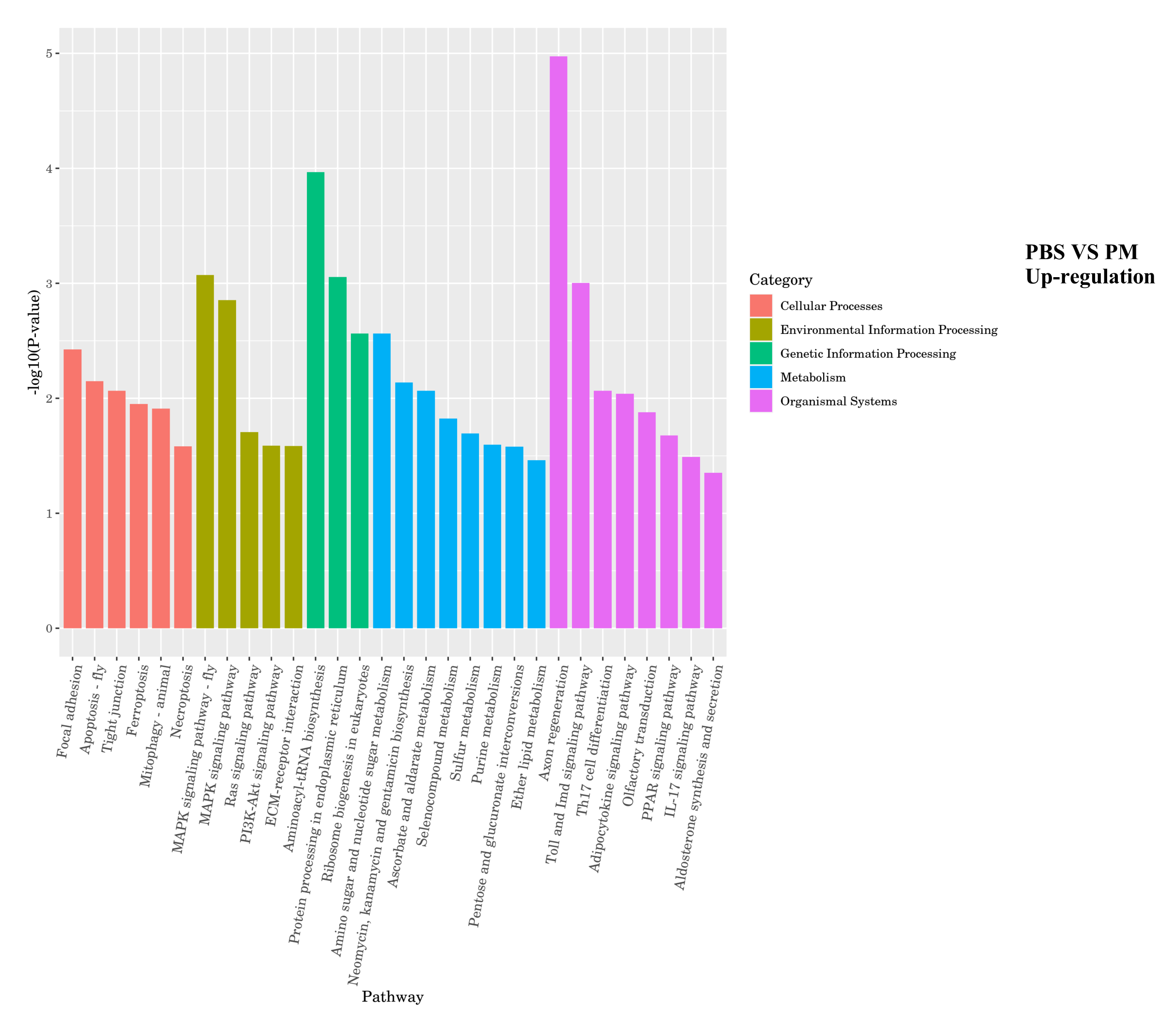

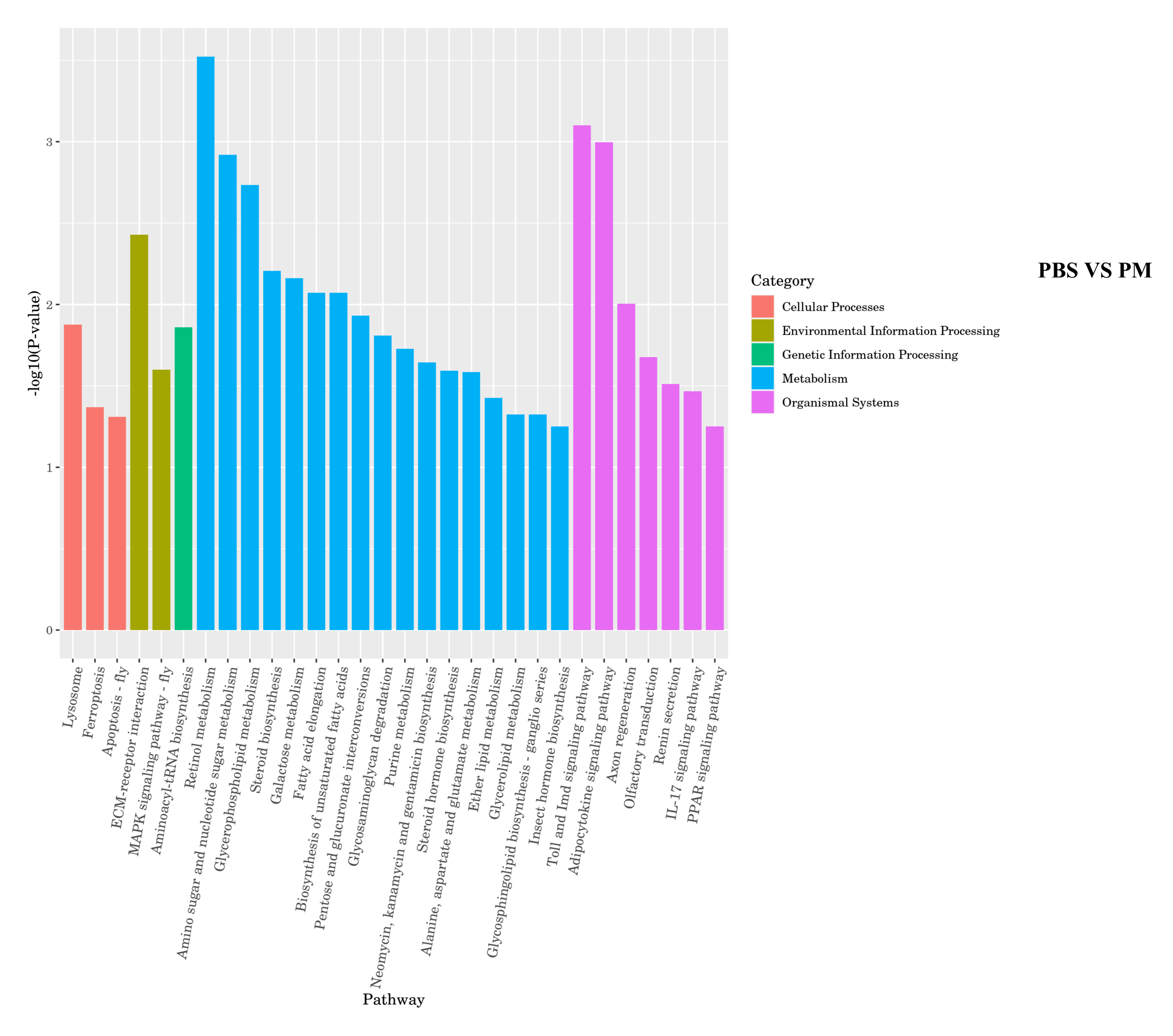

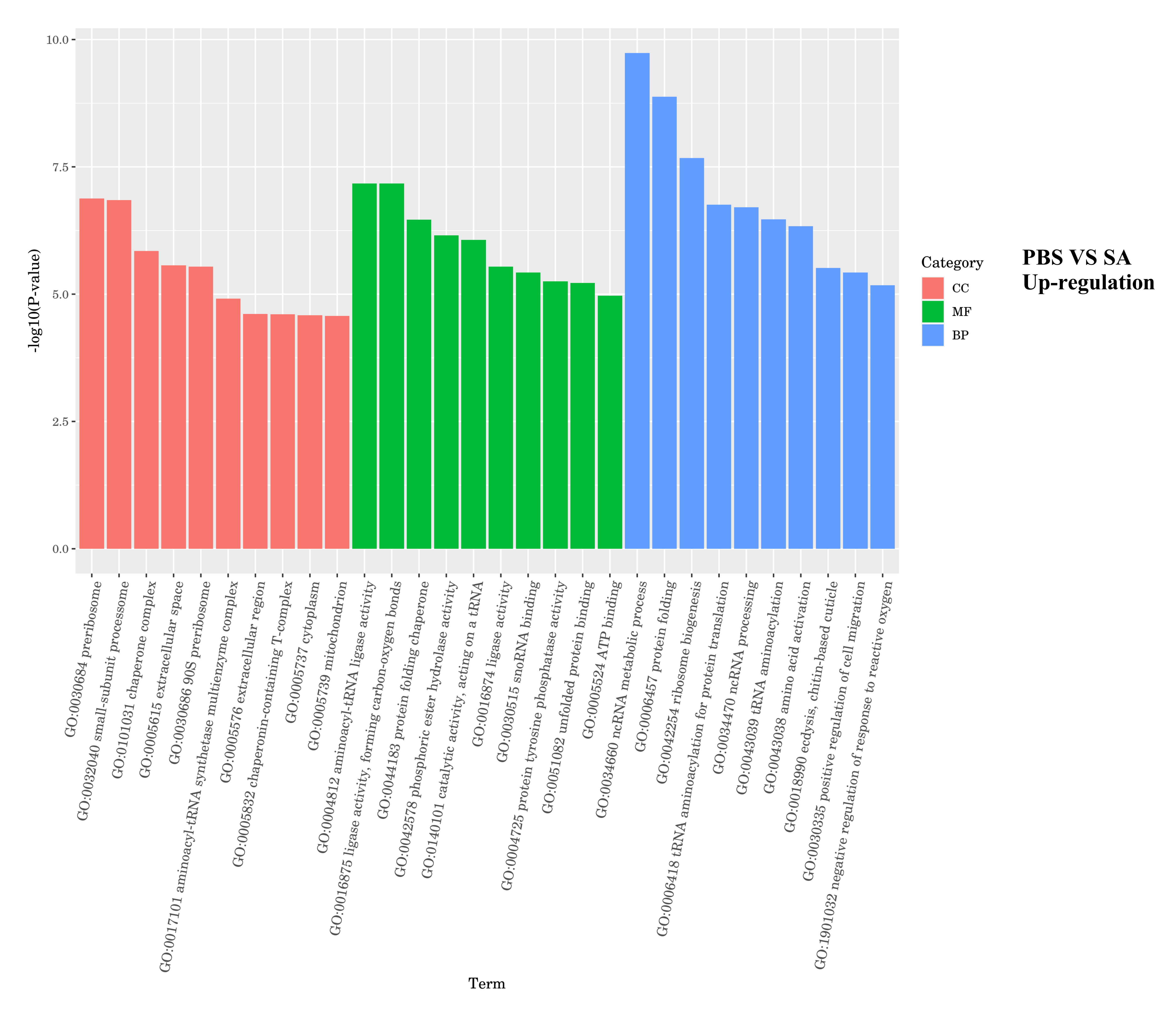

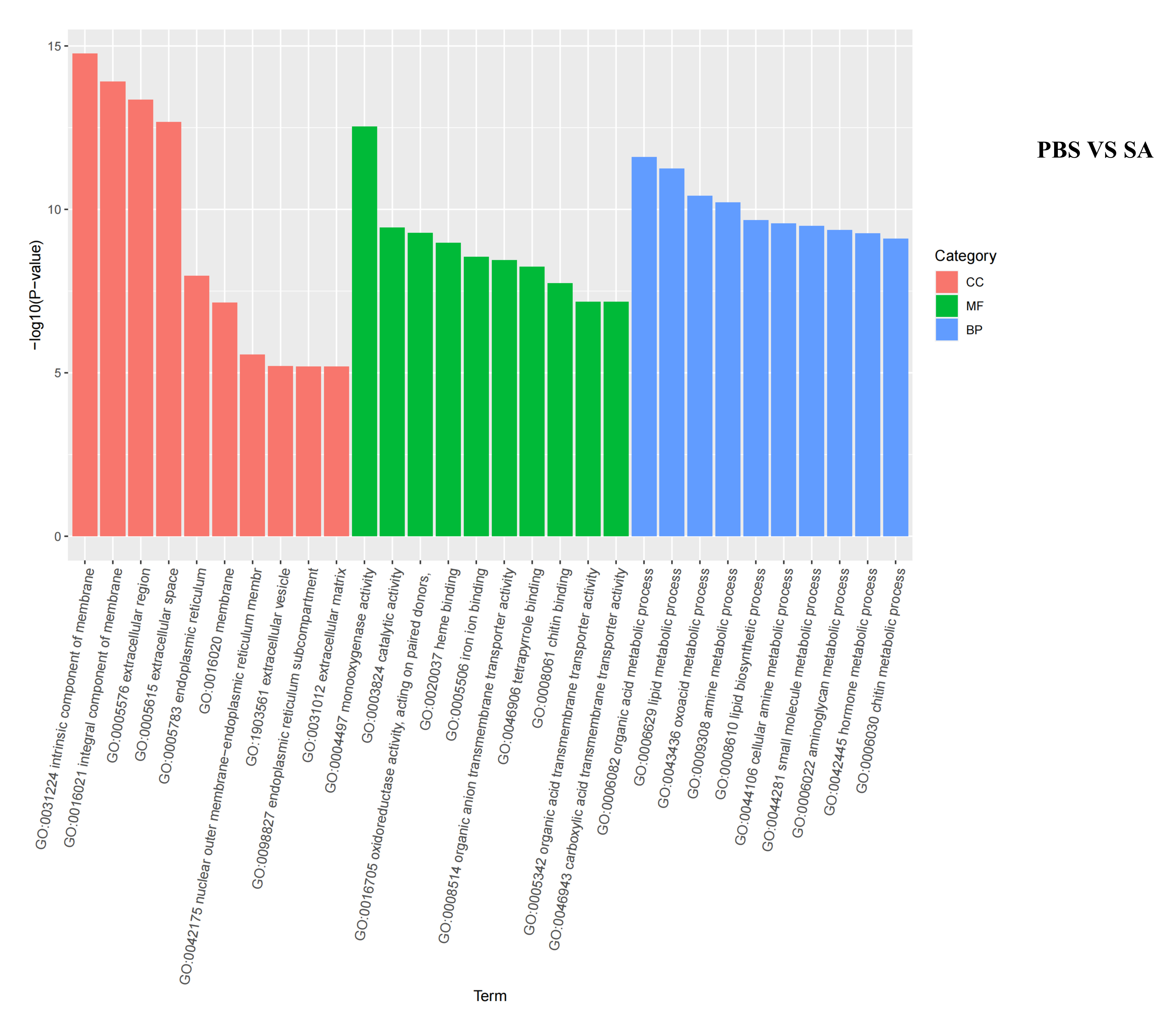

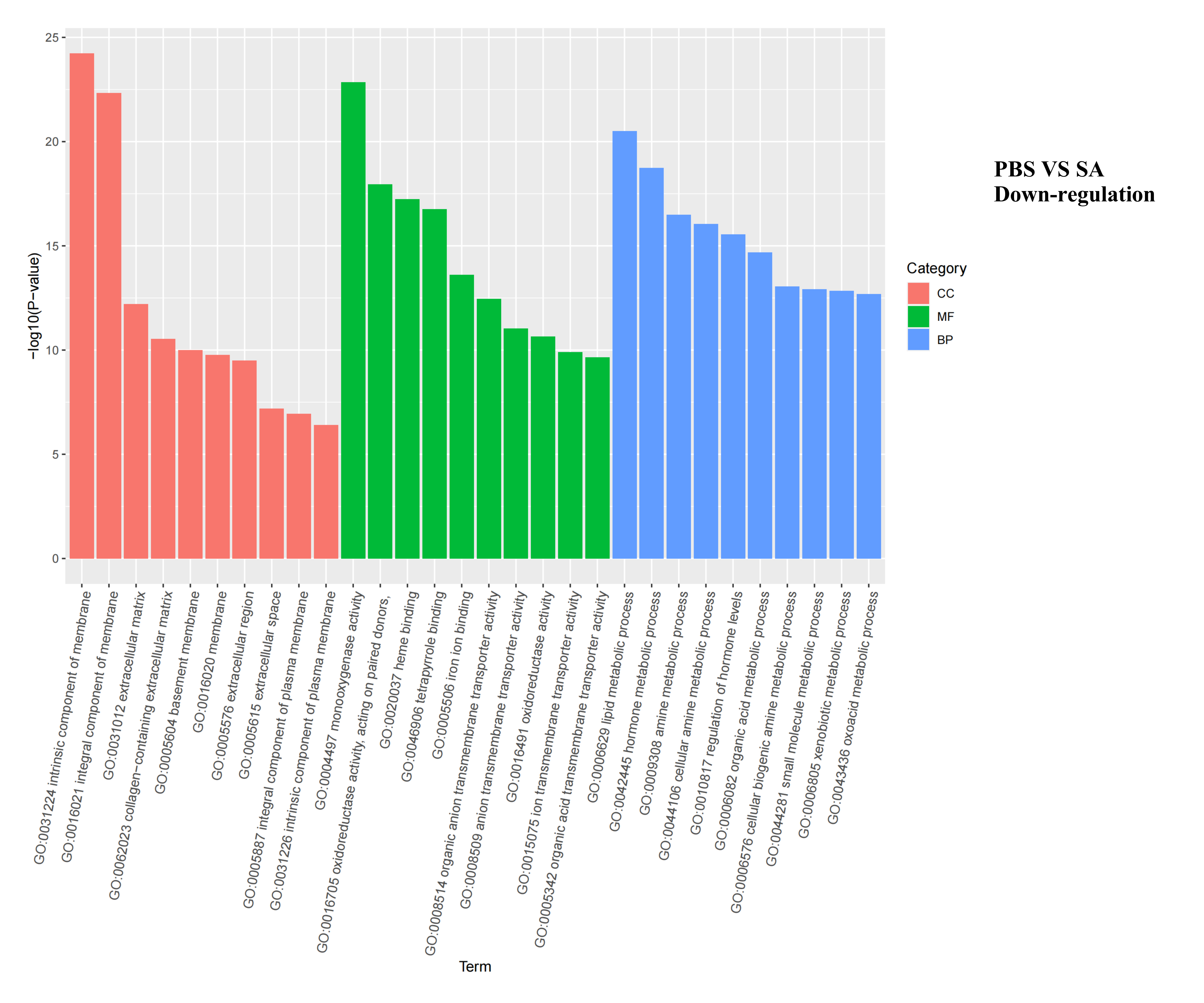

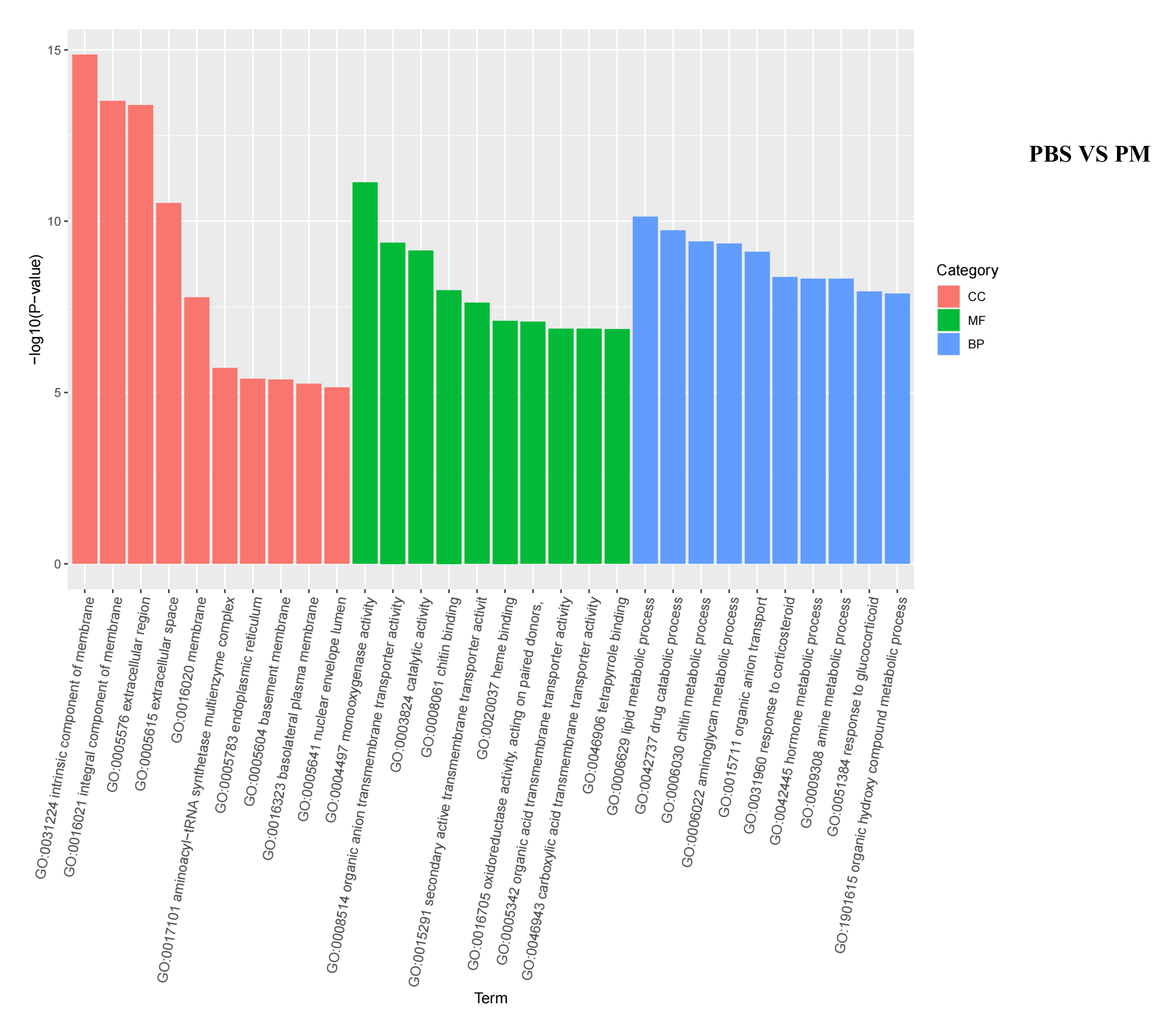

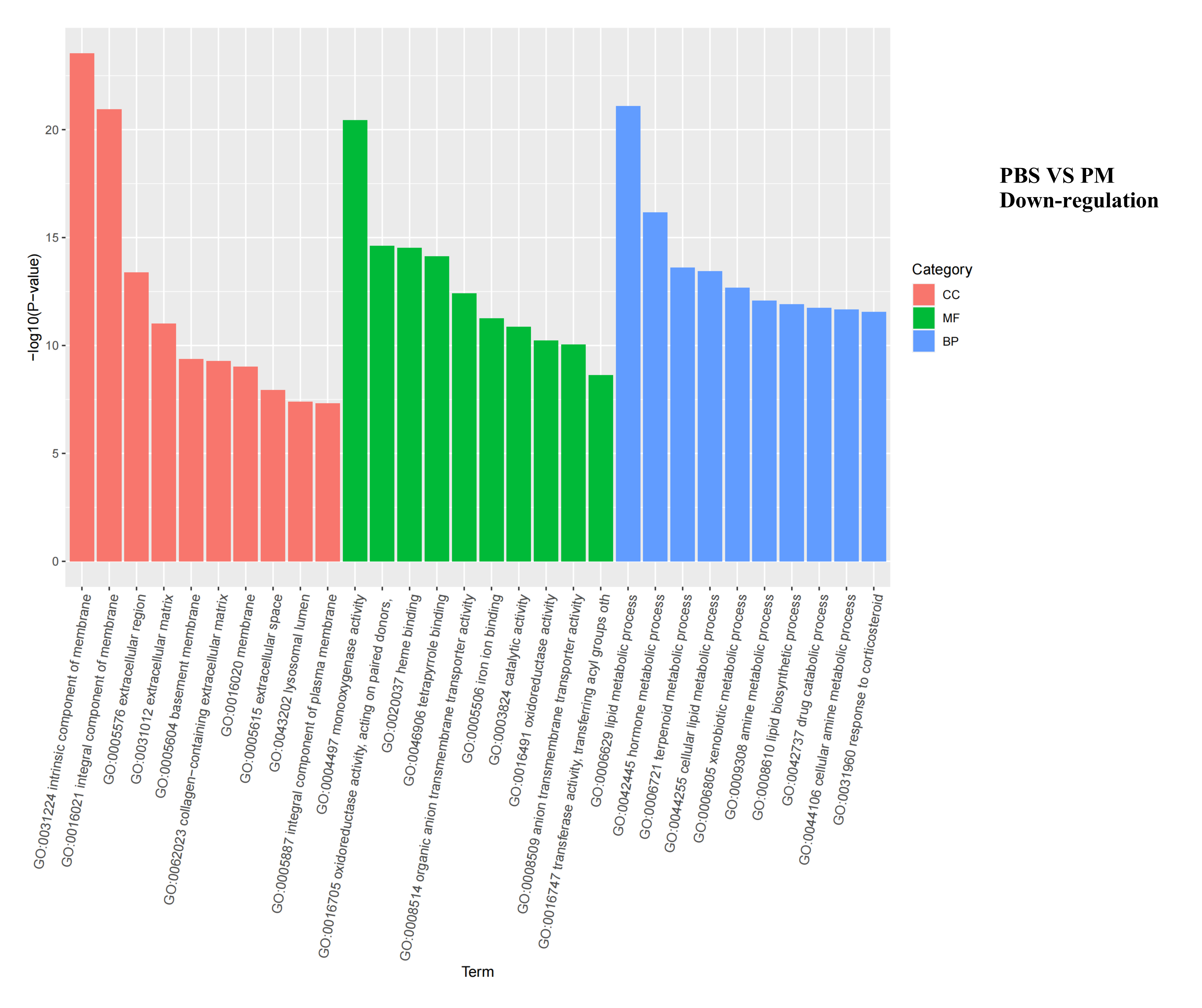

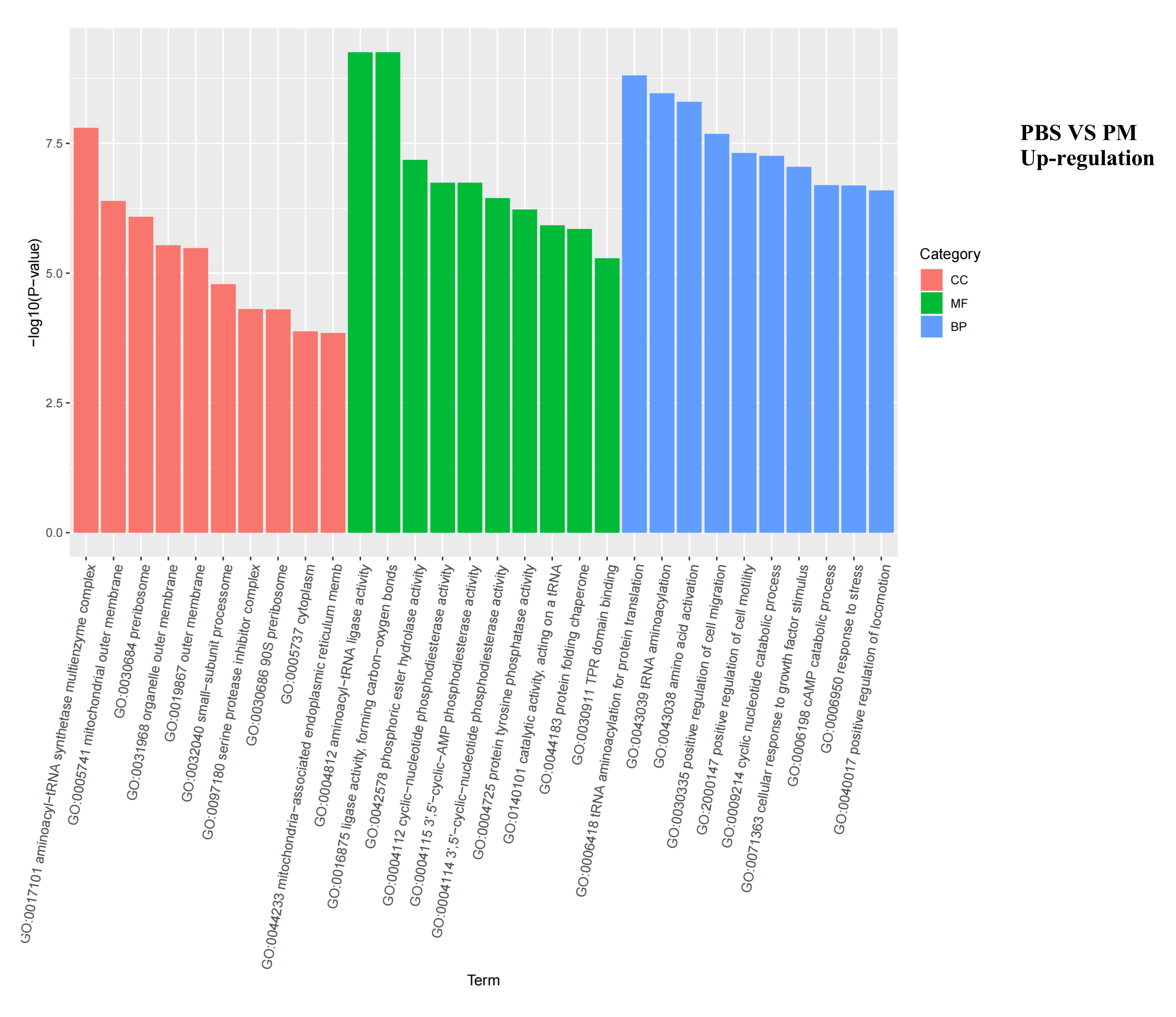

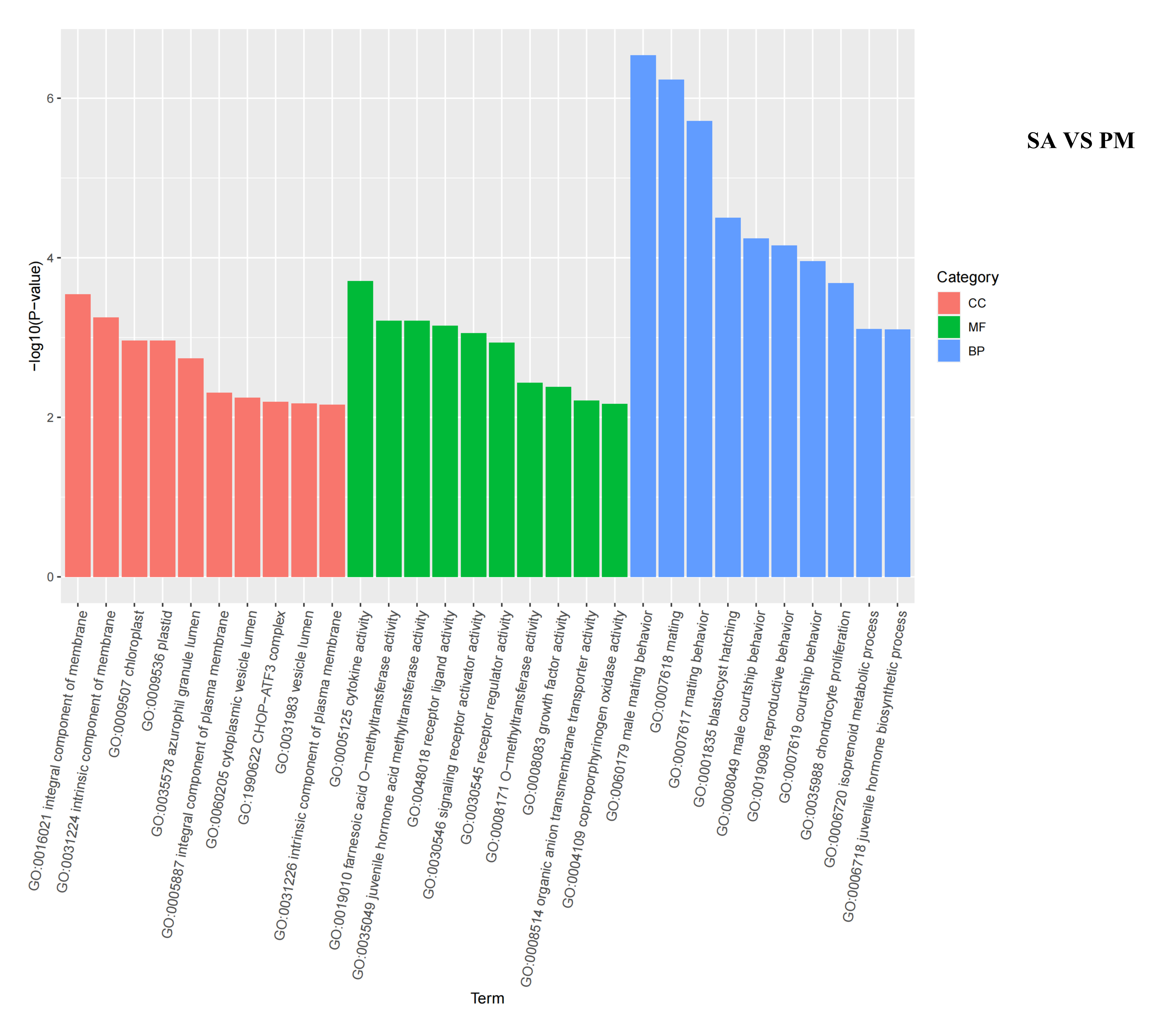

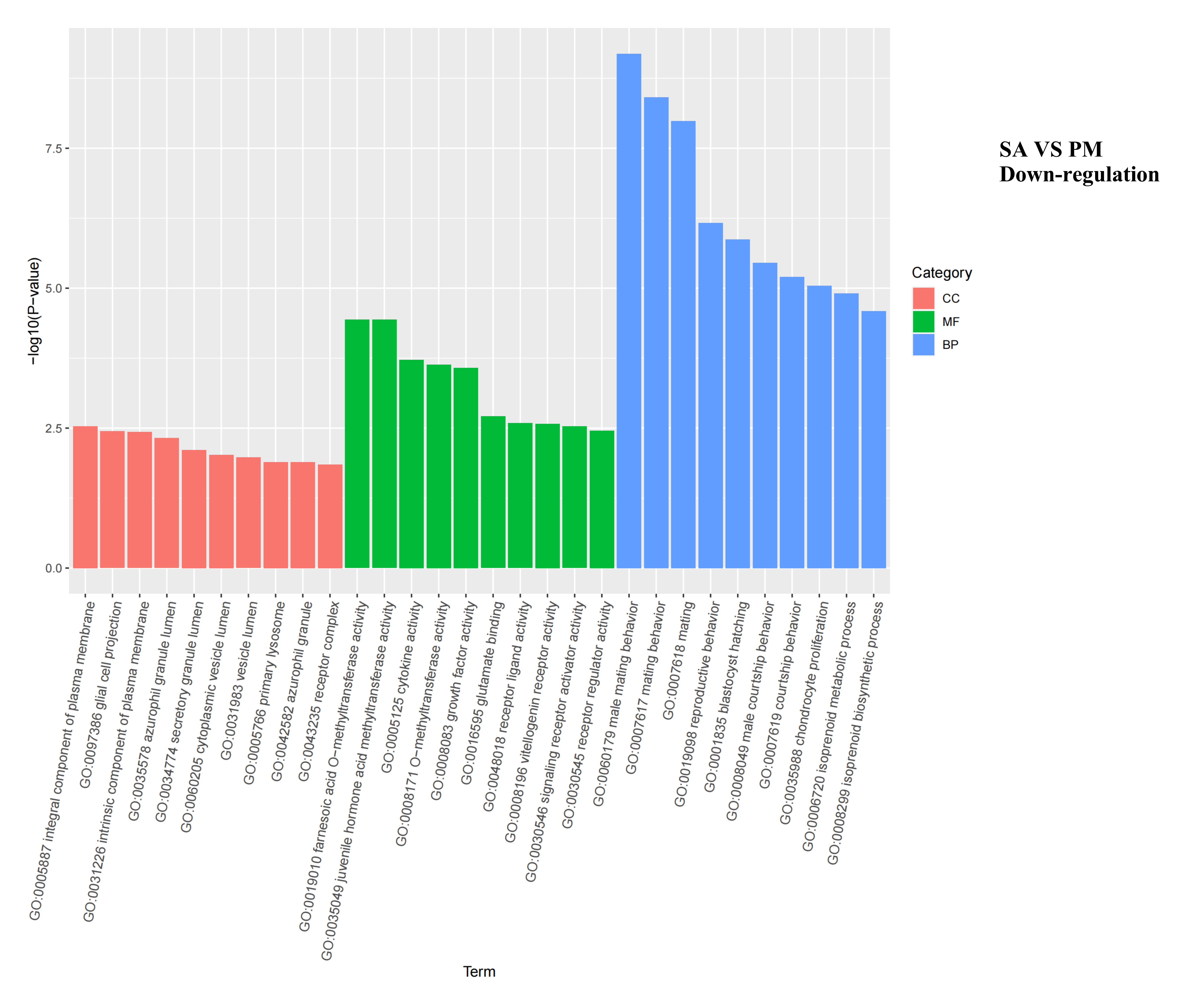

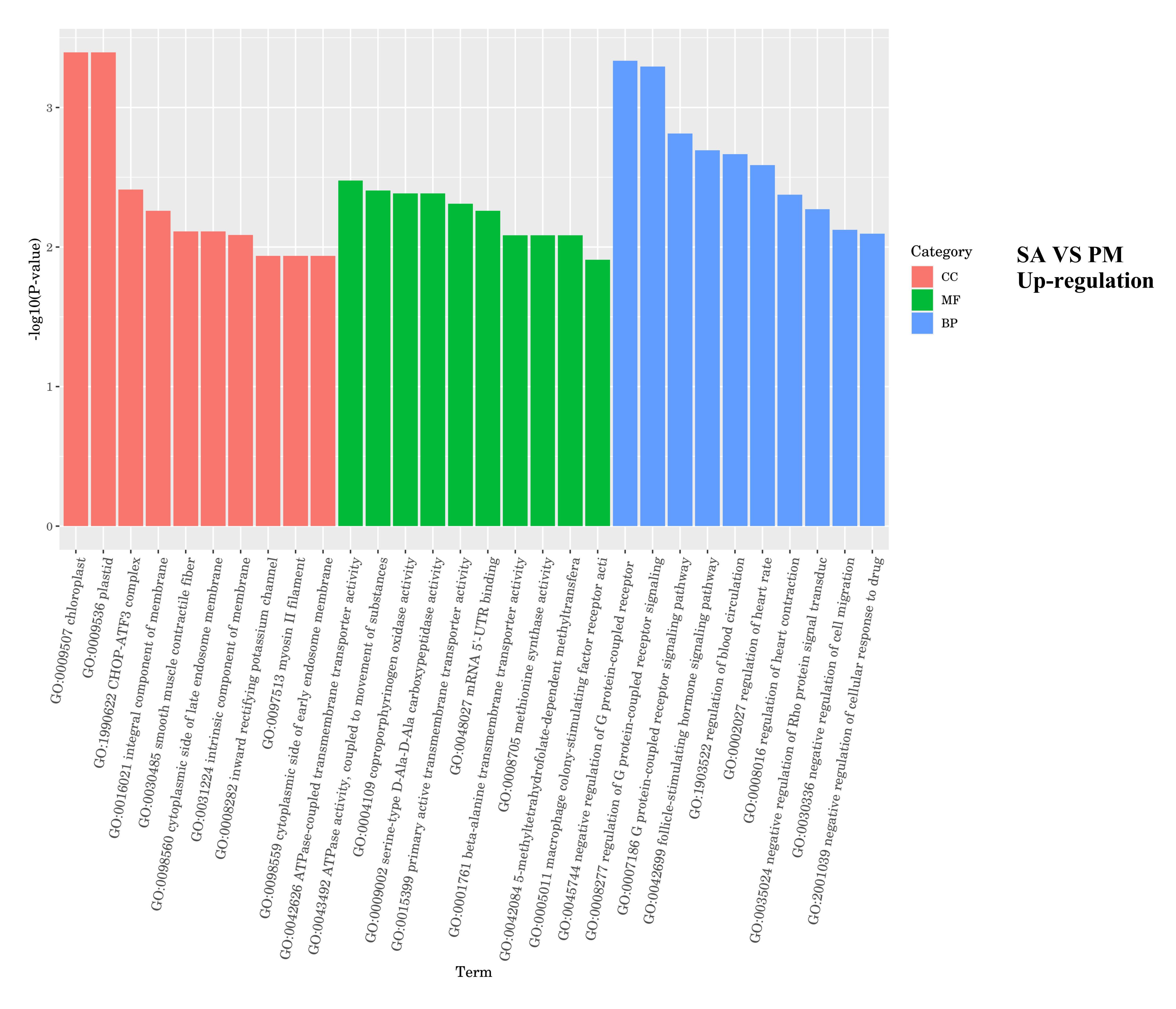


**Supplementary Figure 7.** GO and KEGG enrichment analysis of differentially expressed genes (DEGs).


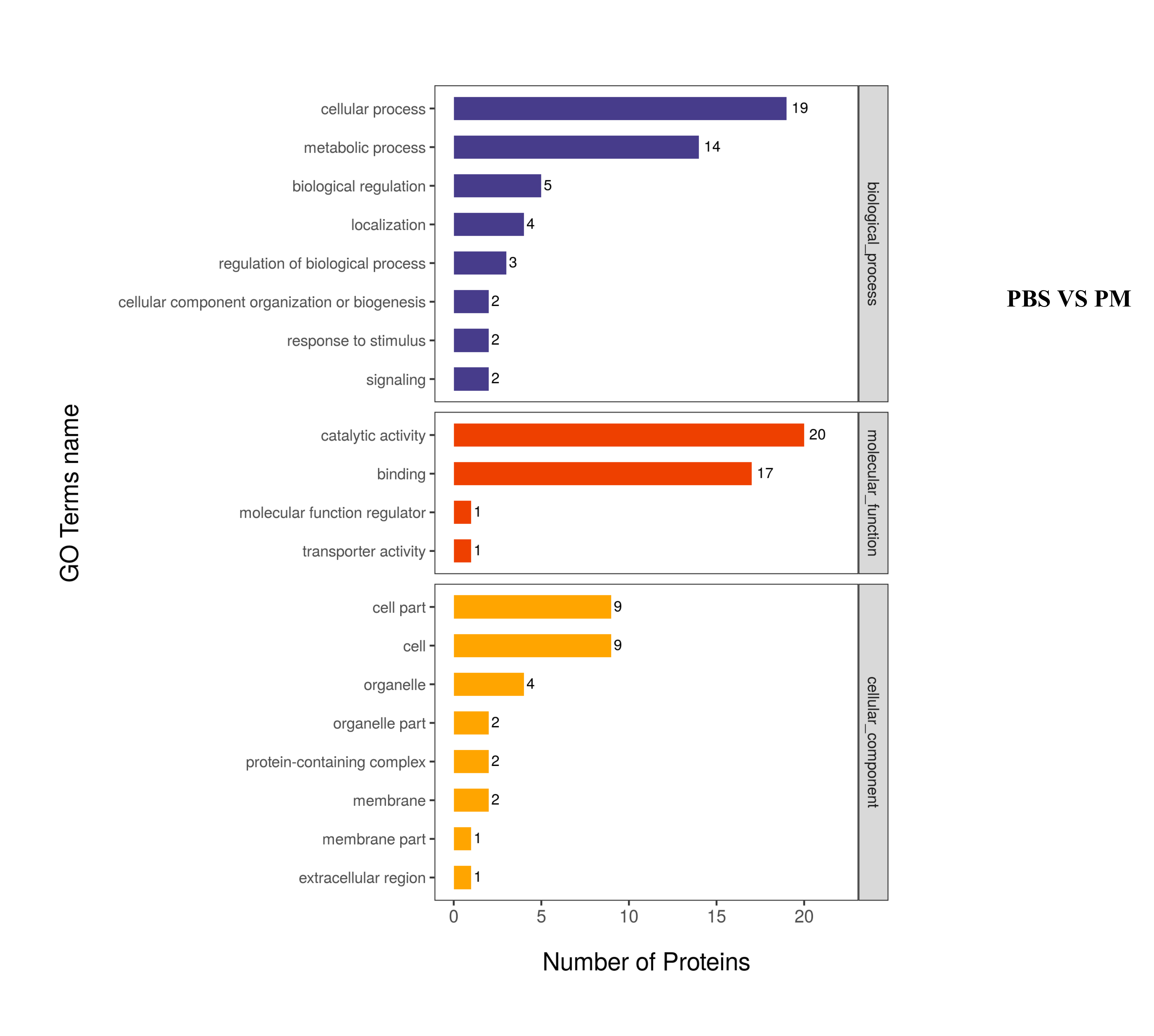

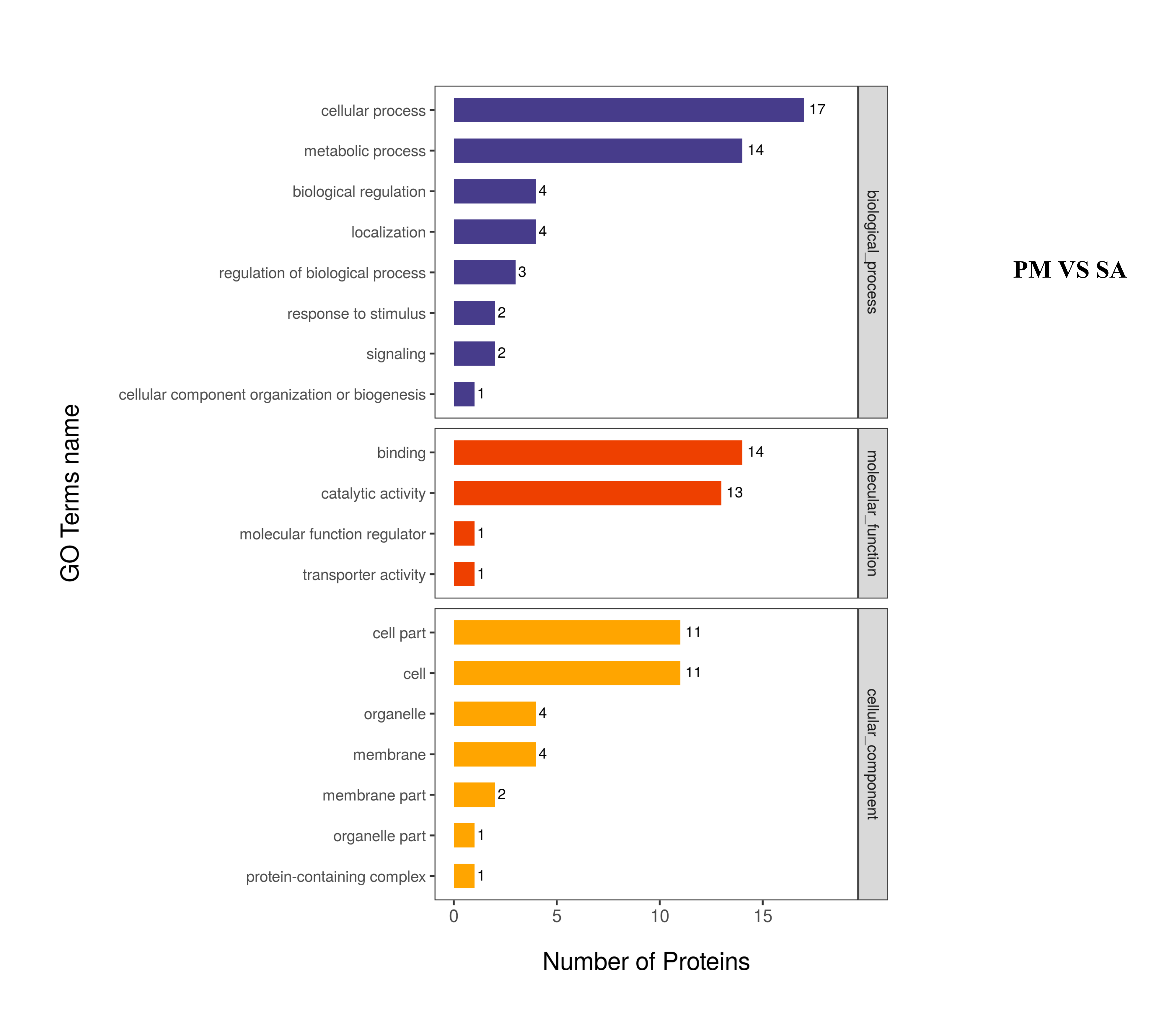

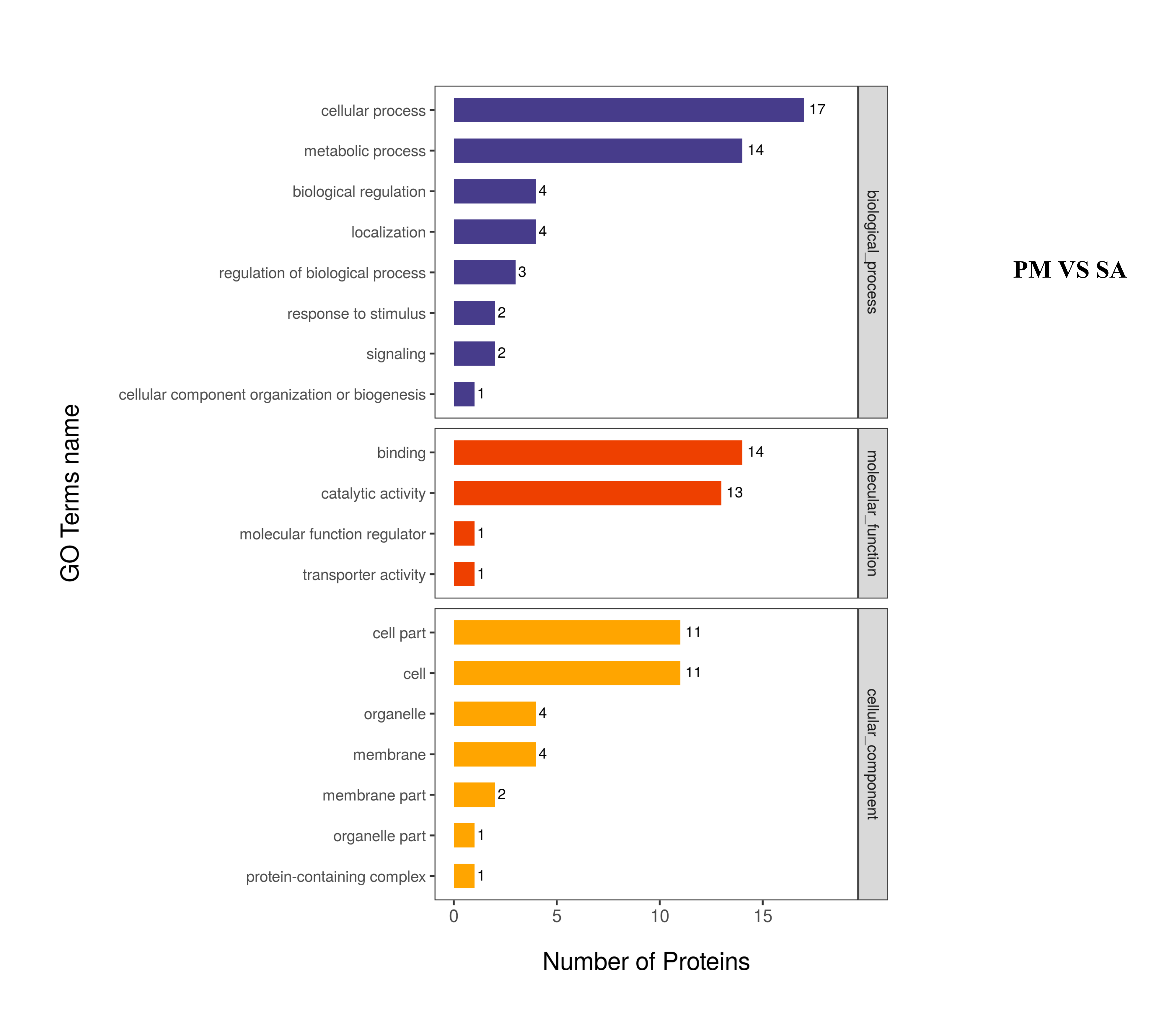


**Supplementary Figure 8.** GO annotation statistics of differentially expressed proteins (DEPs).


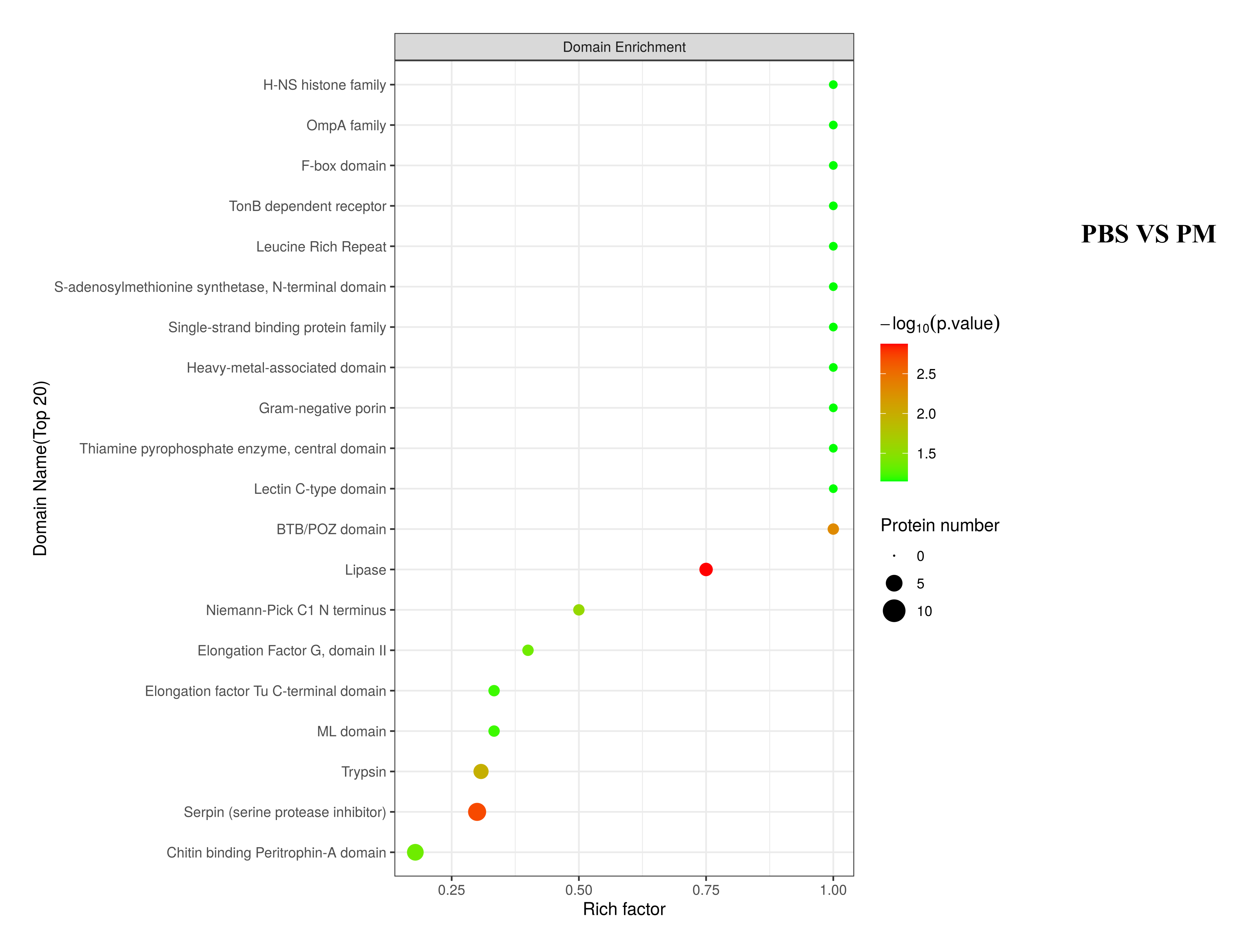

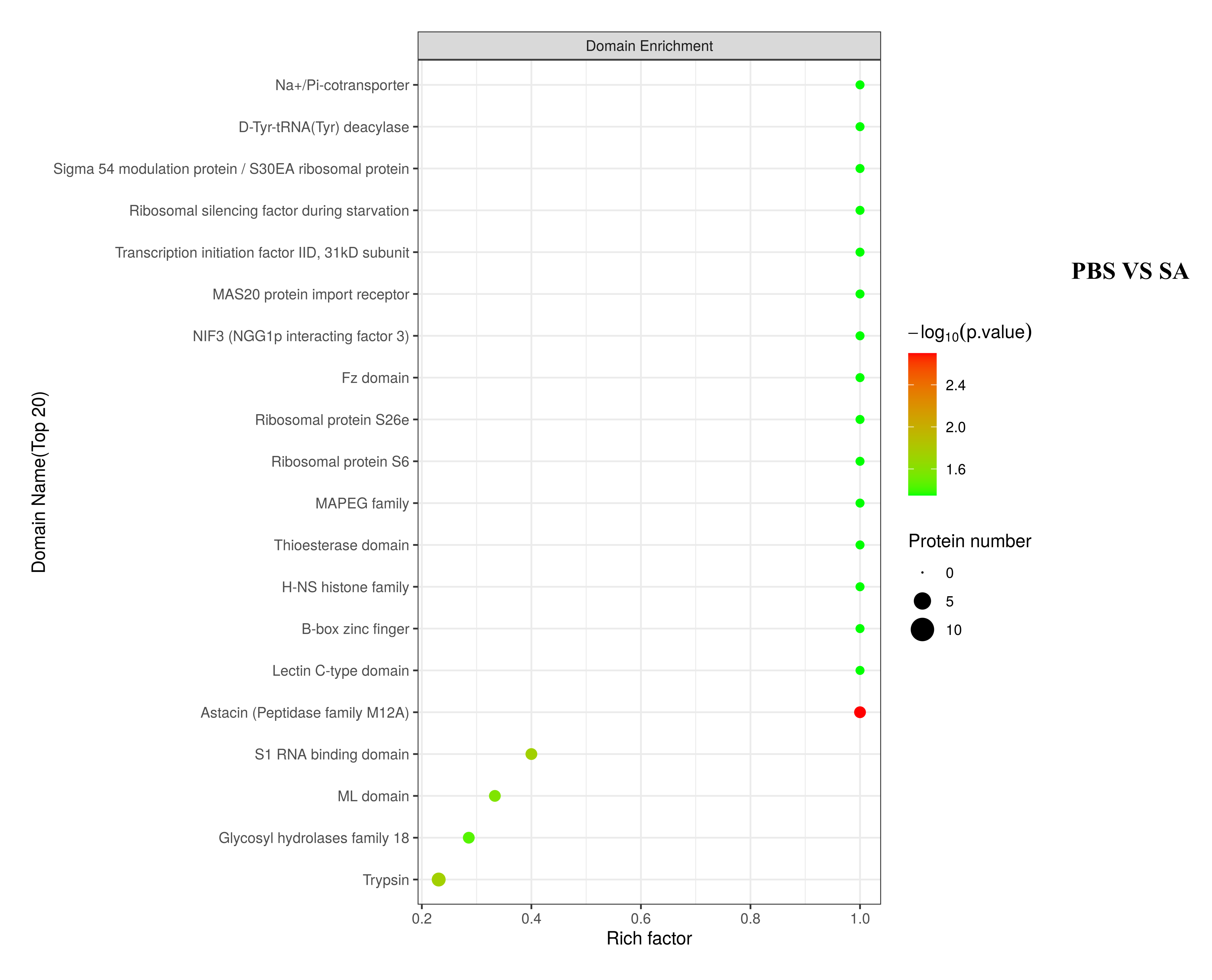

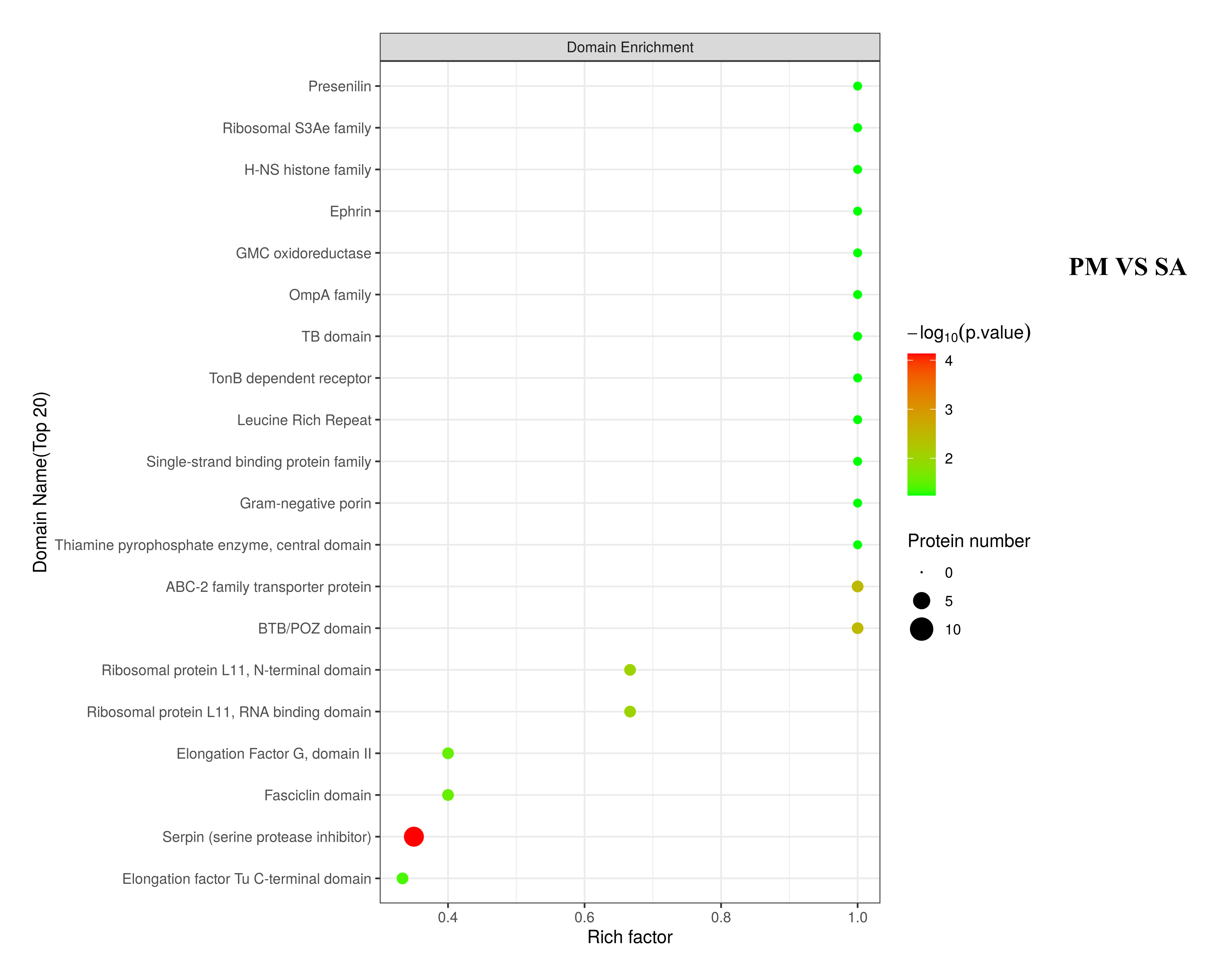


**Supplementary Figure 9.** Domain enrichment analysis of differentially expressed proteins (DEPs).

## 2.2 Supplementary Tables

**Supplementary Table 1.** Specific Primers and Amplification System for RT-qPCR Validation of RNA-seq Results.

| **Primer pairs** | **(5’ to 3’ end)** | **Anotation** | **PCR Product** | **Amplification system** |
| --- | --- | --- | --- | --- |
| Defensin1-RT-F  Defensin1-RT-R | TATTATTCGTCACCATCATCA  TACTGTAGCGAACCACTGAG | Defensin | 110 | 20 μl reaction, 2 × qPCR Mix 10 μl, 1μl each for forward and reverse primers, 1 μl of sample cDNA, 7 μl of ddH_2_O. |
| Defensin2-RT-F  Defensin2-RT-R | GTTTCTTCCACAGGTCGC  CCGTGGAGGCTTCTGTAG | Defensin | 95 |  |
| Microplusin1-RT-F  Microplusin1-RT-R | CGAACTTTCACGCATACACC  AGGAAATCCGTCCCTCTTG | Microplusin | 120 |  |
| Microplusin2-RT-F  Microplusin2-RT-R | GTGTACTGTGCGATGTCTGGG  AAAGTGCCGTTGATTGATTGA | Microplusin | 138 |  |
| Lysozyme-RT-F  Lysozyme-RT-R | TAACTGGGTCTGCCTCGTCA  CGTAGTCCACAGTGCCATCC | Lysozyme | 83 |  |
| Techylectin-RT-F  Techylectin-RT-R | TACTAAGGAGCACTGGATTGGA  CAGTATGGTTCGTTAGCAGGAT | Techylectin | 95 |  |
| Ef-1α-RT-F  Ef-1α-RT-R | CCTGCCAACCTGACCACTGA  CCACACACATATCCTCGGCG | Elongation factor 1 alpha | 137 |  |

**Supplementary Table 2.** Annotation summary with DIAMOND software.

| **Database** | **Number** | **Ratio (%)** |
| --- | --- | --- |
| NR | 32,059 | 22.19 |
| GO | 12,239 | 8.47 |
| KEGG | 9,078 | 6.28 |
| Pfam | 11,677 | 8.08 |
| EggNOG | 22,306 | 15.44 |
| Swissprot | 14,158 | 9.80 |
| In all database | 5,702 | 3.95 |

**Supplementary Table 3** Tables of Differentially Expressed Genes in the Toll, IMD, MAPK, and NF-κB Signaling Pathways

| **Name** | **PBS** | **PM** | **SA** | **FC**(PM/PBS) | **FC**(SA/PBS) | **Up/Down** | **Pathway** |
| --- | --- | --- | --- | --- | --- | --- | --- |
| TRINITY_DN5977_c1_g2 | 121.632495085008 | 605.7516479 | 482.0522127 | 4.980179413 | 3.935586367 | Up | Toll and Imd signaling pathway |
| TRINITY_DN4991_c0_g1 | 877.0040611 | 3442.769057 | 2322.506018 | 3.925602183 | 2.629791506 | Up |  |
| TRINITY_DN11642_c1_g2 | 321.3889665 | 1034.953237 | 791.0010228 | 3.2202513 | 2.443624862 | Up |  |
| TRINITY_DN3843_c0_g1 | 826.0683821 | 2143.149678 | 3619.579671 | 2.594397419 | 4.352800095 | Up |  |
| TRINITY_DN17024_c0_g2 | 9.703586738 | 30.41498918 | 17.33485334 | 3.134406895 | 1.774187781 | Up |  |
| TRINITY_DN202_c3_g2 | 1171.198463 | 3625.336532 | 4417.799147 | 3.095407522 | 3.745918586 | Up |  |
| TRINITY_DN16209_c0_g1 | 225.2189592 | 476.2132099 | 419.1925895 | 2.114445478 | 1.848539589 | Up |  |
| TRINITY_DN1239_c2_g1 | 1481.711295 | 3080.579084 | 3232.987013 | 2.079068368 | 2.166865471 | Up |  |
| TRINITY_DN1795_c2_g1 | 2211.941742 | 4660.929028 | 4654.164018 | 2.107166269 | 2.090002495 | Up |  |
| TRINITY_DN2869_c0_g1 | 295.1234931 | 822.7886371 | 796.3036123 | 2.787946932 | 2.679817511 | Up |  |
| TRINITY_DN6658_c0_g1 | 1820.766278 | 739.24333 | 1041.958046 | 0.406006712 | 0.568342054 | Down |  |
| TRINITY_DN19901_c1_g1 | 53.95919003 | 16.06135599 | 6.326520261 | 0.29765747 | 0.116416982 | Down |  |
| TRINITY_DN19901_c0_g2 | 18.08986734 | 2.493715445 | 6.466659932 | 0.137851505 | 0.3551392 | Down |  |
| TRINITY_DN17829_c0_g1 | 482.7712372 | 92.74550535 | 63.85327584 | 0.192110669 | 0.131388181 | Down |  |
| TRINITY_DN1251_c7_g1 | 244.3520083 | 2041.662982 | 2525.766157 | 8.35541724 | 10.27274533 | Up | MAPK signaling pathway |
| TRINITY_DN114517_c0_g2 | 2.910453473 | 12.93539279 | 14.76465256 | 4.44445957 | 5.037718584 | Up |  |
| TRINITY_DN991_c0_g1 | 499.0762497 | 1117.908555 | 1138.122147 | 2.23995543 | 2.264589745 | Up |  |
| TRINITY_DN759_c0_g4 | 91.58286956 | 343.0764413 | 276.9954475 | 3.746076563 | 3.005041314 | Up |  |
| TRINITY_DN1224_c1_g1 | 677.9905697 | 1375.867062 | 1313.144187 | 2.029330677 | 1.923285843 | Up |  |
| TRINITY_DN1261_c2_g1 | 161.8217343 | 7488.920356 | 3866.669308 | 46.27882892 | 23.72152498 | Up |  |
| TRINITY_DN2869_c0_g1 | 295.1234931 | 822.7886371 | 796.3036123 | 2.787946932 | 2.679817511 | Up |  |
| TRINITY_DN6089_c0_g1 | 295.1234931 | 822.7886371 | 796.3036123 | 2.787946932 | 2.679817511 | Up |  |
| TRINITY_DN3180_c0_g1 | 869.2461605 | 1801.128701 | 1842.108557 | 2.072058276 | 2.104607392 | Up |  |
| TRINITY_DN514_c2_g1 | 365.8399951 | 1477.6387 | 1724.93152 | 4.039029958 | 4.682831514 | Up |  |
| TRINITY_DN7115_c1_g2 | 1466.27145 | 4241.048501 | 2506.386779 | 2.892403382 | 1.6976213 | Up |  |
| TRINITY_DN80_c1_g1 | 167.3129072 | 400.6363911 | 381.4927565 | 2.394533679 | 2.264387166 | Up |  |
| TRINITY_DN16209_c0_g1 | 225.2189592 | 476.2132099 | 419.1925895 | 2.114445478 | 1.848539589 | Up |  |
| TRINITY_DN8189_c0_g1 | 51.10075839 | 1602.175931 | 1263.350039 | 31.35327111 | 24.5281912 | Up |  |
| TRINITY_DN7115_c1_g1 | 92.06383803 | 204.9201173 | 117.1710998 | 2.225848082 | 1.263892801 | Up |  |
| TRINITY_DN4879_c1_g1 | 212.0875494 | 1606.517376 | 1124.536664 | 7.574784003 | 5.26542174 | Up |  |
| TRINITY_DN3578_c0_g1 | 2066.451253 | 8032.641309 | 6133.944502 | 3.887167092 | 2.948120286 | Up |  |
| TRINITY_DN1665_c0_g1 | 215.2087664 | 603.2776215 | 708.2945427 | 2.80322048 | 3.269318568 | Up |  |
| TRINITY_DN13487_c0_g1 | 855.2650348 | 2907.445904 | 2468.503857 | 3.399467751 | 2.866069228 | Up |  |
| TRINITY_DN3335_c0_g1 | 338.0692738 | 686.1936715 | 663.7306952 | 2.029742792 | 1.949840975 | Up |  |
| TRINITY_DN27650_c0_g1 | 7.446851096 | 37.65283518 | 14.23976454 | 5.056208953 | 1.898196239 | Up |  |
| TRINITY_DN11234_c0_g1 | 11.87023007 | 31.24154859 | 26.80379065 | 2.631924437 | 2.242285517 | Up |  |
| TRINITY_DN3888_c1_g1 | 123.5663271 | 257.3975044 | 252.6759313 | 2.083071582 | 2.031378544 | Up |  |
| TRINITY_DN21150_c0_g1 | 2.923106078 | 24.24479545 | 17.99865098 | 8.294189402 | 6.114069893 | Up |  |
| TRINITY_DN4991_c0_g1 | 877.0040611 | 3442.769057 | 2322.506018 | 3.925602183 | 2.629791506 | Up |  |
| TRINITY_DN349_c0_g1 | 98.40636444 | 347.5374937 | 304.3949722 | 3.531656674 | 3.071940233 | Up |  |
| TRINITY_DN18506_c0_g1 | 7.862080647 | 24.09737305 | 16.93447567 | 3.065012194 | 2.138492917 | Up |  |
| TRINITY_DN149142_c0_g1 | 127.5222857 | 47.98858015 | 56.93572801 | 0.376315245 | 0.443431881 | Down |  |
| TRINITY_DN47443_c0_g1 | 124.4593478 | 16.90568457 | 25.39046976 | 0.135832984 | 0.202606504 | Down |  |
| TRINITY_DN14917_c0_g1 | 43.43314366 | 11.20991309 | 9.177387765 | 0.258095826 | 0.20992554 | Down |  |
| TRINITY_DN7667_c0_g1 | 190.4558961 | 53.82701077 | 34.1064213 | 0.282621919 | 0.177955387 | Down |  |
| TRINITY_DN2859_c0_g1 | 602.9240484 | 154.6038289 | 145.2927261 | 0.256423391 | 0.239260434 | Down |  |
| TRINITY_DN1977_c1_g1 | 3264.751848 | 1046.39731 | 892.7925662 | 0.320513582 | 0.271610727 | Down |  |
| TRINITY_DN4879_c1_g1 | 212.0875494 | 1606.517376 | 1124.536664 | 7.574784003 | 5.26542174 | Up | NF-kappa B signaling pathway |
| TRINITY_DN1665_c0_g1 | 215.2087664 | 603.2776215 | 708.2945427 | 2.80322048 | 3.269318568 | Up |  |
| TRINITY_DN21150_c0_g1 | 2.923106078 | 24.24479545 | 17.99865098 | 8.294189402 | 6.114069893 | Up |  |
| TRINITY_DN114517_c0_g2 | 2.910453473 | 12.93539279 | 14.76465256 | 4.44445957 | 5.037718584 | Up |  |
| TRINITY_DN1251_c7_g1 | 244.3520083 | 2041.662982 | 2525.766157 | 8.35541724 | 10.27274533 | Up |  |
| TRINITY_DN2869_c0_g1 | 295.1234931 | 822.7886371 | 796.3036123 | 2.787946932 | 2.679817511 | Up |  |
| TRINITY_DN16209_c0_g1 | 225.2189592 | 476.2132099 | 2.114445478 | 419.1925895 | 1.848539589 | Up |  |
| TRINITY_DN80_c1_g1 | 167.3129072 | 400.6363911 | 2.394533679 | 381.4927565 | 2.264387166 | Up |  |
| TRINITY_DN4100_c0_g1 | 331.249005 | 150.3705008 | 147.7128399 | 0.453950045 | 0.442804274 | Down |  |
| TRINITY_DN2859_c0_g1 | 602.9240484 | 154.6038289 | 145.2927261 | 0.256423391 | 0.239260434 | Down |  |
| TRINITY_DN5575_c1_g1 | 370.6327997 | 184.2661808 | 185.3050749 | 0.497166416 | 0.496483185 | Down |  |
| TRINITY_DN149142_c0_g1 | 127.5222857 | 47.98858015 | 56.93572801 | 0.376315245 | 0.443431881 | Down |  |
| TRINITY_DN14917_c0_g1 | 43.43314366 | 11.20991309 | 0.258095826 | 9.177387765 | 0.20992554 | Down |  |
| TRINITY_DN47443_c0_g1 | 124.4593478 | 16.90568457 | 25.39046976 | 0.135832984 | 0.202606504 | Down |  |

**Supplementary Table 4** Tables of Key Immune Molecules Identified at the Transcriptional Level.

Upload as an EXCEL table separately.

**Supplementary Table 5** Table of Protein Network Interaction Statistics. Upload as an EXCEL table separately.

**Supplementary Table 6** Differentially Expressed Genes with Transcriptional-Translational Upregulation Concordance. Upload as an EXCEL table separately.

**Supplementary Table 7.** Identification and statistical table of immune defense related proteins.

Upload as an EXCEL table separately.

**Supplementary Table 8.** *Hyalomma anatolicum* differentially up-regulates innate immune related genes.

| **Name** | **PBS** | **PM** | **SA** | **FC**(PM/PBS) | **FC**(SA/PBS) | **Up/Down** | **Annotation** |
| --- | --- | --- | --- | --- | --- | --- | --- |
| TRINITY_DN1272_c2_g1 | 25,566.45014 | 158,724.9001 | 185,790.5137 | 6.208327681 | 7.215043403 | Up | microplusin |
| TRINITY_DN1760_c1_g1 | 1,379.590463 | 4,250.803851 | 4,526.518795 | 3.081207042 | 3.25697176 | Up | microplusin |
| TRINITY_DN6219_c1_g2 | 23,780.68118 | 184,672.364 | 212,332.5867 | 7.765646519 | 8.866549429 | Up | microplusin |
| TRINITY_DN1611_c0_g1 | 329.3018605 | 19,756.88664 | 20,757.51382 | 59.99628005 | 62.53112359 | Up | defensin |
| TRINITY_DN249_c0_g1 | 566.4901001 | 3,948.816157 | 3,726.290063 | 6.970671079 | 6.531400395 | Up | defensin |
| TRINITY_DN7431_c2_g2 | 2.018650623 | 349.4052127 | 386.2707364 | 173.0885021 | 189.7365211 | Up | defensin |
| TRINITY_DN1180_c6_g1 | 7.594843801 | 51.87069646 | 31.63734781 | 6.829725247 | 4.136085742 | Up | lectin |
| TRINITY_DN1606_c1_g1 | 127.8281008 | 720.8050463 | 1,158.156893 | 5.638862203 | 9.004271681 | Up | lectin |
| TRINITY_DN40701_c0_g1 | 289.6115711 | 2,096.757691 | 1,695.158717 | 7.239896124 | 5.812711406 | Up | lectin |
| TRINITY_DN937_c0_g1 | 2,250.946066 | 10,360.00647 | 8,389.2055 | 4.602512083 | 3.701012926 | Up | lectin |
| TRINITY_DN6583_c0_g1 | 43.58199922 | 158.6968867 | 101.2250071 | 3.641340221 | 2.307646642 | Up | lectin |
| TRINITY_DN1110_c0_g2 | 17.70677338 | 104.9402699 | 107.086401 | 5.926560852 | 6.002980881 | Up | lectin |
| TRINITY_DN1476_c0_g1 | 2,518.79228 | 5,167.272281 | 5,994.315694 | 2.051488058 | 2.363593525 | Up | lysozyme |
| TRINITY_DN49576_c0_g1 | 58.96546623 | 116.8373343 | 126.2274936 | 1.995526044 | 2.140702036 | Up | lysozyme |
